# Reporting and methodological quality of systematic literature reviews evaluating the associations between e-cigarette use and cigarette smoking behaviors: a systematic quality review

**DOI:** 10.1186/s12954-021-00570-9

**Published:** 2021-11-27

**Authors:** Mimi M. Kim, Lynley Pound, Isabella Steffensen, Geoffrey M. Curtin

**Affiliations:** 1 Scientific and Regulatory Affairs, RAI Services Company, 401 North Main Street, Winston-Salem, NC 27101 USA; 2 Thera-Business, Kanata, ON Canada

**Keywords:** E-cigarettes, Methodological quality, PRISMA, AMSTAR 2

## Abstract

**Introduction:**

Several published systematic reviews have examined the potential associations between e-cigarette use and cigarette smoking, but their methodological and/or reporting quality have not yet been assessed. This systematic quality review followed Preferred Reporting Items for Systematic Reviews and Meta-Analyses (PRISMA) guidelines and AMSTAR (A MeaSurement Tool to Assess systematic Reviews) 2 to evaluate the quality of systematic reviews investigating potential associations between e-cigarette use and cigarette smoking.

**Materials and methods:**

PubMed/MEDLINE, Embase, and PsycINFO were searched from 01 January 2007 to 24 June 2020. Methodological quality was assessed using AMSTAR 2, and reporting quality was assessed using PRISMA guidelines.

**Results:**

Of 331 potentially relevant systematic reviews, 20 met predefined inclusion criteria. Most reviews (*n* = 15; 75%) reported on e-cigarette use and cigarette smoking cessation, while three reported on e-cigarette use and cigarette smoking initiation (15%); and two reported on cigarette smoking initiation and cessation (10%). According to AMSTAR 2 guidelines, 18 of the 20 reviews (90%) were “critically low” in overall confidence of the results, while two were ranked “low.” Additionally, reporting quality varied across the reviews, with only 60% reporting at least half of the PRISMA items.

**Discussion:**

Methodological limitations were identified across reviews examining potential associations between e-cigarette use and cigarette smoking behaviors, indicating that findings from these reviews should be interpreted with caution.

**Conclusions:**

Future systematic reviews in this field should strive to adhere to AMSTAR 2 and PRISMA guidelines, to provide high quality syntheses of the available data with transparent and complete reporting.

**Supplementary Information:**

The online version contains supplementary material available at 10.1186/s12954-021-00570-9.

## Introduction

### E-cigarettes and their public health impact

E-cigarette use has increased significantly over the past decade, as the prevalence of combustible cigarette smoking has declined [1–7]. Unlike combustible cigarettes, e-cigarettes vaporize solutions that users then inhale; this inhaled vapor does not contain most of the approximately 7000 chemicals present in cigarette smoke, and the evidence suggests that, consequently, e-cigarettes confer fewer health risks than cigarettes [8]. However, e-cigarette users are still exposed to nicotine and other harmful or potentially harmful constituents [9, 10].

The effect of e-cigarette use on cigarette smoking behaviors has become a highly controversial issue [11–13], and an objective examination of this relationship is critical to understanding the inherent risks and benefits presented by e-cigarette use [10]. Among the public health concerns regarding e-cigarette use is that e-cigarettes serve as a gateway to combustible cigarette smoking and dependency, particularly in youth; and that existing cigarette smokers who adopt the use of e-cigarettes will be diverted from quitting cigarette smoking [10]. However, others contend that e-cigarette use may instead help promote cessation or reduction of cigarette smoking [11]. Recently, US regulators have urged the tobacco control field to “pay close attention to the effects of e-cigarette use on initiation of and cessation from combustible tobacco use, regardless of the effects of e-cigarettes on health outcomes” [10].

Determining whether the risks that may be associated with the use of e-cigarettes offset the benefits expected from reduced cigarette smoking is crucial for ensuring the adoption of appropriate regulatory policies. Ultimately, an assessment of causality is central to understanding the overall public health effect of e-cigarettes. Coincident with the growing popularity of e-cigarettes, there have been a number of systematic reviews conducted to address these questions.

### Overview of systematic review evaluation

A systematic review is a methodologically rigorous tool for summarizing and evaluating the best evidence available for healthcare interventions. Systematic reviews comprehensively collect, then critically synthesize and evaluate, evidence relating to a given research question. Meta-analyses, which are often included in systematic reviews, combine evidence from multiple studies to provide a comprehensive statistical estimate with greater power than the individual studies alone. Systematic reviews are an invaluable—and increasingly used—tool for informing healthcare professionals and policy makers on evidence-based healthcare decisions [14]. Reviews are required to consider the quality of evidence including the risk of bias, indirectness, inconsistency, imprecision, and the likelihood of publication and reporting bias to provide a transparent, replicable, and systematic synthesis of available evidence [14]. The construct of the “quality of evidence” measures the degree of confidence that the estimate of an effect can adequately support a particular decision or recommendation [14, 15]. However, a wide variation of quality across systematic reviews—with a high prevalence of reviews of poor methodological rigor—has been reported across a number of fields, undermining the confidence in the evidence that would be required for effective decision-making [16–20]. Given the important role systematic reviews play in informing policy decisions and recommendations, coupled with the inconsistency in their general quality, it is vital that the quality of systematic reviews is adequately assessed.

A number of assessment tools have been developed to evaluate and improve the methodological and reporting quality of systematic reviews and meta-analyses. First published in 2007, the AMSTAR (A MeaSurement Tool to Assess systematic Reviews) tool was developed collaboratively with the intent to produce a practical critical appraisal tool that allowed individuals who may not have advanced epidemiological training to carry out rapid and reproducible study quality assessments; it has since become one of the tools most often endorsed as the preferred tool for the quality assessment of systematic reviews by methods groups[21–23]. AMSTAR was updated in 2017 (renamed AMSTAR 2) [24], with the most significant change implemented being the inclusion of an assessment for non-randomized studies of intervention effects (NRSIs), in addition to randomized controlled trials (RCTs). AMSTAR 2 is a 16-item checklist that critically appraises the methodological quality of systematic reviews, including the quality of study selection and data extraction, and the suitability of methods used for data analysis and assessment of scientific quality. While AMSTAR 2 assesses the manner in which the review is conducted, this tool does not assess whether the reporting is transparent and complete. This highlights the need for additional tools to evaluate the reporting quality of systematic reviews.

The PRISMA (Preferred Reporting Items for Systematic Reviews and Meta-Analyses) statement can be used in conjunction with AMSTAR 2 to assist in the review process. The PRISMA statement was developed in 2009 and is a revision of previously-published guidelines—the QUOROM statement (QUality Of Reporting Of Meta-analyses)—which were developed by an international group that sought to address suboptimal reporting in meta-analyses of randomized controlled trials [25]. PRISMA aims to improve standards of reporting of systematic reviews using a 27-item checklist, divided into seven sections—including title, abstract, introduction, methods, results, discussion, and funding—and determines whether the review explicitly reports each item in the corresponding section [25].

### Objective

To date, neither the number of systematic reviews examining the association between e-cigarette use and cigarette smoking, nor the methodological quality or reporting characteristics of those reviews, has been systematically assessed. Given the potential for these systematic reviews to inform both healthcare professionals and policy makers on evidence-based healthcare decisions, it is important that the quantity and quality of these reviews be critically assessed to ensure that key decisions are being made with the best possible available evidence. Accordingly, this systematic quality review evaluation examined the quantity and quality of evidence within this evidence base of systematic reviews. Specifically, systematic reviews that compared the impact of e-cigarettes (nicotine-free and/or nicotine-containing) with any relevant comparator on cigarette smoking behaviors in youth, young adults, and/or adults were included in this systematic quality review. Once relevant systematic reviews were identified, their methodological and reporting quality was determined using AMSTAR 2 and PRISMA guidelines.

## Materials and methods

The current systematic quality review protocol followed PRISMA guidelines for best practices in systematic reviews [26], and was prospectively registered with the PROSPERO registry for systematic reviews (CRD42018078252). The protocol can be found at: https://www.crd.york.ac.uk/PROSPERO/display_record.php?RecordID=78252. There were no significant deviations from the protocol.

### Key questions

The key research questions represent the main focus of the review, which was to evaluate the reporting and methodological quality of published systematic reviews on the associations between e-cigarette use and cigarette smoking behaviors.

**Key Question 1**: What is the methodological quality of published systematic reviews examining e-cigarette use and cigarette smoking behaviors, as assessed by the AMSTAR 2 scale?

**Key Question 2**: What is the reporting quality of published systematic reviews examining e-cigarette use and cigarette smoking behaviors, as assessed by PRISMA guidelines?

### Literature search

A comprehensive search of PubMed/MEDLINE, Embase, and PsycINFO was performed by an information specialist, applying search terms developed using medical subject headings (MeSH) and text words related to the associations between e-cigarette use and cigarette smoking behaviors. The applied search date parameters were from 01 January 2007 to 24 June 2020. An initial database search was conducted on 18 September 2019 and included systematic reviews published from 01 January 2007 to 18 September 2019. An updated database search was conducted on 24 June 2020, and included systematic reviews published from 01 January 2019 to 24 June 2020. Since searches are best conducted from the first of the year, an overlap in the two search timeframes was unavoidable. The search dates were restricted to exclude articles prior to 2007, as defined by the US market introduction of e-cigarettes [27, 28]. Reference lists of included systematic reviews were also checked, and a gray literature search of Google Scholar was performed to identify any additional relevant systematic reviews. In addition, content experts in the field were consulted. Trial/study registries were not searched, as we were specifically interested in published systematic reviews. Detailed information on the literature search strategies (both initial and update searches) are provided in Additional file 1: APPENDIX 1: Search strategy.

### Inclusion criteria

Search results were screened with inclusion/exclusion criteria developed using the PICOS (Population or participants and conditions of interest, Interventions or exposures, Comparisons or control groups, Outcomes of interest, and Study designs) review method [29]. The domain under study was the methodological and reporting quality—as evaluated by AMSTAR 2 and the PRISMA statement—of published systematic reviews on the potential associations between e-cigarette use and cigarette smoking behaviors, such as initiation of cigarette smoking and cessation (or reduction) of cigarette smoking. Additionally, we sought to evaluate the quantity of available reviews.

The following predefined inclusion criteria were used:

**Table:** 

**P**opulation or participants and conditions of interest	Youth, young adults, and/or adults who are cigarette or non-cigarette smokers
**I**nterventions or exposures	Nicotine-free and/or nicotine-containing e-cigarettes. We did not distinguish between open and closed e-cigarette systems
**C**omparisons or control groups	All relevant comparators to an e-cigarette intervention. Systematic reviews were not restricted based on the comparator
**O**utcomes of interest	The primary objective of this assessment was to determine the reporting and methodological quality of previously published systematic reviews using the PRISMA statement and AMSTAR 2. Additionally, this assessment sought to evaluate the quantity of available evidence
**S**tudy designs	Systematic reviews with or without meta-analyses published in English were included. Any study containing the words “systematic review,” with or without “meta-analysis,” or if the authors identified the study as such in the title or abstract was included. Additionally, any study that described a systematic approach to searching the literature, identifying and selecting studies, and synthesizing the available evidence was also included

### Exclusion criteria

The following exclusion criteria were predefined:

1. Articles that are not systematic reviews with or without meta-analyses.
2. Articles that do not assess the associations between e-cigarette use and combustible cigarette smoking behaviors.
3. Articles with date of publication outside the reporting period.
4. Articles in which the full text is non-English.
5. Duplicate articles.

### Data management

**Fig. 1:**
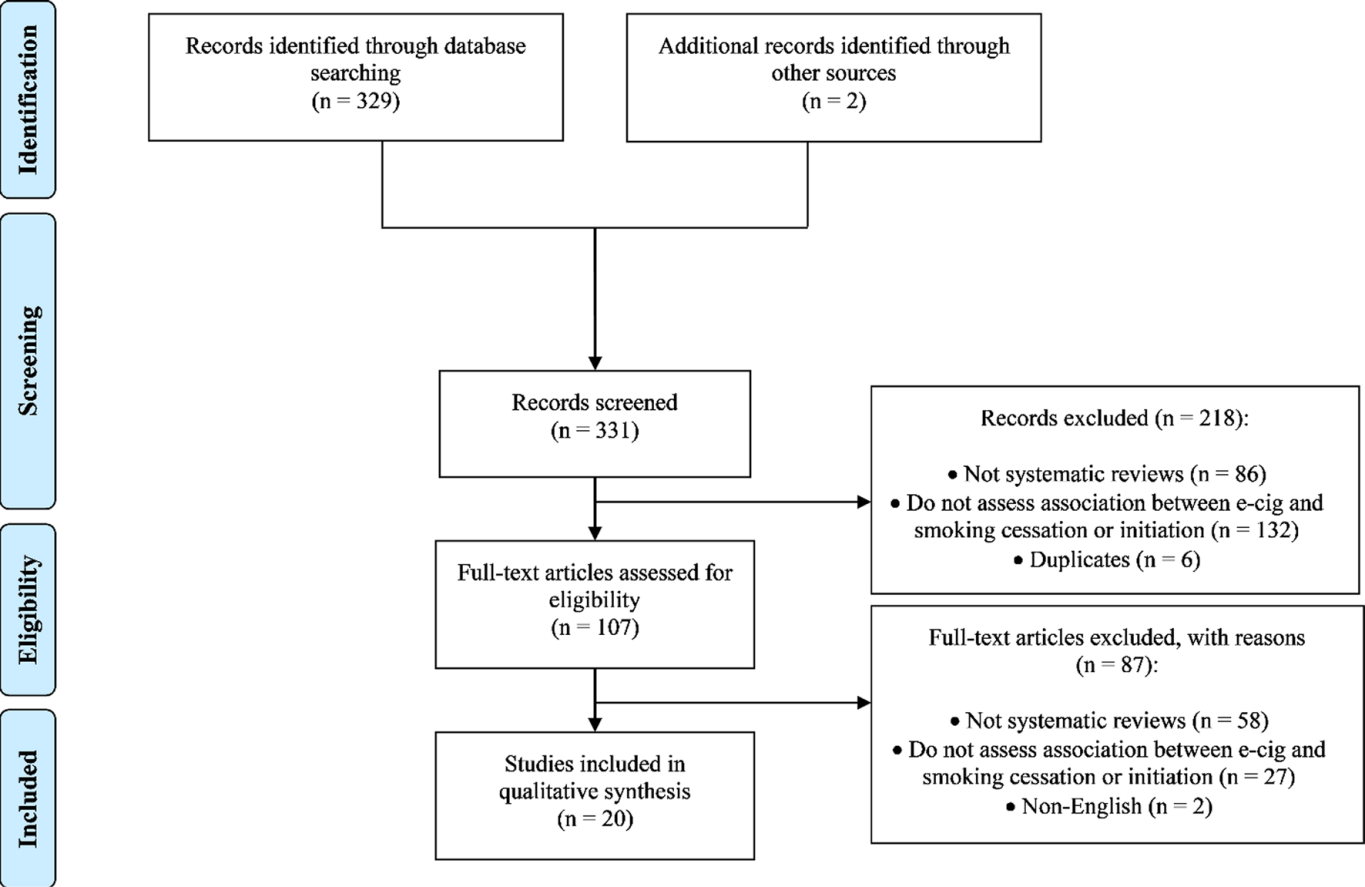
PRISMA Flow Diagram for Systematic Review of the Quality of Systematic Reviews

The search results were uploaded and maintained in DistillerSR (Evidence Partners, Ottawa, Ontario), a web-based systematic review software program. DistillerSR allows reviewers to screen the search results online, extract data from relevant systematic reviews, perform quality assessments online, and export results. Screening questions based on the inclusion and exclusion criteria specified in the systematic quality review protocol were uploaded to DistillerSR. Details of the number of articles identified and examined are provided in the PRISMA flow diagram (Fig. 1) [25].

### Study selection process

Articles were initially screened at the title and abstract level, and full-text articles were obtained for any articles that could not be excluded based on the initial screening alone. Two reviewers independently screened the articles, based on the selection criteria, and any discrepancies between the two reviewers regarding articles that were included or excluded were discussed and resolved in a meeting between reviewers, with co-project leads as moderators; a joint decision was made on whether the article should be included or excluded. Reasons for excluding an article were documented.

### Data extraction

Data were independently extracted by one research scientist (LP) and checked by a second research scientist (IS). Discrepancies were identified and resolved through discussion, including a third team member (MMK/GMC) when necessary. Data extraction forms were created in DistillerSR.

For each systematic review, information regarding the study characteristics and outcomes was extracted, including: author; journal name; publication year; funding source and conflicts of interest (to include both the presence of any conflicts, as well as the nature of any conflicts declared); whether a protocol or guide had been written prior to developing the search strategy; methods and databases searched; inclusion and exclusion criteria; study selection and data extraction methodology; number of systematic reviews included (in both the review and in the meta-analysis, if applicable); list of excluded articles (with justifications); description of included reviews (study setting and duration, population and participant characteristics, interventions, comparators, outcomes, and study design); methods for assessing risk of bias and discussion of any impact on the results; methods of statistical analysis and statistical combinations of results, if applicable; methods for assessing heterogeneity and discussion of any impact on the results; and methods for assessing publication bias and discussion of any impact on the results.

Although the protocol noted that study authors may be contacted if clarification was needed pertaining to any of the extracted data, we did not determine this to be necessary, particularly as our aim was to assess the methodology and reporting quality of existing systematic reviews, and those reviews provided data from published articles. Similarly, because the goal of the current review was to evaluate the methodological and reporting quality of existing systematic reviews, and not the individual studies included in each review, a risk of bias assessment was not relevant and therefore not performed.

### Quality assessment

The methodological quality of included systematic reviews was assessed with the AMSTAR 2 tool [24]. The AMSTAR 2 tool (an update of AMSTAR) is a 16-item critical appraisal tool for systematic reviews that include randomized or nonrandomized studies of healthcare interventions, or both, and includes seven items considered critical domains. Each item in the AMSTAR 2 checklist was evaluated for each systematic review, and labelled “yes,” “no,” “partial yes,” or “not applicable” depending whether or not the requirements of a particular item were satisfied. An “adjusted” percentage that eliminated non-applicable systematic reviews was generated, where appropriate. An overall score was generated to indicate the overall confidence in the results of the review, as previously published [24]. Briefly, the score was generated according to the following criteria:

**Table:** 

Overall Score	Criteria
High	No or one non-critical weakness (i.e., a “no” in no or one non-critical domain)
Moderate	More than one non-critical weakness (i.e., a “no” in more than one non-critical domain) (note: multiple non-critical weaknesses may diminish confidence in the review and it may be appropriate to move the overall appraisal down from moderate to low confidence)
Low	One critical flaw (i.e., a “no” in one critical domain) with or without non-critical weaknesses
Critically Low	More than one critical flaw (i.e., a “no” in more than one critical domain) with or without non-critical weaknesses

The seven domains considered critical were:

- Protocol registered before commencement of the review (item 2)
- Adequacy of the literature search (item 4)
- Justification for excluding individual studies (item 7)
- Risk of bias from individual studies being included in the review (item 9)
- Appropriateness of meta-analytical methods (item 11)
- Consideration of risk of bias when interpreting the results of the review (item 13)
- Assessment of presence and likely impact of publication bias (item 15)

For the purpose of this systematic quality review, we did not consider a “partial yes” in any of the critical domains as a critical flaw.

The reporting quality of included systematic reviews was assessed with the PRISMA statement [25], a 27-item checklist that verifies the transparent and complete reporting of systematic reviews and meta-analyses. Each item in the PRISMA checklist was evaluated for each systematic review, and labelled as “yes,” “no,” or “not applicable” depending whether or not the requirements of a particular item were satisfied.

Both PRISMA and AMSTAR 2 assessments were independently performed in duplicate, and any discrepancies between the two reviewers were discussed and resolved in a meeting between reviewers, including a third reviewer as necessary, to arrive at a joint decision.

## Results

### Study selection

The initial database search (01 January 2007 to 18 September 2019) yielded 244 potentially relevant articles, while the updated database search identified an additional 132 articles; 47 of the articles were duplicate articles across the two searches, due to a resultant overlap from the original and updated search timeframes. Thus, a total of 329 potentially relevant articles were retrieved from the specified databases. By checking the reference lists of these articles and Google Scholar, two additional systematic reviews were identified [30, 31], bringing the total number of potentially relevant articles for this time period to 331.

Exclusion of irrelevant articles (*n* = 224) resulted in 107 potentially relevant articles eligible for full text screening (kappa = 0.814). Of the 107 articles identified for full text review (see Additional file 1: APPENDIX 2 for a list of articles reviewed in full text, with titles and abstracts), 87 did not meet the inclusion criteria. Reasons for exclusion of articles from the search output are described in Additional file 1: APPENDIX 3. Therefore, 20 systematic reviews were eligible for inclusion in this systematic quality review (Fig. 1) [30–49]. The kappa score, measuring level of agreement between the two reviewers, was 0.832. A list of the included systematic reviews can be found in Additional file 1: APPENDIX 4.

### General study characteristics of included systematic reviews investigating e-cigarette use and cigarette smoking behaviors

The characteristics of the included systematic reviews with or without meta-analyses are summarized in Additional file 1: APPENDIX 5. Half of the systematic reviews included a meta-analysis (10 of 20 reviews) [35–39, 41–43, 46, 48].

### Timeframe

Four systematic reviews were published in 2014 [30, 33, 38, 45], three in 2015 [32, 43, 44], four in 2016 [35, 40–42], two in 2017 [36, 37], one in 2018 [39], four in 2019 [31, 34, 46, 49], and two in 2020 [47, 48].

### Region

There was a range of countries represented by the first author of the systematic reviews including the US [30, 31, 37, 42, 45, 49], Canada [32, 33, 40, 41], the United Kingdom (UK) [34, 35, 38, 46, 48], China [39], Australia [43], Italy [44], Brazil (with cross-appointment in Canada) [36], and Saudi Arabia [47].

### Study designs of included systematic reviews

Most systematic reviews included a range of study designs, with both RCTs and NRSIs [31, 33–38, 41–44, 46–49], including two that specifically noted not restricting by study design [37, 46]. Only one systematic review limited the included studies to RCTs [32], while four reviews did not specify study design restrictions [30, 39, 40, 45].

### Additional review characteristics

Ten systematic reviews included only studies in English [31, 32, 34, 40, 43–47, 49], while two systematic reviews included studies in English and one additional language [33, 39]. Seven systematic reviews did not restrict by language [35–38, 41, 42, 48], while one review did not specify any language restrictions [30]. The most common search method used, in addition to the obligatory comprehensive electronic database search, was the review of reference lists across potentially relevant articles [32, 34, 35, 38, 41, 42, 45, 47, 49]. Only six reviews provided a full, but not comprehensive according to AMSTAR 2, search strategy [34–38, 41]; one provided a limited search strategy [42], eleven provided keywords only [30–33, 39, 43–45, 47–49], and two did not report their strategy [40, 46].

### E-cigarette outcomes

The majority of reviews reported on the potential association between e-cigarette use and cigarette smoking cessation [30, 32–36, 38–44, 47, 49]; three reviews reported on e-cigarette use and cigarette smoking initiation [37, 46, 48]; and two reviews reported on e-cigarette use and both cigarette smoking behaviors [31, 45].

Additional information regarding characteristics of the individual systematic reviews can be found in **APPENDICES 6–8**.

### Assessment of methodological quality using AMSTAR 2

**Fig. 2:**
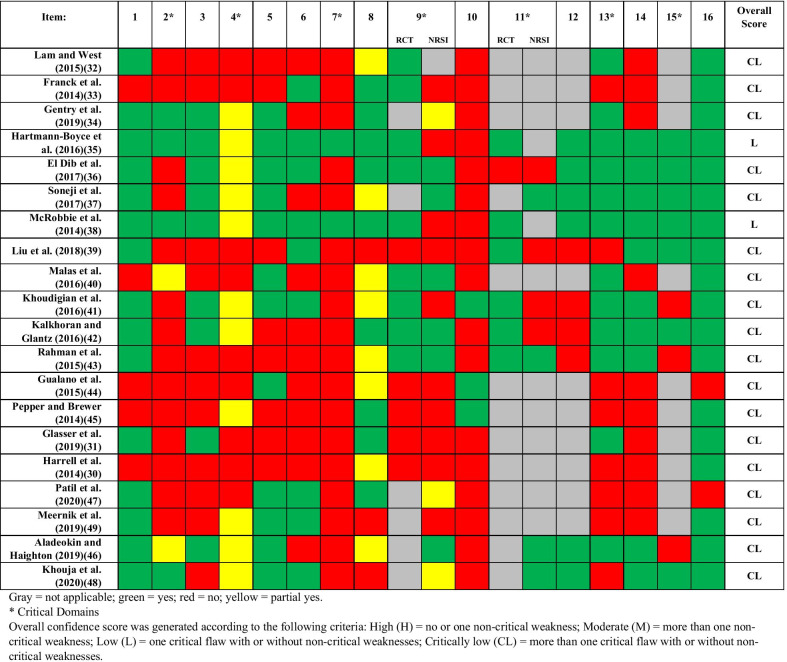
Individual Study Results for Methodological Quality Using the AMSTAR 2 Tool

The results of the AMSTAR 2 assessment of methodological quality for the included systematic reviews are summarized in **APPENDICES 9–10**. Three items (item #s 11, 12, and 15) are specific to meta-analyses and were, therefore, not applicable to 10 reviews [30–34, 40, 44, 45, 47, 49]. Therefore, an “adjusted” percentage, that excluded the 10 systematic reviews without meta-analyses, was generated for these items. Additionally, two items (item #s 9 and 11) were stratified by study design (RCT or NRSI) and were similarly not applicable for a subset of reviews. The results from the AMSTAR 2 assessment of the individual systematic reviews can be found in Fig. 2.

Eighteen systematic reviews scored “critically low” for overall confidence in the results of the review, while two systematic reviews scored “low.” None of the 20 systematic reviews fulfilled all of the 16 AMSTAR 2 quality items.

There were some trends noted across the included systematic reviews. Only 8 of the 16 AMSTAR 2 items were adequately described in approximately half of the systematic reviews. One item varied depending on whether the systematic review included RCTs or NRSIs. Specifically, for Item #9 “Did the review authors use a satisfactory technique for assessing the risk of bias in individual studies that were included in the review?”, 9 of 14 reviews that included RCTs scored a “yes” [32, 33, 35, 36, 38, 40–43] and only 6 of 19 reviews that included NRSIs scored a “yes” [36, 37, 40, 42, 43, 46].

The majority of reviews (75%) failed to report on a quarter of the AMSTAR 2 items (4 items). Among these items, three are considered “critical domains” by the AMSTAR 2 tool guidelines (items 2—Protocol registered before commencement of the review, 4—Adequacy of the literature search, and 7—Justification for excluding individual studies) [24]. The item that was most infrequently provided was a comprehensive literature search strategy (Item 4; 0 of 20 reviews). Although 11 reviews received a “partial yes” in this category (must have searched at least two databases, provided key word and/or search strategy, and justified publication restrictions) [34–38, 41, 42, 45, 46, 48, 49], no authors successfully met all of the criteria for a “yes” in this category (must have also searched the reference lists/bibliographies of included studies, searched trial/study registries, included/consulted content experts in the field, searched for grey literature (where relevant), and conducted the search within 24 months of completion of the review). Additionally, only 4 of 20 reviews contained an explicit statement that the review methods were established prior to the conduct of the review (Item #2: including a justification for any significant deviations from the protocol) [34, 35, 38, 48], only 2 reviews provided a list of excluded studies and justified the exclusions (Item #7) [35, 38], and 3 reviews described the sources of funding for the studies included in the review (Item #10) [41, 44, 45].

The only item that was addressed in the majority of the reviews was related to potential sources of conflict (Item #16: “Did the review authors report any potential sources of conflict of interest, including any funding they received for conducting the review?”) (18 of 20 reviews) [30–43, 45, 46, 48, 49]. For this review, Item #11 (“If meta-analysis was performed did the review authors use appropriate methods for statistical combination of results?”) was stratified by systematic reviews including RCTs and those including NRSIs. Although this item was adequately described in only half of the meta-analyses that included NRSIs, it was adequately described in 6 of 7 six of 7 meta-analyses that included RCTs [35, 38, 39, 41–43].

### Assessment of the quality of reporting using the PRISMA statement

**Fig. 3:**
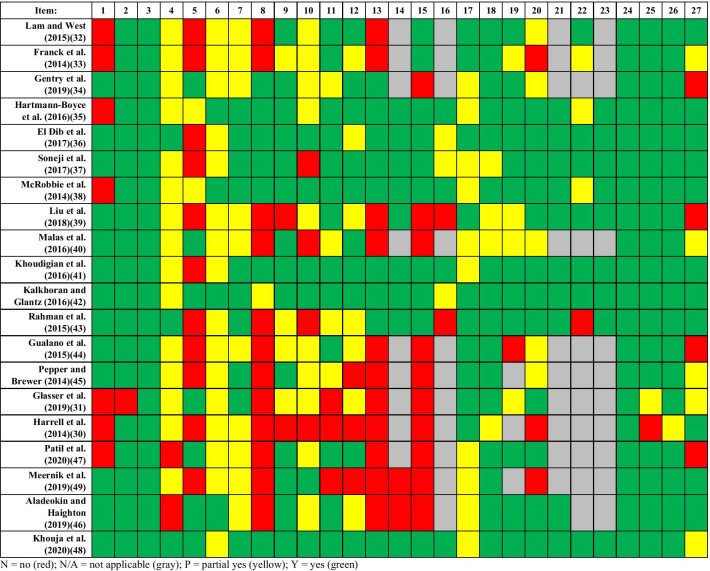
Individual Study Results for Reporting Quality Using the PRISMA Tool

The results of the PRISMA assessment of quality of reporting for systematic reviews are summarized in **APPENDICES 11–12**. An “adjusted” percentage, calculated without including non-applicable reviews, was generated where appropriate. Also, in some instances, the reported percentages are based only on applicable items. “Partial yes” were scored as a “no” for percentage calculations in the following results. The results of the individual systematic reviews can be found in Fig. 3.

Overall, the reporting quality varied between the individual systematic reviews. Of the 20 included systematic reviews, only 12 reviews reported at least half of the PRISMA items. There were some overall trends that are worth noting. Thirteen of 20 reviews identified the report as a systematic review, meta-analysis, or both in the title (Item #1) [34, 36, 37, 39–46, 48, 49], while 19 provided a structured abstract (Item #2) [30, 32–49].

With regard to the Introduction section, all 20 reviews provided a rationale for the review in the context of what is already known (Item #3), while only 3 reviews provided an explicit statement of questions being addressed that contained all components of PICOS (Item #4) [36, 43, 48].

The reporting of the Methods section was similarly varied across the individual systematic reviews. Seven reviews comprehensively reported the items in the Methods section [35–38, 41, 42, 48]. In contrast, 13 reviews poorly reported the Methods section [30–34, 39, 40, 43–47, 49].

There were some trends in the reporting of the Methods section across systematic reviews that are worth noting. Specifically, only seven systematic reviews specified if a review protocol existed and where it could be accessed [31, 34, 40, 42, 46–48]. Additionally, only seven systematic reviews presented a full search strategy such that it could be repeated (35%) [34–38, 41, 48].

Fourteen systematic reviews comprehensively reported the applicable items in the Results section [31, 32, 35–39, 41–43, 45–48]. Further, the same seven reviews that scored well in the reporting of the Methods section also scored well in the reporting of the Results section [35–38, 41, 42, 48]. Six reviews scored poorly on the reporting of the Results section [30, 33, 34, 40, 44, 49].

Generally, the Discussion sections in individual systematic reviews scored well. There were 18 reviews that completely reported all three items in the Discussion section [32–49]. Of the remaining two reviews, one review received a “partial yes” for one item (Item #25) [31], while the second review received a “no” for one item (Item #25) and a “partial yes” for another (Item #26) [30].

Specific to the Discussion section, across the reviews, all of the reviews received a “yes” to Item #24: “Does this review summarize the main findings including the strength of evidence for each main outcome; consider their relevance to key groups (e.g., healthcare providers, users, and policy makers)?”; 18 reviews received a “yes” to Item #25: “Does this review discuss limitations at study and outcome level (e.g., risk of bias), and at review-level (e.g., incomplete retrieval of identified research, reporting bias)?” [32–49]; and, 19 reviews received a “yes” to Item #26: “Does this review provide a general interpretation of the results in the context of other evidence, and implications for future research?” [31–49].

Funding for the systematic review and the role of the funders was reported in 11 of 20 reviews [30, 32, 35–38, 41–43, 46, 49].

## Discussion

Systematic reviews and meta-analyses are increasingly being published and used to inform regulatory decisions, inform health professionals on best practices, and direct research priorities. It is, therefore, critical that systematic reviews be evaluated for their adherence to the methodological guidelines that were defined to increase confidence in the precision and consistency of effects in an evidence base [14]. Without such evaluation, it is impossible to understand the extent to which a systematic review can accurately inform decisions and/or policies.

Importantly, this systematic quality review only identified systematic reviews of “low” and “critically low” methodological quality. This lack of properly conducted and reported systematic reviews investigating whether e-cigarette use is associated with cigarette smoking initiation and/or cessation, as well as the absence of systematic reviews investigating the association between e-cigarette use and other cigarette smoking behaviors, underscores the need for additional high-quality systematic reviews to be performed in this area.

### Deficiencies in methodological quality of systematic reviews using AMSTAR 2

The conclusions from the AMSTAR 2 assessment indicated that the overall methodological quality was poor. Eighteen of the 20 systematic reviews scored “critically low” in the overall confidence in the results of the review, indicating that these systematic reviews should not be relied on to provide an accurate and comprehensive summary of the available studies. Additionally, two systematic reviews received a score of “low” in the overall confidence in the results of the review, indicating that these reviews have a critical flaw and may not provide an accurate and comprehensive summary of the available studies that address the question of interest. Half of the 16 AMSTAR 2 items were described in 50% or fewer reviews.

The deficiency trends across these systematic reviews were important. Specifically, no reviews comprehensively described the literature search strategy (although just over half of the reviews received a “partial yes” in this category); only four reviews provided a reference to a protocol; and only two reviews provided a list of excluded studies with justification. Notably, all three of these poorly-scored items are considered “critical domains” according to the authors of AMSTAR 2 [24].

Assessment of risk of bias was generally lacking based on the AMSTAR 2 tool. Although authors generally adequately assessed risk of bias for RCTs (scored “yes” in 64% of reviews), risk of bias methodology for NRSIs was lacking in the majority of reviews (scored “yes” in only 32% of reviews). In addition, only 15% of systematic reviews described funding sources for the studies included in their review. It has been established that industry-sponsored studies are more likely to report results favoring the sponsored product and are also less likely to be published [50–53]. Funding sources may introduce bias, thereby potentially skewing the results of the individual studies. Thus, this information should be transparently described. It is worth noting that publication bias was, in general, challenging to assess by the included systematic reviews, since it would normally require a minimum of 10 studies, underscoring the lack of studies published in this field. Taken together, improved methodology for assessing risk of bias in systematic reviews investigating e-cigarette use and cigarette smoking behaviors is required.

Additionally, only two of the 20 systematic reviews included in this systematic quality review were published in the Cochrane Database of Systematic Reviews. The Cochrane Database of Systematic Reviews is known for having developed a number of systematic review methodological standards, as well as having a rigorous review process. As such, the systematic reviews published in the Cochrane Database are generally expected to be of higher methodological quality. However, despite this, the two systematic reviews included in our systematic quality review that were published in the Cochrane Database still scored “low” in the overall confidence in the results, according to the AMSTAR 2 tool. These findings are not unexpected. Previous reviews have found that though the methodological quality of Cochrane reviews may be superior to non-Cochrane reviews, the methodological quality of Cochrane reviews can still be, at times, sub-standard [20, 54].

### Deficiencies in reporting quality of systematic reviews using PRISMA guidelines

The results from the PRISMA assessment indicated that reporting quality varied across systematic reviews. Specifically, just over half of the included systematic reviews reported at least 50% of the PRISMA items, while the rest reported less than half of the items. Despite many journals now requiring adherence to the PRISMA guidelines, there are still significant weaknesses in reporting quality in this field. In our analysis, the reporting scores on specific items varied widely, ranging from 15 to 100% reporting. In particular, reporting was lacking in the Methods section, highlighting the need for better and more transparent reporting of systematic review methodology.

### Limitations

This systematic quality review was strengthened by the use of two well-established and widely used tools for assessing methodological and reporting quality. However, there are limitations to this systematic quality review. First, the search results were limited to systematic reviews published in English, with any non-English reviews excluded during the screening process. We also did not consider specific requirements and guidelines of the journals, which may have skewed the results. Additionally, although we considered the quality of the systematic reviews, we did not consider the quality of the included studies within the systematic reviews or the results of the included systematic reviews. Finally, the e-cigarette field is relatively new, and we anticipate that the number of available reviews will continue to grow significantly. It is unclear whether reporting has improved over time, as we identified too few reviews to effectively assess this.

### Implications for policy makers, healthcare providers, and researchers

In a recent systematic review in *JAMA Pediatrics* investigating youth and young adult use of pod-based e-cigarettes, we identified numerous methodological deficiencies—according to AMSTAR-2 and PRISMA guidelines—including no defined key question, no predefined outcomes, a search strategy that was neither comprehensive nor reproducible, and a lack of risk of bias assessment [55]. Overall, these and numerous other methodological flaws only underscore the non-generalizability of this review’s findings. Thus, increased journal requirements that hold systematic reviews to comprehensive, consistent, and transparent standards (such as those exhibited in AMSTAR-2 and PRISMA) are fundamental to improving systematic review methodological and reporting quality.

Ultimately, the lack of high-quality systematic reviews can have significant negative implications for a number of stakeholders. For example, the lack of a comprehensive search strategy may result in the authors missing highly relevant studies. Furthermore, it is critical that, when conducting a systematic review, a protocol is developed a priori and made readily-accessible [56]; failure to do so may ultimately result in the introduction of bias into the review and may call into question both the transparency and reproducibility of the review. Specifically, a comparison of the protocol to the published systematic review is necessary to identify and prevent selective reporting. The lack of a clearly defined research question may also ultimately cause the review to become influenced by the literature identified by the search, rather than remaining focused on the original research question.

Similarly, providing a list of excluded studies, with justifications for exclusion, is required to reduce the risk of bias and ensure transparency regarding the impact of their exclusion from the review. Finally, an appropriate assessment of bias is critical to the interpretation of findings from systematic reviews and/or meta-analysis. Without an adequate assessment of bias, the outcomes of the systematic reviews may be skewed. As systematic reviews are expected to reflect the most comprehensive and highest level of evidence in health care, poor quality review methodology and reporting can misrepresent the results, thereby decreasing the utility of the reviews and/or providing misleading evidence for policy makers, healthcare providers, and researchers.

### Comparison of findings to other research fields

The main findings of this systematic quality review suggest that the overall methodological quality of systematic reviews investigating e-cigarette use and cigarette smoking behaviors was limited, with 18 of 20 systematic reviews receiving a score of “critically low” in the overall confidence of the results and 2 of 20 reviews receiving a score of “low”, and that the overall quality of reporting was moderate. The lack of high-quality systematic reviews is not unique to the tobacco research field.

A number of systematic reviews have investigated systematic review quality in other fields and have similarly determined the available reviews to be of generally lower quality. For example, a systematic quality review investigating the methodological and reporting quality of systematic reviews on tuberculosis concluded that although the reporting of systematic reviews was of moderate quality, the methodological quality was moderate to low [57]. Similarly, the reporting and methodological quality of systematic reviews of nursing interventions in patients with Alzheimer’s disease was found to be inadequate [58]. Another systematic quality review determined that with the publication of the PRISMA guidance in 2009, reporting quality had improved in the field of vascular surgery by 2012 (65% to 73%, *p* < 0.01) [59]. This highlights the necessity for standardized evaluation of reporting and methodological quality in systematic review, and improved rigor in journal requirements for their publication.

## Conclusions

Given the relevance of systematic reviews for informing regulatory decisions, health professionals on best practice, and research priorities, it is critical that the methodological quality of systematic reviews improve significantly. Although it is promising that many journals are now requiring compliance with the PRISMA guidelines for publication, future systematic reviews and meta-analyses in this field should strive to adhere to AMSTAR 2 and PRISMA guidelines to provide high quality analyses of the available data with transparent and complete reporting.

In conclusion, higher quality systematic reviews are required to determine whether there is an association between e-cigarette use and cigarette smoking behaviors. Based on findings from this systematic quality review, the available systematic reviews lack sufficient methodological rigor to support robust determinations regarding e-cigarette use and cigarette smoking behaviors.

## Supplementary Information

**Additional file 1**. **Appendix 1:** Search strategy. **Appendix 2:** List of articles reviewed in full text. **Appendix 3:** Full-text articles excluded (with reasons for exclusion). **Appendix 4:** A list of the included studies. **Appendix 5:** Characteristics of systematic reviews on e-cigarette use and combustible cigarette smoking initiation or cessation. **Appendix 6:** Characteristics of included studies. **Appendix 7:** Methodology and included studies in the included systematic reviews. **Appendix 8:** Risk of bias, statistical analysis, and heterogeneity methodology of included systematic reviews. **Appendix 9:** Scoring results of methodological quality using the AMSTAR 2 tool. **Appendix 10:** Summary of methodological quality using the AMSTAR 2 tool. **Appendix 11:** Scoring results of quality of reporting using the PRISMA tool. **Appendix 12:** Summary of quality of reporting using the PRISMA tool.

## Data Availability

All data and materials considered in this review are publicly available.
