# Supplementary material for: Reporting and methodological quality of systematic literature reviews evaluating the associations between e-cigarette use and cigarette smoking behaviors: a systematic quality review
Source: Harm Reduct J. 2021 Nov 27;18:121. doi: 10.1186/s12954-021-00570-9 (PMC8627036; doi:10.1186/s12954-021-00570-9)
Supplement: Supplementary file 1 — Additional file 1. Appendix 1: Search strategy. Appendix 2: List of articles reviewed in full text. Appendix 3: Full-text articles excluded (with reasons for exclusion). Appendix 4: A list of the included studies. Appendix 5: Characteristics of systematic reviews on e-cigarette use and combustible cigarette smoking initiation or cessation. Appendix 6: Characteristics of included studies. Appendix 7: Methodology and included studies in the included systematic reviews. Appendix 8: Risk of bias, statistical analysis, and heterogeneity methodology of included systematic reviews. Appendix 9: Scoring results of methodological quality using the AMSTAR 2 tool. Appendix 10: Summary of methodological quality using the AMSTAR 2 tool. Appendix 11: Scoring results of quality of reporting using the PRISMA tool. Appendix 12: Summary of quality of reporting using the PRISMA tool. [file 12954_2021_570_MOESM1_ESM.docx]

**APPENDIX 1: Search strategy**

The literature search was conducted by an Information Specialist. Search terms were developed using medical subject headings (MeSH) and text words related to the associations between e-cigarette use and combustible cigarette smoking initiation or cessation. The search strategy included using synonyms of search terms, truncation, wild card symbols, Boolean logic, proximity operators, and limits, in order to focus the search toward the most relevant clinical literature.

**INITIAL DATABASE SEARCH – JANUARY 01 2007 TO SEPTEMBER 18, 2019**

Database(s): Embase 1974 to 2019 September 19, Ovid MEDLINE(R) ALL 1946 to September 18, 2019, PsycINFO 1806 to September Week 2 2019

Search Strategy:

| **#** | **Searches** | **Results** |
| --- | --- | --- |
| 1 | (ecig$ or e-cig$ or e-voke$ or electronic nicotine or electronic cigarette$ or vape$ or vaping or vapourized nicotine or vaporized nicotine or vaporised nicotine).ti,ab. | 11781 |
| 2 | (electronic adj3 cig$).ti,ab. | 6108 |
| 3 | vaping/ | 1483 |
| 4 | electronic cigarettes/ | 8373 |
| 5 | electronic nicotine delivery systems/ | 7700 |
| 6 | or/1-2 | 11818 |
| 7 | or/3-5 | 9440 |
| 8 | or/6-7 | 12811 |
| 9 | tobacco/ | 73989 |
| 10 | nicotine/ | 79279 |
| 11 | (smok$ or tobacco$ or nicotin$ or cigar$ or cigs).ti,ab. | 963099 |
| 12 | or/9-11 | 987851 |
| 13 | 12 and 8 | 12234 |
| 14 | (systematic review or meta-analysis).pt. | 171035 |
| 15 | meta-analysis/ or systematic review/ or systematic reviews as topic/ or meta-analysis as topic/ or "meta analysis (topic)"/ or "systematic review (topic)"/ or exp technology assessment, biomedical/ or network meta-analysis/ | 572318 |
| 16 | ((systematic* adj3 (review* or overview*)) or (methodologic* adj3 (review* or overview*))).ti,ab,kf,kw. | 413610 |
| 17 | ((quantitative adj3 (review* or overview* or synthes*)) or (research adj3 (integrati* or overview*))).ti,ab,kf,kw. | 31117 |
| 18 | ((integrative adj3 (review* or overview*)) or (collaborative adj3 (review* or overview*)) or (pool* adj3 analy*)).ti,ab,kf,kw. | 63683 |
| 19 | (data synthes* or data extraction* or data abstraction*).ti,ab,kf,kw. | 57248 |
| 20 | (met analy* or metanaly* or technology assessment* or HTA or HTAs or technology overview* or technology appraisal*).ti,ab,kf,kw. | 23473 |
| 21 | (meta regression* or metaregression*).ti,ab,kf,kw. | 19250 |
| 22 | (meta-analy* or metaanaly* or systematic review* or biomedical technology assessment* or bio-medical technology assessment*).mp,hw. | 778189 |
| 23 | (cochrane or (health adj2 technology assessment) or evidence report).jw. | 44004 |
| 24 | (comparative adj3 (efficacy or effectiveness)).ti,ab,kf,kw. | 32898 |
| 25 | (outcomes research or relative effectiveness).ti,ab,kf,kw. | 25035 |
| 26 | (meta-analysis or systematic review).md. | 39770 |
| 27 | umbrella review*.ti,ab,kf,kw. | 682 |
| 28 | (evidence adj2 synthesis).ti,ab,kw,kf. | 11158 |
| 29 | or/14-28 | 971834 |
| 30 | 13 and 29 | 391 |
| 31 | limit 30 to english language | 382 |
| 32 | limit 31 to yr="2007 -Current" | 381 |
| 33 | remove duplicates from 32 | 244 |

**DATABASE SEARCH UPDATE– JANUARY 01 2019 TO JUNE 24, 2020**

Database(s): Embase 1974 to 2020 June 24, Ovid MEDLINE(R) ALL 1946 to June 23, 2020, APA PsycInfo 1806 to June Week 4 2020

Search Strategy:

| **#** | **Searches** | **Results** |
| --- | --- | --- |
| 1 | (ecig$ or e-cig$ or e-voke$ or electronic nicotine or electronic cigarette$ or vape$ or vaping or vapourized nicotine or vaporized nicotine or vaporised nicotine).ti,ab. | 15122 |
| 2 | (electronic adj3 cig$).ti,ab. | 7431 |
| 3 | vaping/ | 2786 |
| 4 | electronic cigarettes/ | 10510 |
| 5 | electronic nicotine delivery systems/ | 9517 |
| 6 | or/1-2 | 15164 |
| 7 | or/3-5 | 12187 |
| 8 | or/6-7 | 16414 |
| 9 | tobacco/ | 76186 |
| 10 | nicotine/ | 81589 |
| 11 | (smok$ or tobacco$ or nicotin$ or cigar$ or cigs).ti,ab. | 1007644 |
| 12 | or/9-11 | 1032983 |
| 13 | 12 and 8 | 15507 |
| 14 | (systematic review or meta-analysis).pt. | 192212 |
| 15 | meta-analysis/ or systematic review/ or systematic reviews as topic/ or meta-analysis as topic/ or "meta analysis (topic)"/ or "systematic review (topic)"/ or exp technology assessment, biomedical/ or network meta-analysis/ | 631900 |
| 16 | ((systematic* adj3 (review* or overview*)) or (methodologic* adj3 (review* or overview*))).ti,ab,kf,kw. | 467352 |
| 17 | ((quantitative adj3 (review* or overview* or synthes*)) or (research adj3 (integrati* or overview*))).ti,ab,kf,kw. | 33217 |
| 18 | ((integrative adj3 (review* or overview*)) or (collaborative adj3 (review* or overview*)) or (pool* adj3 analy*)).ti,ab,kf,kw. | 69903 |
| 19 | (data synthes* or data extraction* or data abstraction*).ti,ab,kf,kw. | 62997 |
| 20 | (met analy* or metanaly* or technology assessment* or HTA or HTAs or technology overview* or technology appraisal*).ti,ab,kf,kw. | 25394 |
| 21 | (meta regression* or metaregression*).ti,ab,kf,kw. | 21742 |
| 22 | (meta-analy* or metaanaly* or systematic review* or biomedical technology assessment* or bio-medical technology assessment*).mp,hw. | 857636 |
| 23 | (cochrane or (health adj2 technology assessment) or evidence report).jw. | 46246 |
| 24 | (comparative adj3 (efficacy or effectiveness)).ti,ab,kf,kw. | 35203 |
| 25 | (outcomes research or relative effectiveness).ti,ab,kf,kw. | 26409 |
| 26 | (meta-analysis or systematic review).md. | 43982 |
| 27 | umbrella review*.ti,ab,kf,kw. | 969 |
| 28 | (evidence adj2 synthesis).ti,ab,kw,kf. | 12480 |
| 29 | or/14-28 | 1062488 |
| 30 | 13 and 29 | 536 |
| 31 | limit 30 to english language | 523 |
| 32 | limit 31 to yr="2019 -Current" | 204 |
| 33 | remove duplicates from 32 | 132 |

**APPENDIX 2: List of articles reviewed in full text**

| **Record #** | **Study Information** |
| --- | --- |
| 1 | UndernerM,​ PerriotJ,​ BrousseG,​ et al (2019) Stopping and reducing smoking in patients with schizophrenia. Encephale; 45.  Keywords: adult,​article,​behavior therapy,​cognitive therapy,​controlled study,​craving,​drug combination,​drug therapy,​electronic cigarette,​female,​human,​male,​Medline,​*nicotine replacement therapy,​physical activity,​*psychosis,​randomized controlled trial (topic),​reinforcement,​smoking cessation,​*smoking cessation program,​social competence,​systematic review,​tobacco consumption,​tobacco dependence,​validation process,​*amfebutamone,​atypical antipsychotic agent,​placebo,​*varenicline  Abstract: Objectives: This systematic review of the literature looked at data on pharmacological and non-pharmacological strategies of smoking cessation and reduction of consumption in patients with schizophrenia. Method(s): The research was conducted on Medline for the period 1980-2018. We included randomized controlled trials,​ including preliminary studies of stable schizophrenic patients with no other severe psychiatric disorder and no other substance use than tobacco,​ treated with antipsychotic medications. Individual or group smoking cessation programs with or without pharmacological treatment,​ including a validation of abstinence,​ were included. Result(s): Pharmacotherapies for nicotine dependence-nicotine replacement therapy (n = 3),​ bupropion (n = 6),​ varenicline (n = 8),​ association of medications (n = 4)-were used in 23 studies combined with behavioral support. Compared to the placebo,​ bupropion and varenicline at the end of treatment were found to be the most effective pharmacotherapies to stop or reduce smoking and control craving. All the medications were well tolerated and did not lead to aggravation of psychosis or changes in symptoms. Non-pharmacological interventions: behavioral and cognitive therapies (n = 5) combined with pharmacological treatment facilitated the management of smoking risk situations and improved adherence to antipsychotics; other psychosocial interventions (n = 7) allowed the development of social skills; contigency management strategies with financial reinforcement can be used (n = 4); the practice of physical activity and the use of an electronic cigarette allowed reduction of tobacco consumption. The results of transcranial electromagnetic stimulation studies (n = 6) were discordant. Atypical antipsychotics appear to be associated with a better success of attempts to stop smoking. Conclusion(s): Smoking cessation strategies for patients with schizophrenia appear to be effective and should combine (1) smoking cessation medications with sufficient duration,​ (2) diversified psychosocial approaches and (3) physical activity practice.Copyright © 2019 L'Encephale,​ Paris |
| 12 | Signes-CostaJ,​ deGranda-OriveJI,​ RamosPinedoA,​ et al (2019) Official Statement of the Spanish Society of Pulmonology and Thoracic Surgery (SEPAR) on Electronic Cigarettes and IQOS. Archivos de Bronconeumologia; .  Keywords: adolescent,​article,​burn,​clinical trial (topic),​controlled study,​counseling,​*electronic cigarette,​human,​meta analysis,​nicotine replacement therapy,​*pulmonology,​*smoking cessation,​surgery,​*thorax surgery,​amfebutamone,​varenicline  Abstract: The use of novel tobacco products,​ particularly the electronic cigarette (EC) and partial tobacco combustion devices (HnB systems: Heat not Burn),​ has increased exponentially,​ particularly among adolescents and young people. The health authorities and scientific societies have shown concern about issues surrounding safety and effectiveness (as a method of smoking cessation). A study of the available scientific evidence has concluded that the safety of the vapor or fumes inhaled by the users of these devices cannot be guaranteed. Contradictory results from various clinical trials and meta-analyses also mean that these devices cannot be recommended for their effectiveness in cessation,​ especially when safe and effective treatments are available to help quit smoking (varenicline,​ nicotine replacement therapy,​ and bupropion,​ combined with psychological counseling).Copyright © 2019 SEPAR |
| 13 | FadusMC,​ SmithTT,​ SquegliaLM (2019) The rise of e-cigarettes,​ pod mod devices,​ and JUUL among youth: Factors influencing use,​ health implications,​ and downstream effects. Drug and Alcohol Dependence; 201.  Keywords: adolescent,​adult,​advertising,​attitude to health,​awareness,​cannabis use,​chemical composition,​flavor,​health hazard,​human,​peer pressure,​prevalence,​priority journal,​review,​smoking ban,​smoking cessation,​smoking habit,​smoking regulation,​social aspect,​social media,​systematic review,​United States,​*vaping,​young adult,​cannabis,​*electronic cigarette  Abstract: Background: Electronic cigarettes (e-cigarettes) were first introduced in the U.S. market in 2006,​ with the more recent evolution of "pod-mod" e-cigarettes such as JUUL introduced in 2015. Although marketed as a smoking cessation tool,​ e-cigarettes are rarely used for this purpose in youth. This review aims to synthesize the literature regarding e-cigarette use among youth,​ and provides a resource for clinicians,​ educators,​ and families that helps answer commonly asked questions about e-cigarettes. Method(s): PubMed,​ Scopus,​ and PsycINFO search was performed using search terms "Electronic Nicotine Delivery Systems,​" "e cigarettes,​" "e-cigarettes,​" "electronic cigarettes,​" "vaping,​" "JUUL,​" "e-cigs,​" and "vape pens." Search results were filtered to only include those related to adolescents and young adults. Result(s): E-cigarette use among youth is common,​ with rates of use increasing from 1.5% in 2011 to 20.8% in 2018. Pod mod devices such as JUUL have gained favor among youth for their sleek design,​ user-friendly function,​ desirable flavors,​ and ability to be used discreetly in places where smoking is forbidden. Adolescents are often uninformed about the constituents of e-cigarettes,​ and little is known about the long-term effects of e-cigarettes. Studies have suggested a "gateway" effect for combustible cigarettes and cannabis use. Conclusion(s): E-cigarette use is becoming increasingly common among youth,​ leading to a myriad of questions and concerns from providers,​ educators,​ and family members. More research is needed to determine the ultimate public health impact of e-cigarette use. The authors provide a summary table of frequently asked questions in order to help clarify these common concerns.Copyright © 2019 |
| 14 | HeissC (2019) Electronic cigarettes: Replacing one evil with another?. European Journal of Preventive Cardiology; 26.  Keywords: arterial stiffness,​atrial fibrillation,​blood pressure,​cardiovascular disease,​cardiovascular risk,​editorial,​endothelial dysfunction,​heart rate,​human,​hypertension,​knowledge,​nicotine replacement therapy,​prevalence,​priority journal,​risk factor,​*smoking,​*smoking cessation,​social support,​systematic review,​tobacco use,​alkaloid,​heavy metal,​reactive oxygen metabolite,​*electronic cigarette  Abstract: |
| 15 | WallaceAM,​ ForonjyRE (2019) Electronic cigarettes: Not evidence-based cessation. Translational Lung Cancer Research; 8.  Keywords: artificial intelligence,​behavior change,​cigarette smoking,​*evidence based practice,​ex smoker,​food and drug administration,​health survey,​human,​meta analysis (topic),​note,​Ontario,​pharmacist,​randomized controlled trial (topic),​smokeless tobacco,​*smoking cessation,​systematic review (topic),​three dimensional printing,​tobacco dependence,​United Kingdom,​*electronic cigarette,​e-cigarettes  Abstract: |
| 16 | VillantiAC,​ FeirmanSP,​ NiauraRS,​ et al (2018) How do we determine the impact of e-cigarettes on cigarette smoking cessation or reduction? Review and recommendations for answering the research question with scientific rigor. Addiction (Abingdon,​ England); 113.  Keywords: adult,​article,​Australia,​*cigarette smoking,​controlled study,​*electronic cigarette,​Europe,​female,​human,​Iran,​Korea,​male,​Medline,​New Zealand,​observational study,​outcome assessment,​prospective study,​randomized controlled trial (topic),​*rigor,​*smoking cessation,​systematic review,​United States,​*nicotine  Abstract: AIMS: To propose a hierarchy of methodological criteria to consider when determining whether a study provides sufficient information to answer the question of whether e-cigarettes can facilitate cigarette smoking cessation or reduction. DESIGN: A PubMed search to 1 February 2017 was conducted of all studies related to e-cigarettes and smoking cessation or reduction. SETTINGS: Australia,​ Europe,​ Iran,​ Korea,​ New Zealand and the United States. PARTICIPANTS AND STUDIES: 91 articles. MEASUREMENTS: Coders organized studies according to six proposed methodological criteria: (1) examines outcome of interest (cigarette abstinence or reduction),​ (2) assesses e-cigarette use for cessation as exposure of interest,​ (3) employs appropriate control/comparison groups,​ (4) ensures that measurement of exposure precedes the outcome,​ (5) evaluates dose and duration of the exposure and (6) evaluates the type and quality of the e-cigarette used. FINDINGS: Twenty-four papers did not examine the outcomes of interest. Forty did not assess the specific reason for e-cigarette use as an exposure of interest. Twenty papers did not employ prospective study designs with appropriate comparison groups. The few observational studies meeting some of the criteria (duration,​ type,​ use for cessation) triangulated with findings from three randomized trials to suggest that e-cigarettes can help adult smokers quit or reduce cigarette smoking. CONCLUSION(S): Only a small proportion of studies seeking to address the effect of e-cigarettes on smoking cessation or reduction meet a set of proposed quality standards. Those that do are consistent with randomized controlled trial evidence in suggesting that e-cigarettes can help with smoking cessation or reduction.Copyright © 2017 Society for the Study of Addiction. |
| 21 | KrusemannE,​ BoesveldtS,​ DeGraafK,​ et al (2018) An overview of the role of flavors in e-cigarette addiction. Tobacco Induced Diseases; 16.  Keywords: *addiction,​adult,​candy,​*electronic cigarette,​Embase,​female,​*flavor,​fruit,​human,​juvenile,​learning,​male,​Medline,​nonhuman,​nonsmoker,​practice guideline,​scientist,​smoking,​systematic review,​Vanilla,​conference abstract  Abstract: Background: E-cigarettes are available in a wide variety of flavors,​ which increases sensory appeal and stimulates smoking initiation,​ especially among youth. To determine regulatory measures on flavors in e-cigarettes,​ e.g. restriction or prohibition,​ more insight should be obtained in the role of flavors in e-cigarette addiction. Core components of addiction are liking,​ learning,​ and wanting. We provide an overview of e-cigarette flavors related to these aspects of addiction,​ including differences between youth and adults,​ and smokers and non-smokers. In addition,​ we aim to identify e-liquid flavors that are representative for different flavor categories (e.g. sweet,​ fruit,​ tobacco). Method(s): A systematic literature review was performed in May 2017 using PubMed and EMBASE databases. Key words included terms to capture concepts associated with e-cigarettes,​ flavors,​ liking,​ learning,​ and wanting in articles published from database inception to the search date. Results were independently screened (0.92 Cohen's Kappa) and reviewed. Result(s): Searches yielded 387 unique studies of which 32 were included. Research designs varied between cross-sectional,​ experimental,​ mixed-method,​ case study,​ and longitudinal. Flavors were described as an important reason for e-cigarette initiation. Youth mainly prefer fruit and sweet flavors,​ while tobacco is more popular among adults. Studies used different flavor categories such as sweet,​ fruit,​ tobacco,​ mint,​ candy and dessert. E-liquids representing these categories varied across study designs (e.g.,​ vanilla represented the sweet,​ candy,​ as well as the dessert category). Nevertheless,​ results of different studies were comparable for each flavor category. Conclusion(s): Published research mainly focused on flavors for e-cigarette liking. Research gaps exist on the learning and wanting components of addiction. Our review helps researchers developing study designs to investigate e-cigarette addiction. In addition,​ our overview will provide policy makers a first guideline towards regulating the amount of e-cigarette flavors available in order to prevent e-cigarette initiation among non-smoking youth. |
| 32 | LivingstonCJ,​ FreemanRJ,​ CostalesVC,​ et al (2019) Electronic Nicotine Delivery Systems or E-cigarettes: American College of Preventive Medicine's Practice Statement. American Journal of Preventive Medicine; 56.  Keywords: article,​consumer,​follow up,​health care organization,​health care policy,​human,​incidence,​*passive smoking,​population health,​population research,​preventive medicine,​prospective study,​risk assessment,​smoking cessation,​systematic review,​vaping,​*electronic cigarette  Abstract: Introduction: E-cigarettes or or electronic nicotine delivery systems (ENDS) have rapidly gained popularity in the U.S. Controversy exists about the safety and efficacy of ENDS. The American College of Preventive Medicine's Prevention Practice Committee undertook a consensus-based evidence review process to develop a practice statement for the American College of Preventive Medicine. Method(s): A rapid review of the literature was performed through June 2017 to identify efficacy,​ patient-oriented harms,​ and the impact on population health. Result(s): On an individual level,​ limited evidence suggests that ENDS may be effective at reducing cigarette use among adult smokers intending to quit. There is insufficient evidence addressing potential long-term harms of ENDS,​ and limited evidence is available about short-term harms of ENDS and the impact of secondhand exposure. Although ENDS appear safer than combustible cigarettes,​ they are not without risk. Among youth there is no known benefit and significant concern for harm. On a population level,​ there may be significant harms associated with ENDS,​ particularly among youth nonsmokers. The long-term balance of potential benefits versus harms from the individual and population perspectives are unclear. Conclusion(s): The American College of Preventive Medicine developed practice recommendations that include encouraging screening for ENDS use,​ strategies to prevent the initiation of ENDS use in nonsmokers,​ particularly in youth,​ adoption of a harm reduction model for smokers intending to quit in those who refuse or fail to quit with evidence-based smoking-cessation methods,​ recommendations on policy and regulatory strategies to decrease public use of ENDS and regulation of their components,​ and future research needs.Copyright © 2019 Elsevier Ltd |
| 33 | AlbanoC,​ YangF,​ BuckleyB,​ et al (2016) A systematic review on the health and safety of electronic cigarettes. Journal of Managed Care and Specialty Pharmacy; 22.  Keywords: adverse event,​advertising,​drug delivery device,​drug efficacy,​drug safety,​health hazard,​human,​Medline,​pharmacist,​pharmacy,​public health,​smoking cessation,​systematic review,​*vaping,​vapor,​heavy metal,​conference abstract  Abstract: BACKGROUND: The growth in sales and use of electronic cigarettes has skyrocketed in the past decade. With limited research and conclusions on its health,​ safety,​ and efficacy,​ we undertook a systematic review of published studies of electronic cigarettes. OBJECTIVE(S): To summarize the available scientific research concerning electronic cigarettes with a focus on public health and safety of electronic cigarette usage; in addition,​ its implications in pharmacy practice will be elucidated. METHOD(S): A systematic review and analysis of articles in PubMed was completed. Inclusion criteria of articles about electronic cigarettes and health included search for all articles containing "electronic cigarettes",​ "e-cigs",​ or "vaping" in the title,​ abstract,​ or body. Articles published in non-European or non-United States of America countries were excluded. RESULT(S): We identified 85 articles about electronic cigarettes. These articles and their conclusions were then divided into four general categories about electronic cigarette chemical profiles,​ use in smoking cessation,​ health effects,​ and usage. From these conclusions we identified certain themes pertaining to the health and safety of electronic cigarette usage. The health of electronic cigarette usage is defined by its use in smoking cessation and health risks. We found evidence for the usage of electronic cigarettes in smoking cessation with minor short-term adverse effect. The safety of electronic cigarette usage is defined by the composition of electronic cigarettes,​ the e-liquid,​ and vapor. We found discrepancies between e-liquid labeling and contents and the presence of heavy metals in electronic cigarette vapor among other chemicals. CONCLUSION(S): Electronic cigarettes are drug delivery devices now regulated by the FDA. The presence of electronic cigarette usage is a growing tread in America especially in younger populations. New FDA regulations limits marking for therapeutic purposes but previous advertisements about the benefits of electronic cigarette has permeated the general populace. As pharmacist and public health advocates,​ it is important to clarify the information surrounding electronic cigarettes and make recommendation on their use. |
| 36 | MayelM (2018) Are vapes an effective device for smoking cessation or a gateway to conventional tobacco smoking?. Canadian Journal of Respiratory Therapy; 54.  Keywords: adult,​craving,​drug withdrawal,​electronic cigarette,​female,​human,​male,​Medline,​randomized controlled trial (topic),​*smoking cessation,​smoking reduction,​systematic review,​withdrawal syndrome,​young adult,​cotinine,​nicotine patch,​conference abstract  Abstract: BACKGROUND: With tobacco smoking still being a leading cause of deaths worldwide,​ smoking cessation has become an area of great focus in the recent years. Vapes or e-cigarettes have been getting increasing attention in its effectiveness as a smoking cessation device as it the only device that provides the same illusion and behavioral resemblance to a traditional cigarette. OBJECTIVE(S): To investigate if vapes or e-cigarettes aid in smoking cessation or promote smoking. METHOD(S): A literature search was completed in November 2017 using the following databases: PubMed and Ovid. Randomized controlled trials were only selected to be included in the search. After all inclusion and exclusion criteria were applied,​ four studies were chosen. Reference lists of selected studies were also reviewed for relevant studies. RESULT(S): All included studies were randomized controlled trials with adult participants over the age of 18 years who were current smoker. The intervention used in the studies included e-cigarettes with a nicotine level between 0 and 18 mg and traditional tobacco cigarettes or nicotine patch as a comparison. DISCUSSION: Studies included in this review measured the effect of e-cigarettes on cravings and withdrawal symptoms,​ smoking reduction or abstinence,​ and eCO and cotinine levels. Most studies showed evidence that e-cigarette use leads to an overall reduction in tobacco smoking. CONCLUSION(S): Although the majority of the studies included in this review revealed that e-cigarettes result in a significant decrease in smoking,​ further research needs to be conducted to prove its effectiveness for complete smoking abstinence,​ as well as the harms and risks associated with its use. |
| 40 | HameeRH (2018) Human health effects of electronic cigarettes: A review. Indian Journal of Public Health Research and Development; 9.  Keywords: article,​chemical composition,​clinical research,​creativity,​health hazard,​*health impact assessment,​human,​public health message,​risk assessment,​smoking,​smoking cessation,​systematic review,​tobacco use,​vapor,​nicotine,​*electronic cigarette  Abstract: In the modern generation of the 21st century,​ there has been increased and rapid use of electronic cigarettes in most countries across the globe. These electronic cigarettes are marketed and perceived to be the healthy alternative and life-choice consumables as compared to the conventional cigarettes. As an implication,​ electronic cigarettes have gained popularity amongst teenagers and never-smokers thus becoming the current issue on the public health. However,​ the data available in the online databases is limited regarding the safety issues and the efficacy of treatment,​ reduction of tobacco dependency to the public. Most of the people do not know and recognize the overall impacts of these devices which deliver nicotine-containing vapor on demographic health. This study and systematic review acknowledges the clinical and laboratory research that are mainstays of health impacts of E-cigarettes use versus the devastating health risks of smoking conventional or tobacco cigarettes. Presently,​ the data available reveals that e-cigs are the less harmful,​ and,​ thus are best alternatives to traditional smoking. Advancements,​ creativity,​ research and innovation on the electronic cigarettes will help in community engagement and enhance public awareness. This will be effective smoking as it reduces residual and adverse effects by revisiting quality control standards.Copyright © 2018,​ Indian Journal of Public Health Research and Development. All rights reserved. |
| 41 | KaurG,​ PinkstonR,​ McLemoreB,​ et al (2018) Immunological and toxicological risk assessment of e-cigarettes. European Respiratory Review; 27.  Keywords: aerosol,​air quality,​asthma,​chemical analysis,​chemical composition,​chronic obstructive lung disease,​cytokine production,​device safety,​*electronic cigarette,​exposure,​health hazard,​human,​immunoregulation,​*immunotoxicity,​indoor air pollution,​inflammation,​innate immunity,​nonhuman,​passive smoking,​protein analysis,​review,​signal transduction,​smoking cessation,​smoking regulation,​systematic review,​*toxicity testing,​*vaping,​cotinine,​glycerol,​immunoglobulin enhancer binding protein/ec [Endogenous Compound],​Janus kinase/ec [Endogenous Compound],​mitogen activated protein kinase/ec [Endogenous Compound],​nicotine,​propylene glycol,​STAT protein/ec [Endogenous Compound],​tobacco smoke  Abstract: Knowledge of the long-term toxicological and immunological effects of e-cigarette (e-cig) aerosols remains elusive due to the relatively short existence of vaping. Therefore,​ we performed a systematic search of articles published in public databases and analyzed the research evidence in order to provide critical information regarding e-cig safety. Electronic nicotine delivery systems (or e-cigs) are an alternative to traditional cigarettes for the delivery of nicotine and are typically filled with glycerol or propylene glycol-based solutions known as e-liquids. Though present in lower quantities,​ e-cig aerosols are known to contain many of the harmful chemicals found in tobacco smoke. However,​ due to the paucity of experimental data and contradictory evidence,​ it is difficult to draw conclusive outcomes regarding toxicological,​ immunological and clinical impacts of e-cig aerosols. Excessive vaping has been reported to induce inflammatory responses including mitogen-activated protein kinase,​ Janus tyrosine kinase/signal transducer and activator of transcription and nuclear factor-kappaB signalling,​ similar to that induced by tobacco smoke. Based on recent evidence,​ prolonged exposure to some constituents of e-cig aerosols might result in respiratory complications such as asthma,​ chronic obstructive pulmonary disease and inflammation. Future studies are warranted that focus on establishing correlations between e-cig types,​ generations and e-liquid flavours and immunological and toxicological profiles to broaden our understanding about the effects of vaping.Copyright © ERS 2018. |
| 42 | GhamriRA (2018) Identification of the most effective pharmaceutical products for smoking cessation: A literature review. Journal of Substance Use; 23.  Keywords: alcoholism,​chronic obstructive lung disease,​drug dependence,​electronic cigarette,​high risk population,​human,​Human immunodeficiency virus infection,​mental patient,​meta analysis (topic),​nicotine replacement therapy,​pregnancy,​pregnant woman,​priority journal,​randomized controlled trial (topic),​review,​*smoking cessation,​systematic review (topic),​systematic review,​tobacco,​*tobacco dependence/dt [Drug Therapy],​amfebutamone/dt [Drug Therapy],​clonidine/dt [Drug Therapy],​cytisine/dt [Drug Therapy],​fluoxetine/dt [Drug Therapy],​mecamylamine/dt [Drug Therapy],​moclobemide/dt [Drug Therapy],​nicotine gum/dt [Drug Therapy],​nicotine patch/dt [Drug Therapy],​nortriptyline/dt [Drug Therapy],​rimonabant/dt [Drug Therapy],​selegiline/dt [Drug Therapy],​taranabant/dt [Drug Therapy],​varenicline/dt [Drug Therapy],​venlafaxine/dt [Drug Therapy],​heat not burn tobacco product  Abstract: Introduction: The major causes of excess mortality among smokers are diseases related to smoking,​ including cancer as well as respiratory and vascular diseases. People who stop smoking greatly reduce their risk of disease and early death,​ so effective smoking-cessation interventions are extremely important management strategies. Aim(s): To undertake a major literature review to identify the most effective pharmaceutical products for smoking cessation and detail the latest research in smoking-cessation interventions. Method(s): The Google Scholar,​ PubMed,​ Medline,​ and the Cochrane Library of Systematic Reviews databases were searched. The types of studies searched for were randomized controlled trials,​ systematic reviews,​ and meta-analyses. All studies were included except those involving patients with chronic obstructive pulmonary disease or infection by human immunodeficiency virus. Searched studies assessed nicotine replacement therapy (NRT),​ varenicline,​ combination therapy,​ bupropion,​ and antidepressants. Result(s): The literature search focused on the three first-line treatments for smoking cessation: NRT,​ bupropion,​ and varenicline. Current research in non-first-line treatments (electronic cigarettes,​ cytisine,​ "heat-not-burn" tobacco products) as well as smoking-cessation strategies in high-risk individuals (pregnant women,​ individuals with mental-health or addiction issues) was also detailed. Conclusion(s): Except for high-risk groups,​ use of NRT,​ bupropion,​ and varenicline can result in a higher prevalence of smoking cessation compared with placebos. Varenicline and combination NRT appear to be the most effective smoking-cessation strategies.Copyright © 2018,​ © 2018 Taylor & Francis Group,​ LLC. |
| 48 | ChatterjeeK,​ AlzghoulB,​ InnabiA,​ et al (2018) Is vaping a gateway to smoking: A review of the longitudinal studies. International Journal of Adolescent Medicine and Health; 30.  Keywords: adolescent,​female,​human,​incidence,​*longitudinal study,​male,​Medline,​*psychology,​review,​Scopus,​smoking cessation,​systematic review,​tobacco industry,​*vaping,​Web of Science,​young adult  Abstract: Background: The use of e-cigarettes (ECs) is rising globally. There is concern that e-cigarette may actually lead to smoking,​ especially amongst adolescents. Objective(s): To perform a comprehensive review of literature reporting the longitudinal effects of e-cigarette use on onset of smoking among adolescents and young adults. Method(s): A search was conducted using PubMed,​ Google Scholar,​ Scopus,​ and Web of Science in February 2016 to identify the studies containing data on EC use among adolescents and young adults (age < 30 years). We then narrowed our search to only include longitudinal studies with data on EC and conventional cigarette smoking among this population. Result(s): Four longitudinal studies were identified that analyzed the use of ECs and smoking at both baseline and follow-ups in the target population. These studies demonstrated that EC use is associated with an increase in combustible cigarette smoking,​ even amongst the adolescents who were not susceptible to smoking. Conclusion(s): This review highlights the strong evidence that not only are ECs are not an effective tool for smoking cessation among adolescents,​ they actually are associated with higher incidence of combustible cigarette smoking. Policy makers need to recognize of the insidious nature of this campaign by the tobacco industry and design policies to regulate it.Copyright © 2018 Walter de Gruyter GmbH,​ Berlin/Boston. |
| 51 | OnorIO,​ StirlingDL,​ WilliamsSR,​ et al (2017) Clinical effects of cigarette smoking: Epidemiologic impact and review of pharmacotherapy options. International Journal of Environmental Research and Public Health; 14.  Keywords: acupuncture,​aortic aneurysm,​aversion therapy,​awareness,​bioaccumulation,​chemical analysis,​chemical structure,​chronic obstructive lung disease,​cognitive behavioral therapy,​counseling,​DNA damage,​electronic cigarette,​ethnicity,​gene mutation,​health care cost,​health care policy,​health education,​human,​hypnosis,​incidence,​ischemic heart disease,​lung circulation,​national health service,​peripheral vascular disease,​review,​self help,​sex difference,​signal transduction,​*smoking,​smoking cessation,​systematic review,​Th2 cell,​*tobacco dependence/dt [Drug Therapy],​*tobacco dependence/th [Therapy],​tobacco dependence/dt [Drug Therapy],​United States,​amfebutamone/dt [Drug Therapy],​clonidine/dt [Drug Therapy],​nicotine,​nicotine gum,​nicotine lozenge/dt [Drug Therapy],​nicotine patch,​nortriptyline/dt [Drug Therapy],​varenicline/dt [Drug Therapy]  Abstract: Cigarette smoking-a crucial modifiable risk factor for organ system diseases and cancer-remains prevalent in the United States and globally. In this literature review,​ we aim to summarize the epidemiology of cigarette smoking and tobacco use in the United States,​ pharmacology of nicotine-the active constituent of tobacco,​ and health consequence of cigarette smoking. This article also reviews behavioral and pharmacologic interventions for cigarette smokers and provides cost estimates for approved pharmacologic interventions in the United States. A literature search was conducted on Google Scholar,​ EBSCOhost,​ ClinicalKey,​ and PubMed databases using the following headings in combination or separately: cigarette smoking,​ tobacco smoking,​ epidemiology in the United States,​ health consequences of cigarette smoking,​ pharmacologic therapy for cigarette smoking,​ and non-pharmacologic therapy for cigarette smoking. This review found that efficacious non-pharmacologic interventions and pharmacologic therapy are available for cessation of cigarette smoking. Given the availability of efficacious interventions for cigarette smoking cessation,​ concerted efforts should be made by healthcare providers and public health professionals to promote smoking cessation as a valuable approach for reducing non-smokers' exposure to environmental tobacco smoke.Copyright © 2017 by the authors. |
| 53 | SonejiS,​ PrimackBA,​ PierceJP,​ et al (2017) Re: Modeling the effects of e-cigarettes on smoking behavior: Implications for future adult smoking prevalence. Epidemiology; 28.  Keywords: adult,​*electronic cigarette,​human,​letter,​medical literature,​meta analysis (topic),​prevalence,​priority journal,​public health,​risk assessment,​*smoking  Abstract: |
| 56 | GageSH,​ MaynardOM (2017) Smoke-free policies in psychiatric hospitals need resources. The Lancet Psychiatry; 4.  Keywords: electronic cigarette,​human,​mental disease,​*mental hospital,​mortality,​note,​patient advocacy,​physical violence,​practice guideline,​priority journal,​*smoking ban,​smoking cessation,​staff training,​systematic review (topic),​tobacco dependence  Abstract: |
| 58 | Orellana-BarriosMA,​ PayneD,​ Medrano-JuarezRM,​ et al (2016) Electronic Cigarettes for Smoking Cessation. American Journal of the Medical Sciences; 352.  Keywords: drug cost,​*electronic cigarette,​harm reduction,​human,​nicotine replacement therapy,​review,​*smoking cessation,​systematic review,​tobacco dependence/dt [Drug Therapy],​tobacco dependence/th [Therapy],​amfebutamone/dt [Drug Therapy],​nicotine/dt [Drug Therapy],​nicotine gum/dt [Drug Therapy],​nicotine patch/dt [Drug Therapy],​placebo,​varenicline/dt [Drug Therapy]  Abstract: The use of electronic cigarettes (e-cigarettes) is increasing,​ but their use as a smoking-cessation aid is controversial. The reporting of e-cigarette studies on cessation is variable and inconsistent. To date,​ only 1 randomized clinical trial has included an arm with other cessation methods (nicotine patches). The cessation rates for available clinical trials are difficult to compare given differing follow-up periods and broad ranges (4% at 12 months with non-nicotine e-cigarettes to 68% at 4 weeks with concomitant nicotine e-cigarettes and other cessation methods). The average combined abstinence rate for included prospective studies was 29.1% (combination of 6-18 months rates). There are few comparable clinical trials and prospective studies related to e-cigarettes use for smoking cessation,​ despite an increasing number of citations. Larger randomized clinical trials are essential to determine whether e-cigarettes are effective smoking-cessation devices.Copyright © 2016 Southern Society for Clinical Investigation |
| 59 | Orellana-BarriosMA,​ PayneD,​ NugentK (2016) E-cigarettes and smoking cessation. The Lancet Respiratory Medicine; 4.  Keywords: device safety,​*electronic cigarette,​human,​intermethod comparison,​letter,​meta analysis (topic),​model,​online system,​priority journal,​self report,​smoking,​*smoking cessation,​substitution therapy,​telephone interview,​tobacco use,​nicotine patch  Abstract: |
| 60 | GreenhillR,​ DawkinsL,​ NotleyC,​ et al (2016) Adolescent Awareness and Use of Electronic Cigarettes: A Review of Emerging Trends and Findings. Journal of Adolescent Health; 59.  Keywords: adolescent,​*adolescent health,​*adolescent smoking,​*awareness,​Canada,​*electronic cigarette,​Finland,​Germany,​Greece,​human,​Ireland,​New Zealand,​priority journal,​review,​systematic review,​United Kingdom,​nicotine  Abstract: Adult electronic cigarette (e-cigarette) use is increasing globally,​ and early studies have suggested that similar trends may be observed among the adolescent population,​ albeit at lower levels. The current literature review presents data collected since 2014 from 21 cross-sectional studies and one cohort study that were all published in English. In particular,​ it focuses on awareness,​ ever use,​ past 30-day use,​ and regular use of e-cigarettes. The article suggests that adolescents are nearing complete awareness of e-cigarettes. Furthermore,​ in relation to ever use and past 30-day use,​ higher prevalence rates continue to be reported across time,​ especially in the United States. Nonetheless,​ reported regular use of e-cigarettes remains much lower than past 30-day use,​ although conclusions are limited due to inconsistencies with measurement and consequent lack of cross-cultural applicability. The majority of studies do not report whether adolescents use non-nicotine e-cigarettes. There is a current absence of longitudinal studies that explore any association between e-cigarettes and tobacco use and little qualitative data that may illuminate how and why adolescents use e-cigarettes. Through addressing these methodological limitations,​ future research will be able to inform health care and policy more effectively.Copyright © 2016 |
| 62 | CobbNK,​ SontiR (2016) E-cigarettes: The science behind the smoke and mirrors. Respiratory Care; 61.  Keywords: article,​aspiration pneumonia,​*electronic cigarette,​food and drug administration,​health practitioner,​heart rate,​human,​lung resistance,​market,​meta analysis (topic),​nicotine replacement therapy,​particulate matter,​randomized controlled trial (topic),​respiratory system,​smoking cessation,​vapor,​*cigarette smoke,​flavoring agent,​formaldehyde,​glycerol,​interleukin 6/ec [Endogenous Compound],​nicotine,​propylene glycol  Abstract: E-cigarettes are a diverse set of devices that are designed for pulmonary delivery of nicotine through an aerosol,​ usually consisting of propylene glycol,​ nicotine,​ and flavorings. The devices heat the nicotine solution using a battery-powered circuit and deliver the resulting vapor into the proximal airways and lung. Although the current devices on the market appear to be safer than smoking combusted tobacco,​ they have their own inherent risks,​ which remain poorly characterized due to widespread product variability. Despite rising use throughout the United States,​ predominantly by smokers,​ limited evidence exists for their efficacy in smoking cessation. Pending regulation by the FDA will enforce limited disclosures on the industry but will not directly impact safety or efficacy. Meanwhile,​ respiratory health practitioners will need to tailor their discussions with patients,​ taking into account the broad range of existing effective smoking cessation techniques,​ including pharmaceutical nicotine replacement therapy.Copyright © 2016 Daedalus Enterprises. |
| 64 | TuanRS (2016) Prenatal substance use and developmental disorders: Overview and highlights. Birth Defects Research Part C - Embryo Today: Reviews; 108.  Keywords: behavior disorder,​brain function,​cognitive defect,​congenital malformation,​*developmental disorder,​DNA damage,​editorial,​electronic cigarette,​fetal alcohol syndrome,​fetus development,​human,​maternal smoking,​nerve cell plasticity,​neurotoxicity,​neurotransmission,​pregnant woman,​*prenatal exposure,​priority journal,​*substance use,​systematic review (topic),​alcohol/to [Drug Toxicity],​cocaine/to [Drug Toxicity],​illicit drug,​methamphetamine/to [Drug Toxicity],​nicotine/to [Drug Toxicity],​reactive oxygen metabolite/ec [Endogenous Compound]  Abstract: |
| 65 | PrintzC (2016) UCSF study: E-cigarettes are not helping smokers quit. Cancer; 122.  Keywords: demography,​*electronic cigarette,​food and drug administration,​health behavior,​human,​industry,​meta analysis,​note,​priority journal,​smoking,​smoking ban,​*smoking cessation,​systematic review,​tobacco dependence  Abstract: |
| 66 | FeirmanSP,​ LockD,​ CohenJE,​ et al (2016) Flavored Tobacco Products in the United States: A Systematic Review Assessing Use and Attitudes. Nicotine and Tobacco Research; 18.  Keywords: age,​*attitude to health,​case study,​consumer attitude,​correlational study,​electronic cigarette,​experimental study,​health belief,​human,​observational study,​prevalence,​priority journal,​qualitative research,​quasi experimental study,​review,​smokeless tobacco,​smoking,​systematic review,​*tobacco,​*tobacco use,​United States,​flavoring agent,​menthol,​bidis,​cigar,​*flavored tobacco product,​hookah,​kretek  Abstract: Objectives: We systematically reviewed research examining use of and attitudes toward nonmenthol- flavored tobacco products to provide information relevant to a decision to regulate these products in the future. Methods: To identify eligible studies,​ we searched PubMed,​ CINHAL,​ Embase,​ LILACS,​ and PsycINFO on September 19,​ 2013,​ without date restrictions. We obtained additional studies via gray literature searches,​ expert contacts,​ and hand-searching citations of included articles. We included participants of all ages. We conducted a qualitative synthesis for included studies. Results: The 32 studies included in this review exhibited substantial heterogeneity and were of varied methodological quality. Findings from observational,​ experimental,​ and quasiexperimental studies suggest that flavored tobacco use is associated with young age and that consumers may perceive flavored products more favorably than nonflavored products. Evidence from qualitative studies indicates that flavoring in tobacco is viewed favorably by users and nonusers of these products. Conclusions: The Food and Drug Administration has expressed interest in regulating flavored tobacco products. This systematic review strengthens the evidence base relating to this issue by synthesizing the literature from the United States on the use of and attitudes toward flavored tobacco. To address gaps in the literature,​ more research is needed to understand how flavoring impacts tobacco use over time. The evidence base would further be strengthened with the collection of brand-,​ flavor-,​ and product-specific data. |
| 67 | JhanjeeS (2016) Putting tobacco harm reduction in perspective: Is it a viable alternative?. Indian Journal of Medical Research; 143.  Keywords: cerebrovascular accident,​chronic obstructive lung disease,​economic aspect,​electronic cigarette,​*harm reduction,​human,​ischemic heart disease,​mouth cancer,​nicotine replacement therapy,​note,​premature mortality,​prevalence,​risk factor,​smokeless tobacco,​smoking ban,​smoking cessation program,​systematic review (topic),​tobacco dependence,​*tobacco use,​carbon monoxide,​cigarette smoke,​nicotine gum,​nitrogen oxide,​nitrosamine,​propylene glycol,​tar  Abstract: |
| 68 | IoakeimidisN,​ VlachopoulosC,​ TousoulisD (2016) Efficacy and safety of electronic cigarettes for smoking cessation: A critical approach. Hellenic Journal of Cardiology; 57.  Keywords: cardiovascular disease,​*electronic cigarette,​human,​lung disease,​medical literature,​public health,​publication,​randomized controlled trial (topic),​review,​risk factor,​safety,​*smoking cessation,​systematic review,​acetaldehyde,​formaldehyde,​glycerol,​heavy metal,​nicotine,​propylene glycol,​tobacco smoke  Abstract: |
| 69 | BernsteinSL (2016) Electronic cigarettes: More light,​ less heat needed. The Lancet Respiratory Medicine; 4.  Keywords: alternative medicine,​clinical effectiveness,​clinical research,​confidence interval,​*electronic cigarette,​hazard ratio,​human,​legal aspect,​marketing,​meta analysis (topic),​methodology,​motivation,​note,​priority journal,​randomized controlled trial (topic),​risk benefit analysis,​sensitivity analysis,​smoking,​smoking cessation,​systematic review (topic),​nicotine  Abstract: |
| 71 | VanderkamP,​ BoussageonR,​ UndernerM,​ et al (2016) Efficacy and security of electronic cigarette for tobacco harm reduction: Systematic review and meta-analysis. Presse Medicale; 45.  Keywords: article,​comparative effectiveness,​consultation,​*electronic cigarette,​harm reduction,​human,​meta analysis (topic),​quantitative analysis,​randomized controlled trial (topic),​smoking cessation,​systematic review (topic),​tobacco consumption,​nicotine,​placebo  Abstract: Context Smoking is the first cause of preventable death in France and in the world. Without help,​ it was shown that 80 % of smokers who try to quit smoking relapse after one month with a low long-term success rate. Smoking reduction can concern smokers who did not want to quit or failed in their attempt to weaning. The final aim is to increase attractiveness of drug therapies by developing new products,​ such as electronic cigarettes,​ that can compete cigarette without reproducing its harmful effects. Objective Assess the capacity of electronic cigarettes to reduce or stop tobacco use among regular smokers. Data sources Consultations MEDLINE and COCHRANE databases. Keywords: e-cigarette; electronic cigarettes; ENDD (electronic nicotine delivery system); ENDS (electronic nicotine delivery device); vaping were used. Trial selection Randomized controlled trials (RCTs) comparing the electronic cigarette with nicotine versus placebo device. Results Two randomized controlled trials were included in the quantitative analysis. The nicotine electronic cigarette users have tobacco consumption significantly decreased compared to the placebo group (RR: 1.30,​ 95 % CI [1.02 to 1.66]) at 6 months. Smoking cessation rate at 3 months was greater with the electronic cigarette contains nicotine (RR: 2.55,​ 95 % CI [1.31 to 4.98]). Limits The small number of RCTs included does not allow definitive conclusions about the effectiveness of electronic cigarettes,​ especially in the medium to long term. Conclusion The use of electronic cigarette with nicotine decreases tobacco consumption among regular smokers. Further studies are needed to specify electronic cigarettes safety profile and its ability to cause a reduction in consumption and a long-term cessation in smokers.Copyright © 2016 Elsevier Masson SAS |
| 72 | DavidT,​ TharyanP (2016) Electronic cigarettes for smoking cessation and reduction. Summary of the evidence and implications for public health programmes. Clinical Epidemiology and Global Health; 4.  Keywords: article,​behavior therapy,​*electronic cigarette,​Europe,​evidence based medicine,​human,​multicenter study (topic),​non communicable disease,​patient guidance,​priority journal,​*public health,​randomized controlled trial (topic),​risk factor,​*smoking cessation,​South Africa,​systematic review,​tobacco dependence/dt [Drug Therapy],​tobacco dependence/th [Therapy],​United States,​glycerol,​nicotine patch/ct [Clinical Trial],​nicotine patch/dt [Drug Therapy],​nicotine patch/td [Transdermal Drug Administration],​placebo,​propylene glycol  Abstract: |
| 73 | Lindson-HawleyN,​ Hartmann-BoyceJ,​ FanshaweTR,​ et al (2016) Interventions to reduce harm from continued tobacco use. Cochrane Database of Systematic Reviews; 2016.  Keywords: counseling,​electronic cigarette,​follow up,​*harm reduction,​health status,​human,​inhaler,​nicotine replacement therapy,​priority journal,​review,​self help,​*smoking cessation,​tobacco dependence/dt [Drug Therapy],​*tobacco use,​amfebutamone,​biological marker/ec [Endogenous Compound],​carbon,​carbon monoxide,​nicotine,​nicotine gum/cm [Drug Comparison],​nicotine gum/dt [Drug Therapy],​nicotine patch/cm [Drug Comparison],​nicotine patch/dt [Drug Therapy],​placebo,​tar,​varenicline  Abstract: Background: Although smoking cessation is currently the only guaranteed way to reduce the harm caused by tobacco smoking,​ a reasonable secondary tobacco control approach may be to try and reduce the harm from continued tobacco use amongst smokers unable or unwilling to quit. Possible approaches to reduce the exposure to toxins from smoking include reducing the amount of tobacco used,​ and using less toxic products,​ such as pharmaceutical,​ nicotine and potential reduced-exposure tobacco products (PREPs),​ as an alternative to cigarettes. Objectives: To assess the effects of interventions intended to reduce the harm to health of continued tobacco use,​ we considered the following specific questions: do interventions intended to reduce harm have an effect on long-term health status?; do they lead to a reduction in the number of cigarettes smoked?; do they have an effect on smoking abstinence?; do they have an effect on biomarkers of tobacco exposure?; and do they have an effect on biomarkers of damage caused by tobacco? Search methods: We searched the Cochrane Tobacco Addiction Group Trials Register (CRS) on the 21st October 2015,​ using free-text and MeSH terms for harm reduction,​ smoking reduction and cigarette reduction. Selection criteria: Randomized or quasi-randomized controlled trials of interventions to reduce the amount smoked,​ or to reduce harm from smoking by means other than cessation. We include studies carried out in smokers with no immediate desire to quit all tobacco use. Primary outcomes were change in cigarette consumption,​ smoking cessation and any markers of damage or benefit to health,​ measured at least six months from the start of the intervention. Data collection and analysis: We assessed study eligibility for inclusion using standard Cochrane methods. We pooled trials with similar interventions and outcomes (> 50% reduction in cigarettes a day (CPD) and long-term smoking abstinence),​ using fixed-effect models. Where it was not possible to meta-analyse data,​ we summarized findings narratively. Main results: Twenty-four trials evaluated interventions to help those who smoke to cut down the amount smoked or to replace their regular cigarettes with PREPs,​ compared to placebo,​ brief intervention,​ or a comparison intervention. None of these trials directly tested whether harm reduction strategies reduced the harms to health caused by smoking. Most trials (14/24) tested nicotine replacement therapy (NRT) as an intervention to assist reduction. In a pooled analysis of eight trials,​ NRT significantly increased the likelihood of reducing CPD by at least 50% for people using nicotine gum or inhaler or a choice of product compared to placebo (risk ratio (RR) 1.75,​ 95% confidence interval (CI) 1.44 to 2.13; 3081 participants). Where average changes from baseline were compared for different measures,​ carbon monoxide (CO) and cotinine generally showed smaller reductions than CPD. Use of NRT versus placebo also significantly increased the likelihood of ultimately quitting smoking (RR 1.87,​ 95% CI 1.43 to 2.44; 8 trials,​ 3081 participants; quality of the evidence: low). Two trials comparing NRT and behavioural support to brief advice found a significant effect on reduction,​ but no significant effect on cessation. We found one trial investigating each of the following harm reduction intervention aids: bupropion,​ varenicline,​ electronic cigarettes,​ snus,​ plus another of nicotine patches to facilitate temporary abstinence. The evidence for all five intervention types was therefore imprecise,​ and it is unclear whether or not these aids increase the likelihood of smoking reduction or cessation. Two trials investigating two different types of behavioural advice and instructions on reducing CPD also provided imprecise evidence. Therefore,​ the evidence base for this comparison is inadequate to support the use of these types of behavioural advice to reduce smoking. Four studies of PREPs (cigarettes with reduced levels of tar,​ carbon and nicotine,​ and in one case delivered using an electronically-heated cigarette smoking system) showed some reduction in exposure to some toxicants,​ but it is unclear whether this would substantially alter the risk of harm. We judged the included studies to be generally at a low or unclear risk of bias; however,​ there were some ratings of high risk,​ due to a lack of blinding and the potential for detection bias. Using the GRADE system,​ we rated the overall quality of the evidence for our cessation outcomes as 'low' or 'very low',​ due to imprecision and indirectness. A 'low' grade means that further research is very likely to have an important impact on our confidence in the estimate of effect and is likely to change the estimate. A 'very low' grade means we are very uncertain about the estimate. Authors' conclusions: People who do not wish to quit can be helped to cut down the number of cigarettes they smoke and to quit smoking in the long term,​ using NRT,​ despite original intentions not to do so. However,​ we rated the evidence contributing to the cessation outcome for NRT as 'low' by GRADE standards. There is a lack of evidence to support the use of other harm reduction aids to reduce the harm caused by continued tobacco smoking. This could simply be due to the lack of high-quality studies (our confidence in cessation outcomes for these aids is rated 'low' or 'very low' due to imprecision by GRADE standards),​ meaning that we may have missed a worthwhile effect,​ or due to a lack of effect on reduction or quit rates. It is therefore important that more high-quality RCTs are conducted,​ and that these also measure the long-term health effects of treatments.Copyright © 2016 The Cochrane Collaboration. Published by John Wiley & Sons,​ Ltd. |
| 77 | The Lancet Psychiatry (2015) Ceci n'est pas une cigarette. The Lancet Psychiatry; 2.  Keywords: editorial,​electronic cigarette,​human,​mental health,​meta analysis (topic),​passive smoking,​patient safety,​*smoking,​smoking ban,​systematic review (topic),​nicotine  Abstract: |
| 78 | MeernikC,​ GoldsteinAO (2015) A critical review of smoking,​ cessation,​ relapse and emerging research in pregnancy and post-partum. British Medical Bulletin; 114.  Keywords: article,​educational status,​electronic cigarette,​evidence based practice,​female,​harm reduction,​human,​*maternal smoking,​medicaid,​nicotine replacement therapy,​passive smoking,​practice guideline,​*pregnancy,​pregnant woman,​priority journal,​psychological aspect,​psychosocial care,​*puerperium,​randomized controlled trial (topic),​relapse,​*smoking cessation,​social aspect,​social status,​systematic review (topic),​systematic review,​tobacco use,​amfebutamone,​varenicline  Abstract: Introduction: Smoking during pregnancy causes adverse health outcomes. Though the prevalence of smoking among pregnant women has declined,​ postpartum relapse rates remain high and smoking-related maternal,​ fetal and infant morbidity and mortality remains a public health burden. Sources of data: A comprehensive literature search on smoking in pregnancy was conducted to provide a practical review for health professionals. Areas of agreement: Psychosocial support is an effective evidence-based treatment for pregnant women. Bio-psycho-socio factors that influence likelihood of quitting and remaining quit should be addressed. Areas of controversy: Electronic cigarettes are marketed as a harm reduction tool,​ but research on safety and effectiveness are lacking for pregnant women. Growing points: The safety and efficacy of pharmacotherapy for use among pregnant women remains unclear. Clinicians should increase discussions regarding all resources for tobacco use treatment and secondhand smoke (SHS) exposure during pregnancy and postpartum and offer psychosocial support to all pregnant women. Areas timely for developing research: Research on developing stronger tobacco control policies in low-and middle-income countries,​ increasing cessation and relapse prevention among pregnant smokers with mental health conditions and increasing the impact of evidence-based supports,​ such as the quitline,​ among pregnantwomen can decrease consumption of tobacco in pregnancy.Copyright © The Author 2015. Published by Oxford University Press. |
| 81 | Anonymous (2015) Electronic nicotine delivery systems. Pediatrics; 136.  Keywords: aerosol,​brain development,​*electronic cigarette,​environmental exposure,​epidemiological data,​food and drug administration,​funding,​government regulation,​health care planning,​health care policy,​health education,​health hazard,​high school student,​human,​meta analysis (topic),​neurotoxicity,​nicotine replacement therapy,​nonhuman,​parent counseling,​passive smoking,​patient counseling,​preventive medicine,​priority journal,​randomized controlled trial (topic),​review,​smoking cessation,​smoking cessation program,​smoking regulation,​symptom,​tax,​tobacco dependence/dt [Drug Therapy],​tobacco dependence/pc [Prevention],​tobacco dependence/th [Therapy],​United States,​aldehyde,​alkaloid,​flavoring agent,​glycerol,​nicotine/to [Drug Toxicity],​nicotine patch/ct [Clinical Trial],​nicotine patch/cm [Drug Comparison],​nicotine patch/dt [Drug Therapy],​nitrosamine,​placebo,​polycyclic aromatic hydrocarbon,​propylene glycol,​secondhand aerosol,​thirdhand aerosol  Abstract: Electronic nicotine delivery systems (ENDS) are rapidly growing in popularity among youth. ENDS are handheld devices that produce an aerosolized mixture from a solution typically containing concentrated nicotine,​ flavoring chemicals,​ and propylene glycol to be inhaled by the user. ENDS are marketed under a variety of names,​ most commonly electronic cigarettes and e-cigarettes. In 2014,​ more youth reported using ENDS than any other tobacco product. ENDS pose health risks to both users and nonusers. Nicotine,​ the major psychoactive ingredient in ENDS solutions,​ is both highly addictive and toxic. In addition to nicotine,​ other toxicants,​ carcinogens,​ and metal particles have been detected in solutions and aerosols of ENDS. Nonusers are involuntarily exposed to the emissions of these devices with secondhand and thirdhand aerosol. The concentrated and often flavored nicotine in ENDS solutions poses a poisoning risk for young children. Reports of acute nicotine toxicity from US poison control centers have been increasing,​ with at least 1 child death reported from unintentional exposure to a nicotine-containing ENDS solution. With flavors,​ design,​ and marketing that appeal to youth,​ ENDS threaten to renormalize and glamorize nicotine and tobacco product use. There is a critical need for ENDS regulation,​ legislative action,​ and counter promotion to protect youth. ENDS have the potential to addict a new generation of youth to nicotine and reverse more than 50 years of progress in tobacco control.Copyright © 2015 by the American Academy of Pediatrics. |
| 82 | LamC,​ WestA (2015) Are electronic nicotine delivery systems an effective smoking cessation tool?. Canadian Journal of Respiratory Therapy; 51.  Keywords: clinical effectiveness,​*drug delivery device,​*drug dependence treatment,​electronic cigarette,​human,​Medline,​mouth disease/co [Complication],​nausea/co [Complication],​randomized controlled trial (topic),​review,​satisfaction,​Scopus,​self report,​smoking,​*smoking cessation,​systematic review,​throat irritation/co [Complication],​treatment outcome,​Web of Science,​withdrawal syndrome,​carbon monoxide,​*nicotine/pr [Pharmaceutics],​*drug delivery device/am [Adverse Device Effect],​cigarette vapor,​*electronic nicotine delivery system,​mouth irritation/co [Complication],​*electronic nicotine delivery system/am [Adverse Device Effect]  Abstract: Background: Recent studies have estimated that 21% of all deaths over the past decade are due to smoking,​ making it the leading cause of premature death in Canada. To date,​ many steps have been taken to eradicate the global epidemic of tobacco smoking. Most recently,​ electronic nicotine delivery systems (ENDS) have become a popular smoking cessation tool. ENDS do not burn or use tobacco leaves,​ but instead vapourize a solution the user then inhales. The main constituents of the solution,​ in addition to nicotine when nicotine is present,​ are propylene glycol,​ with or without glycerol and flavouring agents. Currently,​ ENDS are not regulated,​ and have become a controversial topic. Objectives: To determine whether ENDS are an effective smoking cessation tool. Methods: A systematic literature search was conducted in February 2015 using the following databases: PubMed,​ Scopus and Web of Science Core Collection. Randomized controlled trials were the only publications included in the search. A secondary search was conducted by reviewing the references of relevant publications. Results: After conducting the primary and secondary search,​ 109 publications were identified. After applying all inclusion and exclusion criteria through abstract and full-text review,​ four publications were included in the present literature review. A low risk of bias was established for each included study using the Cochrane Collaboration risk of bias evaluation framework. Discussion: The primary outcome measured in all studies was selfreported abstinence or reduction from smoking. In three of the four studies,​ self-reported abstinence or reduction from smoking was verified by measuring exhaled carbon monoxide. In the remaining study,​ the primary outcome measured was self-reported desire to smoke and measured desire to smoke. All four studies showed promise that ENDS are an effective smoking cessation tool. Conclusions: While all publications included in the present review revealed that ENDS are effective smoking cessation aid,​ further evaluation of the potential health effects in long-term use of ENDS remains vital. |
| 83 | AbramsDB,​ NiauraR (2015) The importance of science-informed policy and what the data really tell us about e-cigarettes. Israel Journal of Health Policy Research; 4.  Keywords: *electronic cigarette,​emotion,​harm reduction,​*health care policy,​health impact assessment,​*health science,​human,​meta analysis (topic),​note,​priority journal,​public health,​randomized controlled trial (topic),​smoking cessation,​tobacco industry,​tobacco use,​United States  Abstract: A possible future end-game for cigarettes is explored in the context of the historical progress made to date by tobacco control. Despite good progress,​ there remains an urgent need to increase the use of proven tobacco control policies and practices for prevention and cessation. The problem is worse than previously thought and the 50th anniversary United States Surgeon General's report indicates the overwhelming majority of avoidable deaths are caused by combusting of tobacco,​ primarily cigarettes. The report highlights for the first time the addition of a harm minimization strategy to enhance proven tobacco control efforts and thus much more rapidly speed the obsolescence of cigarettes. Harm minimization can be two pronged. First,​ it can boost proven tobacco control polices to make cigarettes more expensive and less appealing and accessible to maximize the fact that cigarettes are orders of magnitude the most harmful of all tobacco delivery systems. Second,​ harm minimization can support use of substantially less harmful but appealing alternatives to substitute for lethal cigarettes for those users who are unable or unwilling to quit smoking. A future end-game might prudently manage emerging new products like e-cigarettes to help boost the difference in harm between them and lethal cigarettes. Harm minimization could help to accelerate the end of the century-long dominance of the cigarette in what has been called "the golden holocaust". Rather than these emerging delivery devices being used to replace lethal cigarettes in what might be termed a David versus Goliath strategy to disrupt the status quo,​ there is also legitimate concern that these new products could undermine historically successful tobacco control efforts,​ especially youth prevention,​ if allowed free reign. What can the data really tell us about the potential for e-cigarettes to be helpful or harmful? The emerging but limited scientific evidence and the inherent methodological constraints in study designs,​ points to the need for caution in prematurely interpreting results in a manner that could mislead policymakers.Copyright © Abrams and Niaura. |
| 84 | WaghelRC,​ BattiseDM,​ DuckerML (2015) Effectiveness of electronic cigarettes as a tool for smoking cessation or reduction. Journal of Pharmacy Technology; 31.  Keywords: anxiety,​article,​bibliographic database,​clinical effectiveness,​clinical trial (topic),​depression,​*electronic cigarette,​human,​hunger,​irritability,​monotherapy,​nicotine replacement therapy,​restlessness,​schizophrenia,​self report,​*smoking,​*smoking cessation,​systematic review,​tobacco dependence,​treatment duration,​withdrawal syndrome,​nicotine,​placebo  Abstract: Objective: To examine the evidence concerning effectiveness of electronic cigarettes (e-cigarettes) in smoking cessation or reduction and reduction in desire to smoke and withdrawal symptoms. Data Sources: A PubMed literature search through May 2014 was performed using search terms electronic cigarettes or e-cigarettes; smoking or tobacco; and efficacy or effectiveness limited to clinical trials in humans. Cochrane Library and International Pharmaceutical Abstracts were searched using the term electronic cigarettes. Relevant citations from identified articles were reviewed. Study Selection and Data Extraction: All English-language clinical trials assessing cessation,​ reduction in cigarette use,​ desire to smoke,​ and/or reduction in withdrawal symptoms with e-cigarettes were included. Data Synthesis: Of 7 trials,​ one showed similar 6-month abstinence rates between e-cigarettes and nicotine patches. Another trial reported 12.5% of participants achieving abstinence at 24 months with e-cigarettes. Three studies demonstrated significant percentages of patients maintaining >=50% reduction in cigarettes/day from baseline to 24 or 52 weeks. Another trial showed significant reductions in cigarette use from baseline through 52 weeks with e-cigarettes; no difference in abstinence rates beyond 12 weeks was found between nicotine and placebo e-cigarettes. Only 2 trials assessed reduction in desire to smoke or withdrawal symptoms,​ and results varied. Conclusions: The limited evidence available supports that e-cigarettes may be effective as monotherapy for smoking cessation and reduction. However,​ superiority to nicotine replacement therapy was not proven. Limited conclusions can be drawn regarding reduction in desire to smoke and withdrawal symptoms. The unknown longterm safety risk should also be considered.Copyright © The Author(s) 2014. |
| 85 | BrownJ (2015) A positive view on e-cigarettes. BMJ (Online); 351.  Keywords: clinical decision making,​clinical effectiveness,​Cochrane Library,​device therapy,​*electronic cigarette,​evidence based medicine,​health disparity,​health hazard,​human,​letter,​meta analysis (topic),​prevalence,​priority journal,​smoking cessation,​tobacco dependence/dt [Drug Therapy],​tobacco dependence/th [Therapy],​United Kingdom,​nicotine patch/dt [Drug Therapy]  Abstract: |
| 88 | LeeAHY,​ StaterBJ,​ CloseL,​ et al (2015) Are e-cigarettes effective in smoking cessation?. Laryngoscope; 125.  Keywords: article,​clinical effectiveness,​combustion,​human,​longitudinal study,​otolaryngologist,​particulate matter,​premature mortality,​priority journal,​randomized controlled trial (topic),​*smoking cessation,​systematic review (topic),​tobacco dependence,​*electronic cigarette,​nicotine patch  Abstract: |
| 89 | BornH,​ PerskyM,​ KrausDH,​ et al (2015) Electronic cigarettes: A primer for clinicians. Otolaryngology - Head and Neck Surgery (United States); 153.  Keywords: *electronic cigarette,​heart function,​human,​nicotine replacement therapy,​patient safety,​practice guideline,​randomized controlled trial (topic),​respiratory function,​review,​smoking cessation,​systematic review,​tobacco,​tobacco consumption,​tobacco use,​nicotine,​nicotine patch,​nitrosamine,​*electronic cigarette/ct [Clinical Trial]  Abstract: Objective. To introduce the otolaryngology community to the current state of research regarding electronic cigarettes,​ with special attention paid to mechanism,​ impact on health and addiction,​ and use in smoking cessation. Data Sources. Review of Google Scholar and PubMed databases using the keywords electronic cigarettes,​ e-cigs,​ e-cigarettes,​ and vaping. In addition,​ information from media sources as well as news outlets was evaluated to gauge public perception of research findings. Review Methods. Recent research and randomized controlled trials were prioritized. Conclusions. The landscape of electronic cigarette devices is evolving,​ as is the research regarding their risks and benefits. Utilization is rapidly increasing. It appears that older users employ them as a smoking cessation tool compared to younger users. The data are generally inconclusive regarding the benefits of electronic cigarettes for smoking cessation compared with other methods. Furthermore,​ the safety profile of electronic cigarettes is dynamic and difficult to fully ascertain. Implications for Practice. Patients with a variety of otolaryngologic conditions,​ including cancer,​ may benefit from frank discussion regarding electronic cigarettes. Furthermore,​ increasing patient inquiries regarding these devices are likely given their increasing popularity.Copyright © American Academy of Otolaryngology-Head and Neck Surgery Foundation 2015. |
| 90 | AnnamalaiA,​ SinghN,​ O'MalleySS (2015) Smoking use and cessation among people with serious mental illness. Yale Journal of Biology and Medicine; 88.  Keywords: anxiety,​behavior change,​biological activity,​bipolar disorder/dt [Drug Therapy],​bipolar disorder/th [Therapy],​clinical trial (topic),​cognitive therapy,​community mental health center,​drug efficacy,​drug withdrawal,​electronic cigarette,​evidence based practice,​human,​irritability,​major depression/dt [Drug Therapy],​major depression/th [Therapy],​*mental disease/dt [Drug Therapy],​*mental disease/th [Therapy],​mental disease/dt [Drug Therapy],​meta analysis (topic),​monotherapy,​mood disorder/si [Side Effect],​nausea/si [Side Effect],​nicotine replacement therapy,​nightmare/si [Side Effect],​paranoia/si [Side Effect],​patient counseling,​prescription,​prevalence,​psychosis/dt [Drug Therapy],​psychosis/th [Therapy],​qualitative analysis,​reinforcement,​review,​schizophrenia/dt [Drug Therapy],​schizophrenia/th [Therapy],​serious mental illness/dt [Drug Therapy],​smoking ban,​*smoking cessation,​suicide attempt/si [Side Effect],​*tobacco dependence/dt [Drug Therapy],​*tobacco dependence/th [Therapy],​tobacco dependence/dt [Drug Therapy],​treatment response,​unspecified side effect/si [Side Effect],​amfebutamone/ae [Adverse Drug Reaction],​amfebutamone/cb [Drug Combination],​amfebutamone/cm [Drug Comparison],​amfebutamone/dt [Drug Therapy],​clozapine/dt [Drug Therapy],​haloperidol/dt [Drug Therapy],​placebo,​varenicline/ae [Adverse Drug Reaction],​varenicline/ct [Clinical Trial],​varenicline/cb [Drug Combination],​varenicline/cm [Drug Comparison],​varenicline/cm [Drug Comparison],​varenicline/dt [Drug Therapy],​*serious mental illness/dt [Drug Therapy],​*serious mental illness/th [Therapy]  Abstract: Smoking rates in people with serious mental illness (SMI+) are disproportionately high compared to the general population. It is a leading contributor to the early mortality in this population. Smoking cessation rates are low in this group,​ though patients are motivated to quit. Unfortunately,​ health care providers do not always prioritize smoking cessation for this population. This review provides an overview of prevalence rates,​ biological effects that maintain smoking,​ and evidence-based treatments for smoking cessation in SMI. In addition,​ objective and qualitative data from a chart review of 78 patients with SMI prescribed smoking cessation treatment at one community mental health center are described. Of these,​ 30 (38.5 percent) were found to either quit (16/78) or reduce (14/78) smoking. Varenicline appeared to be particularly effective. Review of the literature and results of this study suggest that smoking cessation pharmacotherapies are effective for SMI patients and should be offered to those who smoke.Copyright © 2015,​ Yale Journal of Biology and Medicine Inc. All rights reserved. |
| 91 | PolosaR,​ CaponnettoP (2015) What to advise to respiratory patients intending to use electronic cigarettes. Discovery Medicine; 20.  Keywords: article,​cardiovascular disease,​chewing gum,​clinical trial (topic),​*electronic cigarette,​health care personnel,​human,​meta analysis (topic),​nicotine replacement therapy,​outcome assessment,​oxidative stress,​patient counseling,​physician,​*respiratory care practice,​risk factor,​smoking and smoking related phenomena,​smoking cessation,​carcinogen,​nicotine  Abstract: Electronic cigarettes (ECs) are being increasingly used as an attractive long-term alternative nicotine source to conventional cigarettes. This substitution is likely to improve health in regular EC users,​ and more and more respiratory patients using or intending to use ECs will be seeking professional medical advice. Unfortunately,​ the public's view of ECs is far from being clear with a great deal of ambiguity around the product and its intended use. Moreover,​ health-care professionals themselves do not seem to use an evidence-based approach when it comes to informing respiratory patients about ECs and many advise against their use. Evidence-based advice about ECs is provided here with the goal of improving counseling between physicians and their respiratory patients using or intending to use ECs. Regular EC use is unlikely to raise significant health concerns and can lead to health improvement in the respiratory patient who makes the switch.Copyright © Discovery Medicine. |
| 93 | BroseLS (2015) E-cigarettes may help smokers stop or reduce smoking. Evidence-Based Medicine; 20.  Keywords: clinical practice,​*electronic cigarette,​flavor,​harm reduction,​human,​nicotine replacement therapy,​note,​outcome assessment,​randomized controlled trial (topic),​*smoking cessation,​systematic review,​*tobacco dependence/th [Therapy],​United Kingdom,​glycerol,​nicotine,​propylene glycol,​tobacco smoke,​*electronic cigarette/ct [Clinical Trial]  Abstract: |
| 94 | BeghR,​ Lindson-HawleyN,​ AveyardP (2015) Does reduced smoking if you can't stop make any difference?. BMC Medicine; 13.  Keywords: article,​asthma,​cardiovascular risk,​chronic obstructive lung disease,​electronic cigarette,​*harm reduction,​health care planning,​health promotion,​heart infarction,​human,​ischemic heart disease,​lung cancer,​meta analysis (topic),​nicotine replacement therapy,​outcome assessment,​randomized controlled trial (topic),​risk assessment,​risk benefit analysis,​*smoking cessation,​tobacco dependence/dt [Drug Therapy],​world health organization,​cigarette smoke,​nicotine/ct [Clinical Trial],​nicotine/dt [Drug Therapy],​nicotine lozenge/ct [Clinical Trial],​nicotine lozenge/dt [Drug Therapy],​electronic cigarette/ct [Clinical Trial]  Abstract: Background: Promoting and supporting smoking reduction in smokers with no immediate intention of stopping smoking is controversial given existing fears that this will deter cessation and that reduction itself may not improve health outcomes. Discussion: Evidence shows that smokers who reduce the number of daily cigarettes smoked are more likely to attempt and actually achieve smoking cessation. Further,​ clinical trials have shown that nicotine replacement therapy benefits both reduction and cessation. Worldwide data suggests that 'non-medical' nicotine is more attractive to people who smoke,​ with electronic cigarettes now being widely used. Nevertheless,​ only one small trial has examined the use of electronic cigarettes to promote reduction,​ with direct evidence remaining inconclusive. It has been suggested that long-term reduced smoking may directly benefit health,​ although the benefits are small compared with cessation. Summary: The combined data imply that smoking reduction is a promising intervention,​ particularly when supported by clean nicotine; however,​ the benefits are only observed when it leads to permanent cessation.Copyright © 2015 Begh et al. |
| 95 | StubbsB,​ VancampfortD,​ BobesJ,​ et al (2015) How can we promote smoking cessation in people with schizophrenia in practice? A clinical overview. Acta Psychiatrica Scandinavica; 132.  Keywords: article,​behavior therapy,​clinical practice,​clinical trial (topic),​diabetes mellitus,​electronic cigarette,​group therapy,​health care disparity,​*health promotion,​human,​kinesiotherapy,​mental patient,​meta analysis (topic),​meta analysis,​nicotine replacement therapy,​priority journal,​psychopharmacotherapy,​psychosocial care,​*schizophrenia,​smoking,​*smoking cessation,​*smoking cessation program,​systematic review (topic),​tobacco dependence/dt [Drug Therapy],​tobacco dependence/th [Therapy],​transcranial direct current stimulation,​weight gain,​withdrawal syndrome,​amfebutamone/dt [Drug Therapy],​varenicline/dt [Drug Therapy]  Abstract: Objective: High rates of smoking and nicotine dependence are associated with increased physical comorbidity and premature death in people with schizophrenia. We conducted a clinical overview to establish how smoking cessation should be promoted in practice. Method: Systematic clinical review of major electronic databases from inception till November 2014. Results: A growing body of evidence supports pharmacological interventions to assist smoking cessation. The most promising evidence is for bupropion with several meta-analyses demonstrating its effectiveness. Currently,​ there is limited evidence demonstrating the effectiveness of nicotine replacement therapy (NRT) and varenicline,​ although this is likely to be due to the paucity of research. There are no consistent data to suggest that pharmacological interventions increase adverse events. Behavioural and psychosocial interventions also demonstrate promise,​ particularly when combined with pharmacotherapy. Careful monitoring of antipsychotic levels (in particular clozapine) is essential,​ and the promotion of physical activity may be useful to negate potential weight gain and diabetes risk following smoking cessation. Conclusion: Evidence from systematic reviews and meta-analyses suggests that smoking cessation interventions are effective in people with schizophrenia,​ although more long-term research is required. Promoting smoking cessation should be given utmost priority in clinical practice,​ and we offer practical strategies to facilitate this.Copyright © 2015 John Wiley & Sons A/S. Published by John Wiley & Sons Ltd |
| 97 | FoleyNC,​ LindsayP (2015) The use of electronic cigarettes is not associated with cessation of smoking: A systematic review and meta-analysis. International Journal of Stroke; 10.  Keywords: *smoking,​*systematic review,​*meta analysis,​*Canadian,​*cerebrovascular accident,​*electronic cigarette,​smoking cessation,​human,​Scopus,​Medline,​confidence interval,​risk,​adult,​follow up,​community,​longitudinal study,​cross-sectional study,​sample size,​education,​ethnicity,​abstinence,​data base,​Web of Science,​nicotine,​placebo,​nicotine patch  Abstract: Background: The use of electronic cigarettes (e-cigarettes) has increased significantly over the past several years. In addition to the claim that the e-version is a less harmful alternative to conventional cigarettes,​ another of their purported benefits is a smoking cessation aide. Methods: Relevant studies were identified through a literature search of Pubmed,​ EMBASE,​ Scopus,​ and ISI Web of Science databases,​ published up toMarch 2015. Studies were included if they were controlled (in design or analysis) and examined the relationship between electronic cigarette use and smoking cessation (abstinence for at least 30 days) in healthy,​ community-dwelling adults who were current smokers,​ or who had recently quit. Pooled analysis was conducted using a random effects approach and expressed as an odds ratio (OR) with 95% confidence interval (CI). Results: The results from 4 studies were included (2 RCTs,​ 1 longitudinal study,​ and 1 cross-sectional study). Total sample size was 3,​742 participants (1,​414 e-cigarette users). The mean age of participants was 42 years. The mean/median number of cigarettes smoked per day ranged from 14 to 20. The study contrasts in the 2 RCTs were active e-cigarettes (i.e.,​ nicotine containing) vs. placebo e-cigarette (i.e.,​ no nicotine) vs. nicotine patches; and e-cigarettes (2 levels of nicotine) vs. placebo e-cigarettes. In the 2 non-RCTs,​ the analyses were adjusted for demographic variables age,​ sex ethnicity,​ education,​ and baseline smoking (number of cigarettes smoked/day). Lengths of follow-up were 6 months (n = 1),​ and one year (n = 2). The use of e-cigarettes did not significantly increase the odds of successfully quitting smoking (OR = 1.22,​ 95% CI 0.73-2.03,​ p = 0.45). Conclusions: Although little empirical evidence exists,​ the use of electronic cigarettes was not associated with an increased odds of successful smoking cessation. |
| 98 | AllehebiRO,​ KhanM,​ StanbrookMB (2015) Efficacy and safety of electronic cigarettes for smoking cessation: A systematic review. American Journal of Respiratory and Critical Care Medicine; 191.  Keywords: *electronic cigarette,​*smoking cessation,​*systematic review,​*American,​*society,​*safety,​abstinence,​prevalence,​tobacco,​validity,​statistical significance,​smoking,​meta analysis,​atrial fibrillation,​aspiration pneumonia,​dyspnea,​human,​case report,​death,​throat irritation,​coughing,​adverse drug reaction,​comparative study,​risk,​therapy,​devices,​placebo,​nicotine patch,​nicotine  Abstract: Rationale: Electronic-cigarettes (e-cigarettes) are battery-powered electronic nicotine delivery devices designed to deliver nicotine in a similar manner to tobacco without tobacco's other harmful constituents. We systematically reviewed the evidence to date regarding the efficacy and safety of e-cigarettes. Methods: We searched MEDLINE and EMBASE from 1946 to May 2014. Studies of efficacy were included if they enrolled current smokers and compared e-cigarettes to placebo,​ active control or no therapy. Studies of safety were included regardless of design if they reported any adverse events associated with e-cigarette use. Results: Of 4569 abstracts identified,​ 297 articles underwent full-text review. For efficacy,​ 4 studies (2 randomized trials,​ 2 uncontrolled before-and-after studies) met inclusion criteria. For safety,​ 22 articles met inclusion criteria. Meta-analysis showed that point prevalence abstinence was significantly better for e-cigarettes vs. placebo at 1 month (RR 1.71,​ 95% CI 1.08-2.72,​ I2 = 0%). However,​ differences for point prevalence abstinence did not reach statistical significance at 3 months (RR 1.95,​ 95% CI 0.74-5.13,​ I2 = 65%) or 6 months (RR 1.32,​ 95% CI 0.59-2.93,​ I2 = 59%),​ with large heterogeneity between studies rendering the validity of these pooled estimates uncertain. The only study to evaluate continuous abstinence found low rates at 6 months,​ with no significant differences seen between e-cigarettes compared with placebo (7.3% vs. 4.1%,​ RR 1.77,​ 95% CI 0.54-5.77) or open-label nicotine patch (7.3% vs. 5.8%,​ RR 1.26,​ 95% CI 0.68-2.34). Respiratory adverse effects among e-cigarette users included dry cough (incidence range 26-32%),​ throat irritation (7-32%),​ and shortness of breath (2- 20%),​ although incidence of these events tended to decrease over time. Case reports have documented serious adverse events in e-cigarette users including death,​ lipoid pneumonia,​ and recurrent atrial fibrillation. In comparative studies,​ incidence of serious adverse events did not differ between e-cigarettes and placebo e-cigarettes (19.7% vs. 13.9%,​ RR 1.36,​ 95% CI 0.54-3.42),​ but were more frequent with e-cigarettes than open-label nicotine patch (19.7% vs. 11.8%,​ RR 1.97,​ 95% CI 1.05-3.68). Conclusions: Electronic cigarettes achieve higher rates of smoking cessation at 1 month than placebo,​ but limited available data suggest that this effect may not be sustained over longer time periods. E-cigarettes are associated with frequent short-term respiratory adverse events and may pose a higher risk of serious adverse events than nicotine patch. Given the paucity of existing data,​ long-term studies of the efficacy and safety of e-cigarettes are needed to determine their possible role in smoking cessation. |
| 100 | MeoSA,​ AlAsiriSA (2014) Effects of electronic cigarette smoking on human health. European Review for Medical and Pharmacological Sciences; 18.  Keywords: airway obstruction,​allergic airway inflammation,​article,​brain disease,​bronchus,​burn,​cancer risk,​coughing,​cytokine release,​dizziness,​dry eye,​*electronic cigarette,​exhalation,​eye disease,​gastrointestinal disease,​gene expression,​genital system disease,​hazard assessment,​headache,​*health,​health hazard,​human,​inflammation,​lung cancer,​mediator release,​Medline,​mucosal dryness,​nausea,​respiratory tract disease,​*smoking,​systematic review,​vomiting,​Web of Science,​cytokine/ec [Endogenous Compound],​nitric oxide/ec [Endogenous Compound],​*electronic cigarette smoking,​upper respiratory tract irritation  Abstract: OBJECTIVE: Electronic cigarette smoking is gaining dramatic popularity and is steadily spreading among the adolescents,​ high income,​ urban population around the world. The aim of this study is to highlight the hazards of e-cigarette smoking on human health. MATERIALS AND METHODS: In this study,​ we identified 38 published studies through a systematic database searches including ISI-web of science and pub-med. We searched the related literature by using the key words including Electronic cigarette,​ E-cigarette,​ E-vapers,​ incidence,​ hazards. Studies in which electronic cigarette smoking hazards was investigated were included in the study. No limitations on publication status,​ study design of publication were implemented. Finally we included 28 publications and remaining 10 were excluded. RESULTS: E-smoking can cause,​ nausea,​ vomiting,​ headache,​ dizziness,​ choking,​ burn injuries,​ upper respiratory tract irritation,​ dry cough,​ dryness of the eyes and mucous membrane,​ release of cytokines and pro-inflammatory mediators,​ allergic airway inflammation,​ decreased exhaled nitric oxide (FeNO) synthesis in the lungs,​ change in bronchial gene expression and risk of lung cancer. CONCLUSIONS: Electronic cigarettes are swiftly promoted as an alternative to conventional cigarette smoking,​ although its use is highly controversial. Electronic cigarettes are not a smoking cessation product. Non-scientific claims about e-cigarettes are creating confusion in public perception about e-cigarette and people believe that e-cigarettes are safe and less addictive,​ but its use is unsafe and hazardous to human health. E-cigarette smoking should be regulated in the same way as traditional cigarettes and must be prohibited to children and adolescents. |
| 101 | ProchaskaJJ (2014) Quitting smoking is associated with long term improvements in mood. BMJ (Online); 348.  Keywords: anxiety,​brain function,​disease association,​disease predisposition,​drug surveillance program,​drug use,​editorial,​exercise,​follow up,​human,​intermethod comparison,​long term exposure,​major depression,​mental disease,​*mental health,​meta analysis (topic),​*mood change,​*mood disorder/si [Side Effect],​mood disorder/si [Side Effect],​neurotransmitter release,​nicotine replacement therapy,​observational study,​posttraumatic stress disorder,​priority journal,​quality of life,​randomized controlled trial (topic),​recurrent disease,​risk assessment,​schizophrenia,​*smoking cessation,​social support,​stress,​systematic review (topic),​withdrawal syndrome,​amfebutamone/ae [Adverse Drug Reaction],​amfebutamone/ct [Clinical Trial],​electronic cigarette,​nicotine,​varenicline/ae [Adverse Drug Reaction],​varenicline/ct [Clinical Trial]  Abstract: |
| 102 | RahmanMA,​ HannN,​ WilsonA,​ et al (2014) Electronic cigarettes: Patterns of use,​ health effects,​ use in smoking cessation and regulatory issues. Tobacco Induced Diseases; 12.  Keywords: airway obstruction,​cytotoxicity,​drug delivery device,​geographic distribution,​*government regulation,​health care policy,​*health impact assessment,​human,​legal aspect,​nicotine replacement therapy,​pneumonia,​priority journal,​randomized controlled trial (topic),​recreation,​review,​sex difference,​smoking,​*smoking cessation,​social status,​systematic review,​tobacco dependence,​acrolein,​*electronic cigarette,​formaldehyde,​tobacco smoke,​toxic substance,​toxin  Abstract: Background: Electronic cigarettes (e-cigarettes) are battery-powered devices that vaporize a liquid solution to deliver a dose of inhaled nicotine to the user. There is ongoing debate regarding their regulation. Objectives: This comprehensive narrative review aimed to discuss key issues including usage patterns,​ health effects,​ efficacy in smoking cessation and regulatory concerns with a view to informing future regulation and research agendas. Methods: PubMed,​ Scopus and Web of Science databases were searched using the terms (electronic cigarettes OR e-cigarettes) for articles in English,​ relevant to humans and published during January 2009-January 2014. Results: The literature search revealed 37 relevant articles. Findings suggest that e-cigarettes are mostly used by middle-aged current smokers,​ particularly males,​ to help them for quitting or for recreation. E-cigarettes contain very low levels of multiple toxic substances such as formaldehyde and acrolein,​ but these levels are many times lower than those found in cigarettes. They were found to have effectiveness in aiding smoking cessation to a limited degree. Debate continues regarding regulating their use for cessation versus heavy restrictions to control recreational use on the basis that it perpetuates nicotine addiction. Conclusions: The cytotoxicity and long term health effects of e-cigarettes are unknown. Nevertheless the e-cigarette market continues to expand,​ largely driven by middle-aged smokers who claim to be using e-cigarettes in an attempt to reduce or quit smoking. E-cigarettes may have some potential as smoking cessation aids and,​ in the researchers' view,​ should therefore be subject to further research and regulation similar to other nicotine replacement therapies.Copyright © 2014 Rahman et al.; licensee BioMed Central. |
| 103 | FranckC,​ BudlovskyT,​ WindleSB,​ et al (2014) Electronic cigarettes in North America: History,​ use,​ and implications for smoking cessation. Circulation; 129.  Keywords: article,​awareness,​Cochrane Library,​coughing/si [Side Effect],​drug efficacy,​drug safety,​dyspnea/si [Side Effect],​human,​inhalation,​medical ethics,​medical history,​Medline,​meta analysis,​motivation,​North America,​practice guideline,​priority journal,​quality control,​randomized controlled trial (topic),​risk reduction,​*smoking,​*smoking cessation,​smoking habit,​systematic review,​throat irritation/si [Side Effect],​*tobacco dependence/dt [Drug Therapy],​tobacco dependence/dt [Drug Therapy],​xerostomia/si [Side Effect],​*electronic cigarette/ae [Adverse Drug Reaction],​*electronic cigarette/dt [Drug Therapy],​nicotine,​nicotine patch,​placebo  Abstract: Background: Designed to mimic the look and feel of tobacco cigarettes,​ electronic cigarettes (e-cigarettes) may facilitate smoking cessation. However,​ the efficacy and safety of e-cigarette use for this purpose remain poorly understood. Our objectives were to review the available data on the efficacy and safety of e-cigarettes for smoking cessation and to consider issues relevant to the context in which they are used,​ including product awareness and regulatory and ethical concerns. Methods and Results: We systematically searched PubMed for randomized controlled trials and uncontrolled,​ experimental studies involving e-cigarettes. Included studies were limited to English or French language reports. Quality assessment was performed according to the Cochrane Risk of Bias tool. We identified 169 publications,​ of which 7 studies were included. Studies have concluded that e-cigarettes can help reduce the number of cigarettes smoked and may be as effective for smoking cessation as the nicotine patch. Although there is a lack of data concerning the safety and efficacy of e-cigarettes as a smoking cessation therapy,​ available evidence showed no significant difference in adverse event rates between e-cigarettes and the nicotine patch. E-cigarettes are widely used among smokers attempting to quit. However,​ significant international variation remains in the regulatory mechanisms governing the sale and distribution of e-cigarettes. Ethical concerns surround the use of e-cigarettes among minors and their potential to undermine efforts to reduce cigarette smoking. Conclusion: Given the limited available evidence on the risks and benefits of e-cigarette use,​ large,​ randomized,​ controlled trials are urgently needed to definitively establish their potential for smoking cessation. © 2014 American Heart Association,​ Inc. |
| 104 | RatschenE (2014) Electronic cigarettes in mental health settings - Solving a conundrum?. Psychiatric Bulletin; 38.  Keywords: article,​*electronic cigarette,​human,​*mental disease,​mental health center,​practice guideline,​randomized controlled trial (topic),​*smoking,​smoking ban,​smoking cessation,​systematic review (topic)  Abstract: Electronic cigarettes (e-cigarettes),​ have recently been the focus of much attention and debate. This article attempts to highlight their relevance and potential importance for mental health settings,​ with a focus on in-patient units. To do so,​ the complexities involved in smoking among people with mental disorder,​ the debate surrounding e-cigarettes,​ and their potential to be utilised as a smoking cessation or temporary abstinence aid in the context of smoke-free policies and new National Institute for Health and Care Excellence guidance for smoking cessation in mental health settings,​ will be discussed and synthesised below.Copyright © 2014 The Author. |
| 105 | ChapmanS (2014) E-cigarettes: The best and the worst case scenarios for public health - An essay by Simon Chapman. BMJ (Online); 349.  Keywords: *electronic cigarette,​England,​harm reduction,​health care access,​human,​Korea,​market,​New Zealand,​priority journal,​*public health,​public health message,​review,​smoking,​smoking cessation,​smoking regulation,​systematic review (topic),​tobacco industry,​tobacco use,​cigarette smoke,​nicotine  Abstract: |
| 106 | RahmanMA,​ HannNR,​ WilsonAM,​ et al (2014) Electronic cigarettes are effective for smoking cessation: Evidence from a systematic review and meta-analysis. Circulation; 130.  Keywords: *smoking cessation,​*systematic review,​*meta analysis,​*medical society,​*resuscitation,​human,​smoking,​model,​hypothesis,​gender,​randomized controlled trial,​implantable cardioverter defibrillator,​study design,​consumer,​effect size,​data base,​cross-sectional study,​cohort analysis,​risk,​Medline,​death,​*electronic cigarette,​nicotine  Abstract: Abstract Introduction: Smoking is the leading cause of preventable death worldwide. Finding effective interventions for smoking cessation has proven difficult and existing interventions have limited consumer appeal. Electronic cigarettes (e-cigarettes) are becoming increasingly popular and a possible role for them in smoking cessation is being debated. Our objective was therefore to analyse existing research to investigate whether use of e-cigarettes is an effective smoking cessation method. Hypothesis: We assessed the hypothesis that use of e-cigarettes is an effective smoking cessation method. Methods: A systematic review of articles in English of any publication date was conducted by searching PubMed,​ Web of Knowledge and Scopus databases. Published studies investigating the effectiveness of e-cigarettes for smoking cessation among current smokers were included. Studies were systematically reviewed,​ and meta-analyses were conducted using the Mantel-Haenszel fixed-effect and random-effects models. Heterogeneity and quality of the selected studies were also evaluated. Results: Six studies were selected,​ including two randomised controlled trials,​ two cohort studies and two cross-sectional studies,​ and included 7,​551 participants. Meta-analyses included 1,​242 participants on whom complete smoking cessation data was available. Of these,​ 224 (18%) reported smoking cessation after using nicotine-enriched e-cigarettes for a minimum period of six months. Use of such e-cigarettes was positively associated with smoking cessation with a pooled Effect Size of 0.20 (95%CI 0.11-0.28). Nicotine filled e-cigarettes were more effective in achieving cessation compared to those without nicotine (pooled Risk Ratio 2.29,​ 95%CI 1.05-4.97). Use of e-cigarettes was also effective in reducing smokers' daily cigarette consumption. The studies included were heterogeneous,​ (I2=93%,​ p<0.001). A meta-regression model showed that 98% of this heterogeneity was caused by study design and gender variation. Conclusions: In conclusion,​ available literature suggests that the use of e-cigarettes may be an effective alternate smoking cessation method. Further research is required to investigate this among both genders. |
| 109 | PrattA,​ SuL,​ Audera-LopezC,​ et al (2013) The rise of e-cigarettes: An emerging threat to the tobacco endgame?. Respiratory Medicine; 107.  Keywords: *tobacco,​*health care planning,​*public health,​health,​systematic review (topic),​devices,​smoking,​imitation,​safety,​methodology,​tobacco dependence,​protection,​smoking cessation,​government,​human,​population,​marketing,​systematic review,​policy,​world health organization,​China,​*electronic cigarette  Abstract: Background: Electronic cigarettes (e-cigarettes) first developed in China in 2004are gaining popularity and the number of e-cigarette users is growing rapidly. E-cigarette manufacturers and supporters of the devices argue that e-cigarettes are a safe alternative to tobacco smoking,​ and that they may aid smoking cessation. However,​ there is currently insufficient scientific evidence to prove their safety and efficacy. Furthermore,​ at the 5th session of the WHO FCTC Conference of Parties,​ concerns were raised that ENDs have been aggressively advertised and marketed as imitation or substitute tobacco products and as cessation devices around the world. Parties called for WHO to gather more evidence on whether e-cigarettes are safe,​ their efficacy as cessation aids,​ and that they don' t act as a gateway to nicotine addiction for young people. Objectives: The objective of this paper is to discuss health concerns on ecigarettes and to provide an overview of recent responses and regulations made by governments to ensure protection from potential health threats to individuals and populations. It will also examine the different marketing strategies employed by manufacturers in order to highlight the need for regulation of these products. Methodology: Systematic review of existing online information. Results: This paper consolidates existing information and evidence and outlines priorities for research and policy development. |
| 111 | TahiriM,​ MottilloS,​ JosephL,​ et al (2011) Unconventional smoking cessation aids: A metaanalysis of randomized controlled trials. American Journal of Epidemiology; 173.  Keywords: *meta analysis,​*smoking cessation,​*epidemiology,​*randomized controlled trial (topic),​smoking,​acupuncture,​patient,​human,​cigarette smoking,​hypnosis,​Cochrane Library,​Medline,​PsycINFO,​data base,​prevalence,​abstinence,​model,​risk,​confidence interval  Abstract: Background: Unconventional smoking cessation aids,​ including acupuncture,​ electronic (E)-cigarettes,​ hypnotherapy,​ and rapid smoking are increasingly being used as an alternative to pharmacological and behavioral interventions for smoking cessation. Randomized controlled trials (RCTs) investigating these unconventional aids have reported widely varying estimates of their efficacy. Objective: To carry out a meta-analysis to determine the efficacy of unconventional smoking cessation aids and to compare these aids to one another. Methods: We systematically searched the Cochrane Library,​ EMBASE,​ Medline,​ and PsycINFO databases through June 2010 for RCTs investigating acupuncture,​ E-cigarette,​ hypnotherapy,​ and rapid smoking for smoking cessation. We only included RCTs that reported cessation outcomes as point prevalence or continuous abstinence at 6 or 12 months. We used random-effect modeling for our meta-analysis. Results: A total of 14 RCTs were identified,​ of which 6 investigated acupuncture (823 patients),​ 4 investigated hypnosis (273 patients),​ and 4 investigated rapid smoking (99 patients). No RCTs investigating Ecigarettes met our inclusion criteria. The estimated mean treatment effects were acupuncture (odds ratio (OR) = 3.53; 95% confidence interval (CI) = 1.03,​ 12.07),​ hypnotherapy (OR = 4.55; 95% CI = 0.98,​ 21.01),​ and rapid smoking (OR = 4.26; 95% CI = 1.26,​ 14.38). Conclusion: Acupuncture and rapid smoking substantially increase the odds of smoking cessation. Although hypnosis may aid in smoking cessation,​ there is insufficient evidence to draw strong conclusions regarding its efficacy. RCTs on E-cigarettes are needed to evaluate their efficacy. |
| 119 | Carrie D Patnode,​ Jillian T Henderson,​ Jamie H Thompson,​ et al (2015) . #journal#; .  Keywords:   Abstract: BACKGROUND: Tobacco use is the leading preventable cause of disease,​ disability,​ and death in the United States. Interventions to help adults quit smoking might stop or reduce tobacco-related illness.,​ PURPOSE: To systematically review evidence for the effectiveness and safety of pharmacotherapy and behavioral tobacco cessation interventions among adults,​ including pregnant women and those with mental health conditions,​ and to conduct a de novo search for primary evidence related to electronic nicotine delivery systems for adults.,​ METHODS: We conducted a review of reviews and searched for existing systematic reviews published through August 1,​ 2014 in the following databases and organizations' websites: PubMed,​ PsycInfo,​ the Database of Abstracts of Reviews of Effects,​ the Cochrane Database of Systematic Reviews,​ the Centre for Reviews and Dissemination Health Technology Assessment,​ the Agency of Healthcare Research and Quality,​ British Medical Journal Clinical Evidence,​ the Canadian Agency for Drugs and Technologies in Health,​ Center for Disease Control and Prevention's Guide to Community Preventive Services,​ the Institute of Medicine,​ the National Institute for Health and Clinical Excellence,​ the National Health Service Health Technology Assessment Programme,​ and the Surgeon General. We included reviews that were published in the English language that systematically reported the effects of tobacco cessation interventions on health,​ cessation,​ or adverse outcomes. We excluded nonsystematic meta-analyses and narrative reviews and those that focused on harm reduction or relapse prevention. We conducted an a priori search for primary trial evidence related to the effectiveness and safety of electronic nicotine delivery systems (ENDS) (through March 1,​ 2015) and a search for pharmacotherapy among pregnant women (through August 15,​ 2014) to supplement the review of reviews methodology. Two investigators independently reviewed abstracts and full-text articles against a set of a priori inclusion and quality criteria. Discrepancies were resolved by consensus. One reviewer abstracted data into an evidence table and a second reviewer checked these data. We grouped reviews based on population (general adults,​ pregnant women,​ individuals with mental health conditions) and intervention (pharmacotherapy,​ behavioral,​ or combined interventions). We identified one or more reviews within each population and intervention subgroup that represented the most current and applicable evidence to serve as the basis for the main findings ("primary" reviews) and discussed complementary and discordant findings from other included reviews as necessary. We did not reanalyze any of the individual study evidence; we presented pooled analyses and existing point estimates from included reviews.,​ RESULTS: We included 54 systematic reviews,​ 22 of which served as the basis for the primary findings. Among adults,​ nine reviews addressed the efficacy and/or harms of nicotine replacement therapy (NRT),​ bupropion hydrochloride sustained release (bupropion SR),​ and/or varenicline. None of these reviews reported on health outcomes. All three medications were found to be effective in increasing smoking quit rates compared with placebo or nondrug arms at 6 or more months followup. The pooled risk ratio (RR) for abstinence for NRT was 1.60 (95% confidence interval [CI],​ 1.53 to 1.68); for bupropion SR,​ RR 1.62 (95% CI,​ 1.49 to 1.76); and for varenicline,​ 2.27 (95% CI,​ 2.02 to 2.55). Combined NRT versus a single form of NRT showed a statistically significantly greater cessation effect in pooled analysis (RR 1.34 [95% CI,​ 1.18 to 1.51]). None of the drugs were associated with major cardiovascular adverse events,​ although NRT produced higher rates of all cardiovascular events (driven by minor events). One review on combined pharmacotherapy and behavioral interventions reported a relative increase in quitting by 82 percent versus nonpharmacotherapy usual care (RR 1.82 [95% CI,​ 1.66 to 2.00]). We included an additional 33 reviews that addressed behavioral tobacco cessation treatments among adults,​ including those that focused on specific subpopulations such as older adults. Compared with various controls,​ behavioral interventions such as in-person advice and support from clinicians,​ self-help materials,​ and telephone counseling had modest,​ but significantly increased,​ relative smoking cessation at 6 or more months (18% to 96%). For example,​ the pooled RR of physician advice versus no advice was 1.76 (95% CI,​ 1.58 to 1.96) for smoking cessation at 6 or more months followup. Only two trials addressed the efficacy and harms related to the use of electronic cigarettes and these trials suggested no benefit on smoking cessation among smokers intending to quit. We included eight reviews that focused on pregnant women that found significant benefits for perinatal health,​ including increased birth weight and reduced preterm birth. These benefits were evident with behavioral interventions,​ and suggested by data from some of the NRT trials,​ although that evidence was limited. Cessation during late pregnancy was greater among women receiving any type of behavioral intervention,​ with evidence most clear for counseling. Rates of validated cessation among women allocated to NRT (5% to 24%) compared with placebo (0% to 15%) were not statistically different,​ although few studies contributed data. Our reviews among individuals with depression or schizophrenia provided limited trial evidence on the efficacy of pharmacotherapy or behavioral interventions. There was,​ however,​ some evidence of a benefit for bupropion among those with schizophrenia and the addition of a mood management component to behavioral interventions for smokers with depression.,​ CONCLUSIONS: This review of reviews suggests that behavioral interventions and pharmacotherapy,​ alone or in combination,​ are effective in helping to reduce rates of smoking among the general adult population. Behavioral interventions,​ in particular,​ can assist pregnant women to stop smoking. Data on the effectiveness and safety of electronic nicotine delivery systems are limited. Future research should focus on direct comparisons between different combinations and classes of drugs; the incidence of serious adverse events related to medications for cessation; the efficacy and safety of ENDS; and pharmacotherapies for pregnant women and those with mental health conditions including evidence on health outcomes. |
| 124 | Sarah Wolf,​ Senushi O'Sullivan,​ Roselynn Dean,​ et al (2019) Does utilization of electronic cigarettes facilitate smoking cessation compared to other interventions?.. The Journal of the Oklahoma State Medical Association; 112.  Keywords:   Abstract: Clinical Question: Does utilization of electronic cigarettes facilitate smoking cessation compared to other interventions?,​ Authors: Sarah Wolf MD,​ PGY-1; Senushi O'Sullivan MD,​ PGY-2;Roselynn Dean MD,​ PGY-3; Tomas Owens MD.,​ Faculty Mentor: Tomas Owens,​ MD.,​ Residency Program: Integris Great Plains Family Medicine Residency Program,​ Oklahoma City,​ Oklahoma.,​ Answer: Debatable. With conflicting data and some evidence of e-cigarettes leading to on-going nicotine use,​ there is not a clear benefit to utilizing e-cigarettes for smoking cessation.,​ Level of Evidence for the answer: B.,​ Search Terms: smoking cessation,​ electronic cigarettes,​ e-cigarettes.,​ Date Search was Concluded: 24 April 2019 Inclusion and Exclusion Criteria.,​ Inclusion criteria: Studies directly comparing smoking cessation with utilization of electronic cigarettes compared to control group.,​ Exclusion criteria: Studies that were systematic reviews,​ published since 2015,​ and studies comparing smoking cessation with anything other than electronic cigarettes. |
| 125 | Caitlin Notley,​ Tracey J Brown,​ Linda Bauld,​ et al (2019) Development of a Complex Intervention for the Maintenance of Postpartum Smoking Abstinence: Process for Defining Evidence-Based Intervention.. International journal of environmental research and public health; 16.  Keywords:   Abstract: Relapse to tobacco smoking for pregnant women who quit is a major public health problem. Evidence-based approaches to intervention are urgently required. This study aimed to develop an intervention to be integrated into existing healthcare. A mixed methods approach included a theory-driven systematic review identifying promising behaviour change techniques for targeting smoking relapse prevention,​ and qualitative focus groups and interviews with women (ex-smokers who had remained quit and those who had relapsed),​ their partners and healthcare professionals (N = 74). A final stage recruited ten women to refine and initially test a prototype intervention. Our qualitative analysis suggests a lack,​ but need for,​ relapse prevention support. This should be initiated by a trusted 'credible source'. For many women this would be a midwife or a health visitor. Support needs to be tailored to individual needs,​ including positive praise/reward,​ novel digital and electronic support and partner or social support. Advice and support to use e cigarettes or nicotine replacement therapy for relapse prevention was important for some women,​ but others remained cautious. The resulting prototype complex intervention includes face-to-face support reiterated throughout the postpartum period,​ tailored digital and self-help support and novel elements such as gifts and nicotine replacement therapy (NRT). |
| 127 | Michael D Klein,​ Natasha A Sokol,​ Laura R Stroud (2019) Electronic Cigarettes: Common Questions and Answers.. American family physician; 100.  Keywords:   Abstract: Electronic cigarettes (e-cigarettes) are popular devices designed to heat a liquid solution,​ often containing nicotine,​ that generates an inhaled aerosol,​ or vapor. e-Cigarettes have been marketed as healthier alternatives to traditional cigarettes. Thus,​ most adult users are current or former smokers who use e-cigarettes to reduce or quit cigarette smoking. Switching completely from cigarettes to e-cigarettes is associated with reduced toxicant exposure and reduced short-term respiratory symptoms; however,​ long-term health effects of e-cigarettes are unknown. Although a recent randomized trial suggests that e-cigarettes may promote smoking cessation,​ systematic reviews have had low certainty of evidence regarding cessation. e-Cigarettes pose several potential health risks,​ including exposure to heavy metals and toxicants,​ and nicotine poisoning. e-Cigarettes are also popular among youth,​ with rates of e-cigarette use surpassing those of cigarette use in this population. Youth e-cigarette use is associated with increased risk of subsequent cigarette and marijuana use. Screening for e-cigarette use in youth and adults,​ including pregnant women,​ in conjunction with screening for tobacco use,​ is advised. Education and interventions to prevent e-cigarette use should be provided to all youth. Youth should be counseled to stop using nicotine/tobacco products,​ including e-cigarettes. Although the impact of e-cigarette use in pregnancy is unknown,​ nicotine is a teratogen; thus,​ pregnant women should be counseled to abstain from using all nicotine/tobacco products. |
| 128 | Sarah Gentry,​ Nita G Forouhi,​ Caitlin Notley (2019) Are Electronic Cigarettes an Effective Aid to Smoking Cessation or Reduction Among Vulnerable Groups? A Systematic Review of Quantitative and Qualitative Evidence.. Nicotine & tobacco research : official journal of the Society for Research on Nicotine and Tobacco; 21.  Keywords:   Abstract: INTRODUCTION: Smoking prevalence remains high in some vulnerable groups,​ including those who misuse substances,​ have a mental illness,​ are homeless,​ or are involved with the criminal justice system. E-cigarette use is increasing and may support smoking cessation/reduction.,​ METHODS: Systematic review of quantitative and qualitative data on the effectiveness of e-cigarettes for smoking cessation/reduction among vulnerable groups. Databases searched were MEDLINE,​ EMBASE,​ PsychINFO,​ CINAHL,​ ASSIA,​ ProQuest Dissertations and Theses,​ and Open Grey. Narrative synthesis of quantitative data and thematic synthesis of qualitative data.,​ RESULTS: 2628 records and 46 full texts were screened; 9 studies were identified for inclusion. Due to low quality of evidence,​ it is uncertain whether e-cigarettes are effective for smoking cessation in vulnerable populations. A moderate quality study suggested that e-cigarettes were as effective as nicotine replacement therapy. Four studies suggested significant smoking reduction; however,​ three were uncontrolled and had sample sizes below 30. A prospective cohort study found no differences between e-cigarette users and nonusers. No significant adverse events and minimal side effects were identified. Qualitative thematic synthesis revealed barriers and facilitators associated with each component of the COM-B (capability,​ opportunity,​ motivation,​ and behavior) model,​ including practical barriers; perceptions of effectiveness for cessation/reduction; design features contributing to automatic and reflective motivation; smoking bans facilitating practical opportunity; and social connectedness increasing social opportunity.,​ CONCLUSION: Further research is needed to identify the most appropriate device types for practicality and safety,​ level of support required in e-cigarette interventions,​ and to compare e-cigarettes with current best practice smoking cessation support among vulnerable groups.,​ IMPLICATIONS: Smoking prevalence among people with mental illness,​ substance misuse,​ homelessness,​ or criminal justice system involvement remains high. E-cigarettes could support cessation. This systematic review found limited quantitative evidence assessing effectiveness. No serious adverse events were identified. Qualitative thematic synthesis revealed barriers and facilitators mapping to each component of the COM-B (capability,​ opportunity,​ motivation,​ and behavior) model,​ including practical barriers; perceived effectiveness; design features contributing to automatic and reflective motivation; smoking bans facilitating practical opportunity; and social connectedness increasing social opportunity. Further research should consider appropriate devices for practicality and safety,​ concurrent support,​ and comparison with best practice smoking cessation support. Copyright © The Author(s) 2018. Published by Oxford University Press on behalf of the Society for Research on Nicotine and Tobacco. All rights reserved. For permissions,​ please e-mail: journals.permissions@oup.com. |
| 133 | Robert West,​ Kathryn Coyle,​ Lesley Owen,​ et al (2018) Estimates of effectiveness and reach for 'return on investment' modelling of smoking cessation interventions using data from England.. Addiction (Abingdon,​ England); 113 Suppl 1.  Keywords:   Abstract: BACKGROUND AND AIMS: Estimating 'return on investment' (ROI) from smoking cessation interventions requires reach and effectiveness parameters for interventions for use in economic models such as the EQUIPT ROI tool (http:roi.equipt.eu). This paper describes the derivation of these parameter estimates for England that can be adapted to create ROI models for use by other countries.,​ METHODS: Estimates were derived for interventions in terms of their reach and effectiveness in: (1) promoting quit attempts and (2) improving the success of quit attempts (abstinence for at least 12 months). The sources were systematic reviews of efficacy supplemented by individual effectiveness evaluations and national surveys.,​ FINDINGS: Quit attempt rates were estimated to be increased by the following percentages (with reach in parentheses): 20% by tax increases raising the cost of smoking 5% above the cost of living index (100%); 10% by enforced comprehensive indoor public smoking bans (100%); 3% by mass media campaigns achieving 400 gross rating points (100%); 40% by brief opportunistic physician advice (21%); and 110% by use of a licensed nicotine product to reduce cigarette consumption (12%). Quit success rates were estimated to be increased by the following ratios: 60% by single-form nicotine replacement therapy (NRT) (5%); 114% by NRT patch plus a faster-acting NRT (2%);124% by prescribed varenicline (5%); 60% by bupropion (1%); 100% by nortriptyline (0%),​ 10) 298% by cytisine (0%); 40% by individual face-to-face behavioural support (2%); 37% by telephone support (0.5%); 88% by group behavioural support (1%); 63% by text messaging (0.5%); and 19% by printed self-help materials (1%). There was insufficient evidence to obtain reliable,​ country-specific estimates for interventions such as websites,​ smartphone applications and e-cigarettes.,​ CONCLUSIONS: Tax increases,​ indoor smoking bans,​ brief opportunistic physician advice and use of nicotine replacement therapy (NRT) for smoking reduction can all increase population quit attempt rates. Quit success rates can be increased by provision of NRT,​ varenicline,​ bupropion,​ nortriptyline,​ cytisine and behavioural support delivered through a variety of modalities. Parameter estimates for the effectiveness and reach of these interventions can contribute to return on investment estimates in support of national or regional policy decisions. Copyright © 2017 The Authors. Addiction published by John Wiley & Sons Ltd on behalf of Society for the Study of Addiction. |
| 140 | Jamie Hartmann-Boyce,​ Hayden McRobbie,​ Chris Bullen,​ et al (2016) Electronic cigarettes for smoking cessation.. The Cochrane database of systematic reviews; 9.  Keywords:   Abstract: BACKGROUND: Electronic cigarettes (ECs) are electronic devices that heat a liquid into an aerosol for inhalation. The liquid usually comprises propylene glycol and glycerol,​ with or without nicotine and flavours,​ and stored in disposable or refillable cartridges or a reservoir. Since ECs appeared on the market in 2006 there has been a steady growth in sales. Smokers report using ECs to reduce risks of smoking,​ but some healthcare organizations,​ tobacco control advocacy groups and policy makers have been reluctant to encourage smokers to switch to ECs,​ citing lack of evidence of efficacy and safety. Smokers,​ healthcare providers and regulators are interested to know if these devices can help smokers quit and if they are safe to use for this purpose. This review is an update of a review first published in 2014.,​ OBJECTIVES: To evaluate the safety and effect of using ECs to help people who smoke achieve long-term smoking abstinence.,​ SEARCH METHODS: We searched the Cochrane Tobacco Addiction Group's Specialized Register,​ the Cochrane Central Register of Controlled Trials (CENTRAL),​ MEDLINE,​ Embase,​ and PsycINFO for relevant records from 2004 to January 2016,​ together with reference checking and contact with study authors.,​ SELECTION CRITERIA: We included randomized controlled trials (RCTs) in which current smokers (motivated or unmotivated to quit) were randomized to EC or a control condition,​ and which measured abstinence rates at six months or longer. As the field of EC research is new,​ we also included cohort follow-up studies with at least six months follow-up. We included randomized cross-over trials,​ RCTs and cohort follow-up studies that included at least one week of EC use for assessment of adverse events (AEs).,​ DATA COLLECTION AND ANALYSIS: We followed standard Cochrane methods for screening and data extraction. Our main outcome measure was abstinence from smoking after at least six months follow-up,​ and we used the most rigorous definition available (continuous,​ biochemically validated,​ longest follow-up). We used a fixed-effect Mantel-Haenszel model to calculate the risk ratio (RR) with a 95% confidence interval (CI) for each study,​ and where appropriate we pooled data from these studies in meta-analyses.,​ MAIN RESULTS: Our searches identified over 1700 records,​ from which we include 24 completed studies (three RCTs,​ two of which were eligible for our cessation meta-analysis,​ and 21 cohort studies). Eleven of these studies are new for this version of the review. We identified 27 ongoing studies. Two RCTs compared EC with placebo (non-nicotine) EC,​ with a combined sample size of 662 participants. One trial included minimal telephone support and one recruited smokers not intending to quit,​ and both used early EC models with low nicotine content and poor battery life. We judged the RCTs to be at low risk of bias,​ but under the GRADE system we rated the overall quality of the evidence for our outcomes as 'low' or 'very low',​ because of imprecision due to the small number of trials. A 'low' grade means that further research is very likely to have an important impact on our confidence in the estimate of effect and is likely to change the estimate. A 'very low' grade means we are very uncertain about the estimate. Participants using an EC were more likely to have abstained from smoking for at least six months compared with participants using placebo EC (RR 2.29,​ 95% CI 1.05 to 4.96; placebo 4% versus EC 9%; 2 studies; 662 participants. GRADE: low). The one study that compared EC to nicotine patch found no significant difference in six-month abstinence rates,​ but the confidence intervals do not rule out a clinically important difference (RR 1.26,​ 95% CI 0.68 to 2.34; 584 participants. GRADE: very low).Of the included studies,​ none reported serious adverse events considered related to EC use. The most frequently reported AEs were mouth and throat irritation,​ most commonly dissipating over time. One RCT provided data on the proportion of participants experiencing any adverse events. The proportion of participants in the study arms experiencing adverse events was similar (ECs vs placebo EC: RR 0.97,​ 95% CI 0.71 to 1.34 (298 participants); ECs vs patch: RR 0.99,​ 95% CI 0.81 to 1.22 (456 participants)). The second RCT reported no statistically significant difference in the frequency of AEs at three- or 12-month follow-up between the EC and placebo EC groups,​ and showed that in all groups the frequency of AEs (with the exception of throat irritation) decreased significantly over time.,​ AUTHORS' CONCLUSIONS: There is evidence from two trials that ECs help smokers to stop smoking in the long term compared with placebo ECs. However,​ the small number of trials,​ low event rates and wide confidence intervals around the estimates mean that our confidence in the result is rated 'low' by GRADE standards. The lack of difference between the effect of ECs compared with nicotine patches found in one trial is uncertain for similar reasons. None of the included studies (short- to mid-term,​ up to two years) detected serious adverse events considered possibly related to EC use. The most commonly reported adverse effects were irritation of the mouth and throat. The long-term safety of ECs is unknown. In this update,​ we found a further 15 ongoing RCTs which appear eligible for this review. |
| 148 | Caroline Franck,​ Kristian B Filion,​ Mark J Eisenberg (2018) Smoking Cessation in Patients With Acute Coronary Syndrome.. The American journal of cardiology; 121.  Keywords: *Acute Coronary Syndrome/th [Therapy],​Behavior Therapy,​*Bupropion/tu [Therapeutic Use],​*Cardiac Rehabilitation,​*Counseling,​Electronic Nicotine Delivery Systems,​Humans,​Motivation,​*Smoking Cessation/mt [Methods],​*Smoking Cessation Agents/tu [Therapeutic Use],​*Tobacco Use Cessation Devices,​*Varenicline/tu [Therapeutic Use]  Abstract: Over 30% of the nearly 1 million North Americans hospitalized annually with an acute coronary syndrome (ACS) are smokers. Despite a substantially increased risk of morbidity and mortality,​ 2/3 of patients who quit smoking after ACS return to smoking within 1 year. To summarize the evidence of smoking cessation in patients hospitalized after ACS,​ we systematically reviewed all randomized controlled trials of pharmacologic and behavioral smoking cessation therapies in patients with ACS. In addition,​ we reviewed the clinical considerations surrounding the use of smoking cessation therapies,​ including their broad mechanisms of action and possible alternative treatments,​ including cardiac rehabilitation programs and electronic cigarettes. A total of 7 randomized controlled trials met our inclusion criteria (4 pharmacotherapies and 3 behavioral therapies). In pharmacologic trials,​ only varenicline increased point prevalence abstinence at 12 months. Behavioral interventions produced significantly improved abstinence rates at 6 and 12 months. However,​ these studies had substantial limitations affecting their generalizability. Overall,​ currently available smoking cessation therapies are limited in their efficacy in patients hospitalized after ACS. Because of the relative scarcity of data and the urgency of establishing clinical guidelines,​ there is a critical need to continue examining the efficacy and safety of smoking cessation interventions in patients hospitalized after ACS. Copyright © 2018 Elsevier Inc. All rights reserved. |
| 154 | Tim Coleman,​ Catherine Chamberlain,​ Mary-Ann Davey,​ et al (2015) Pharmacological interventions for promoting smoking cessation during pregnancy.. The Cochrane database of systematic reviews; .  Keywords: Bupropion/tu [Therapeutic Use],​Female,​Humans,​Nicotinic Agonists/tu [Therapeutic Use],​Pregnancy,​*Pregnancy Complications/dt [Drug Therapy],​Pregnancy Outcome,​Randomized Controlled Trials as Topic,​*Smoking Cessation/mt [Methods],​*Tobacco Use Cessation Devices,​Varenicline/tu [Therapeutic Use]  Abstract: BACKGROUND: Smoking in pregnancy is a public health problem. When used by non-pregnant smokers,​ pharmacotherapies (nicotine replacement therapy (NRT),​ bupropion and varenicline) are effective for smoking cessation,​ however,​ their efficacy and safety in pregnancy remains unknown. Electronic Nicotine Delivery Systems (ENDS),​ or e-cigarettes,​ are becoming widely used but their efficacy and safety when used for smoking cessation in pregnancy are also unknown.,​ OBJECTIVES: To determine the efficacy and safety of smoking cessation pharmacotherapies (including NRT,​ varenicline and bupropion),​ other medications,​ or ENDS when used for smoking cessation in pregnancy.,​ SEARCH METHODS: We searched the Pregnancy and Childbirth Group's Trials Register (11 July 2015),​ checked references of retrieved studies,​ and contacted authors.,​ SELECTION CRITERIA: Randomised controlled trials (RCTs) conducted in pregnant women with designs that permit the independent effects of any type of pharmacotherapy or ENDS on smoking cessation to be ascertained were eligible for inclusion.The following RCT designs are included.Placebo-RCTs: any form of NRT,​ other pharmacotherapy,​ or ENDS,​ with or without behavioural support/cognitive behaviour therapy (CBT),​ or brief advice,​ compared with an identical placebo and behavioural support of similar intensity.RCTs providing a comparison between i) any form of NRT,​ other pharmacotherapy,​ or ENDS added to behavioural support/CBT,​ or brief advice and ii) behavioural support of similar (ideally identical) intensity.Parallel- or cluster-randomised trials were eligible for inclusion. Quasi-randomised,​ cross-over and within-participant designs were not,​ due to the potential biases associated with these designs.,​ DATA COLLECTION AND ANALYSIS: Two review authors independently assessed trials for inclusion and risk of bias and also independently extracted data and cross checked individual outcomes of this process to ensure accuracy. The primary efficacy outcome was smoking cessation in later pregnancy (in all but one trial,​ at or around delivery); safety was assessed by 11 outcomes (principally birth outcomes) that indicated neonatal and infant well-being; and we also collated data on adherence with trial treatments.,​ MAIN RESULTS: This review includes a total of nine trials which enrolled 2210 pregnant smokers: eight trials of NRT and one trial of bupropion as adjuncts to behavioural support/CBT. The risk of bias was generally low across trials with virtually all domains of the 'Risk of bias' assessment tool being satisfied for the majority of studies. We found no trials investigating varenicline or ENDS. Compared to placebo and non-placebo controls,​ there was a difference in smoking rates observed in later pregnancy favouring use of NRT (risk ratio (RR) 1.41,​ 95% confidence interval (CI) 1.03 to 1.93,​ eight studies,​ 2199 women). However,​ subgroup analysis of placebo-RCTs provided a lower RR in favour of NRT (RR 1.28,​ 95% CI 0.99 to 1.66,​ five studies,​ 1926 women),​ whereas within the two non-placebo RCTs there was a strong positive effect of NRT,​ (RR 8.51,​ 95% CI 2.05 to 35.28,​ three studies,​ 273 women; P value for random-effects subgroup interaction test = 0.01). There were no differences between NRT and control groups in rates of miscarriage,​ stillbirth,​ premature birth,​ birthweight,​ low birthweight,​ admissions to neonatal intensive care,​ caesarean section,​ congenital abnormalities or neonatal death. Compared to placebo group infants,​ at two years of age,​ infants born to women who had been randomised to NRT had higher rates of 'survival without developmental impairment' (one trial). Generally,​ adherence with trial NRT regimens was low. Non-serious side effects observed with NRT included headache,​ nausea and local reactions (e.g. skin irritation from patches or foul taste from gum),​ but these data could not be pooled.,​ AUTHORS' CONCLUSIONS: NRT used in pregnancy for smoking cessation increases smoking cessation rates measured in late pregnancy by approximately 40%. There is evidence,​ suggesting that when potentially-biased,​ non-placebo RCTs are excluded from analyses,​ NRT is no more effective than placebo. There is no evidence that NRT used for smoking cessation in pregnancy has either positive or negative impacts on birth outcomes. However,​ evidence from the only trial to have followed up infants after birth,​ suggests use of NRT promotes healthy developmental outcomes in infants. Further research evidence on NRT efficacy and safety is needed,​ ideally from placebo-controlled RCTs which achieve higher adherence rates and which monitor infants' outcomes into childhood. Accruing data suggests that it would be ethical for future RCTs to investigate higher doses of NRT than those tested in the included studies. |
| 158 | Regina El Dib,​ Erica A Suzumura,​ Elie A Akl,​ et al (2017) Electronic nicotine delivery systems and/or electronic non-nicotine delivery systems for tobacco smoking cessation or reduction: a systematic review and meta-analysis.. BMJ open; 7.  Keywords: Behavior Therapy,​*Electronic Nicotine Delivery Systems,​Humans,​Nicotinic Agonists/ad [Administration & Dosage],​Randomized Controlled Trials as Topic,​*Smoking Cessation/mt [Methods],​*Tobacco Smoking/th [Therapy],​Tobacco Use Cessation Devices  Abstract: OBJECTIVE: A systematic review and meta-analysis to investigate the impact of electronic nicotine delivery systems (ENDS) and/or electronic non-nicotine delivery systems (ENNDS) versus no smoking cessation aid,​ or alternative smoking cessation aids,​ in cigarette smokers on long-term tobacco use.,​ DATA SOURCES: Searches of MEDLINE,​ EMBASE,​ PsycInfo,​ CINAHL,​ CENTRAL and Web of Science up to December 2015.,​ STUDY SELECTION: Randomised controlled trials (RCTs) and prospective cohort studies.,​ DATA EXTRACTION: Three pairs of reviewers independently screened potentially eligible articles,​ extracted data from included studies on populations,​ interventions and outcomes and assessed their risk of bias. We used the Grading of Recommendations Assessment,​ Development and Evaluation approach to rate overall certainty of the evidence by outcome.,​ DATA SYNTHESIS: Three randomised trials including 1007 participants and nine cohorts including 13 115 participants proved eligible. Results provided by only two RCTs suggest a possible increase in tobacco smoking cessation with ENDS in comparison with ENNDS (RR 2.03,​ 95% CI 0.94 to 4.38; p=0.07; I2=0%,​ risk difference (RD) 64/1000 over 6 to 12 months,​ low-certainty evidence). Results from cohort studies suggested a possible reduction in quit rates with use of ENDS compared with no use of ENDS (OR 0.74,​ 95% CI 0.55 to 1.00; p=0.051; I2=56%,​ very low certainty).,​ CONCLUSIONS: There is very limited evidence regarding the impact of ENDS or ENNDS on tobacco smoking cessation,​ reduction or adverse effects: data from RCTs are of low certainty and observational studies of very low certainty. The limitations of the cohort studies led us to a rating of very low-certainty evidence from which no credible inferences can be drawn. Lack of usefulness with regard to address the question of e-cigarettes' efficacy on smoking reduction and cessation was largely due to poor reporting. This review underlines the need to conduct well-designed trials measuring biochemically validated outcomes and adverse effects. Copyright Published by the BMJ Publishing Group Limited. For permission to use (where not already granted under a licence) please go to http:www.bmj.com/company/products-services/rights-and-licensing/. |
| 160 | Samir Soneji,​ Jessica L Barrington-Trimis,​ Thomas A Wills,​ et al (2017) Association Between Initial Use of e-Cigarettes and Subsequent Cigarette Smoking Among Adolescents and Young Adults: A Systematic Review and Meta-analysis.. JAMA pediatrics; 171.  Keywords: Adolescent,​*Adolescent Behavior,​Disease Progression,​Electronic Nicotine Delivery Systems/px [Psychology],​*Electronic Nicotine Delivery Systems/sn [Statistics & Numerical Data],​Female,​Humans,​Male,​Risk Factors,​*Smoking/ep [Epidemiology],​Smoking/px [Psychology],​Smoking Cessation/sn [Statistics & Numerical Data],​*Tobacco Use Disorder/ep [Epidemiology],​Tobacco Use Disorder/px [Psychology],​Young Adult  Abstract: Importance: The public health implications of e-cigarettes depend,​ in part,​ on whether e-cigarette use affects the risk of cigarette smoking.,​ Objective: To perform a systematic review and meta-analysis of longitudinal studies that assessed initial use of e-cigarettes and subsequent cigarette smoking.,​ Data Sources: PubMed,​ EMBASE,​ Cochrane Library,​ Web of Science,​ the 2016 Society for Research on Nicotine and Tobacco 22nd Annual Meeting abstracts,​ the 2016 Society of Behavioral Medicine 37th Annual Meeting & Scientific Sessions abstracts,​ and the 2016 National Institutes of Health Tobacco Regulatory Science Program Conference were searched between February 7 and February 17,​ 2017. The search included indexed terms and text words to capture concepts associated with e-cigarettes and traditional cigarettes in articles published from database inception to the date of the search.,​ Study Selection: Longitudinal studies reporting odds ratios for cigarette smoking initiation associated with ever use of e-cigarettes or past 30-day cigarette smoking associated with past 30-day e-cigarette use. Searches yielded 6959 unique studies,​ of which 9 met inclusion criteria (comprising 17389 adolescents and young adults).,​ Data Extraction and Synthesis: Study quality and risk of bias were assessed using the Newcastle-Ottawa Scale and the Risk of Bias in Non-randomized Studies of Interventions tool,​ respectively. Data and estimates were pooled using random-effects meta-analysis.,​ Main Outcomes and Measures: Among baseline never cigarette smokers,​ cigarette smoking initiation between baseline and follow-up. Among baseline non-past 30-day cigarette smokers who were past 30-day e-cigarette users,​ past 30-day cigarette smoking at follow-up.,​ Results: Among 17389 adolescents and young adults,​ the ages ranged between 14 and 30 years at baseline,​ and 56.0% were female. The pooled probabilities of cigarette smoking initiation were 30.4% for baseline ever e-cigarette users and 7.9% for baseline never e-cigarette users. The pooled probabilities of past 30-day cigarette smoking at follow-up were 21.5% for baseline past 30-day e-cigarette users and 4.6% for baseline non-past 30-day e-cigarette users. Adjusting for known demographic,​ psychosocial,​ and behavioral risk factors for cigarette smoking,​ the pooled odds ratio for subsequent cigarette smoking initiation was 3.62 (95% CI,​ 2.42-5.41) for ever vs never e-cigarette users,​ and the pooled odds ratio for past 30-day cigarette smoking at follow-up was 4.28 (95% CI,​ 2.52-7.27) for past 30-day e-cigarette vs non-past 30-day e-cigarette users at baseline. A moderate level of heterogeneity was observed among studies (I2 = 60.1%).,​ Conclusions and Relevance: e-Cigarette use was associated with greater risk for subsequent cigarette smoking initiation and past 30-day cigarette smoking. Strong e-cigarette regulation could potentially curb use among youth and possibly limit the future population-level burden of cigarette smoking. |
| 161 | Catherine Chamberlain,​ Susan Perlen,​ Sue Brennan,​ et al (2017) Evidence for a comprehensive approach to Aboriginal tobacco control to maintain the decline in smoking: an overview of reviews among Indigenous peoples.. Systematic reviews; 6.  Keywords: Choice Behavior,​Humans,​*Oceanic Ancestry Group,​Prevalence,​*Smoking/ep [Epidemiology],​*Smoking/eh [Ethnology],​*Smoking Cessation/eh [Ethnology]  Abstract: BACKGROUND: Tobacco smoking is a leading cause of disease and premature mortality among Aboriginal and Torres Strait Islander (Indigenous) Australians. While the daily smoking prevalence among Indigenous Australians has declined significantly from 49% in 2001,​ it remains about three times higher than that of non-Indigenous Australians (39 and 14%,​ respectively,​ for age >=15 years in 2014-15). This overview of systematic reviews aimed to synthesise evidence about reducing tobacco consumption among Indigenous peoples using a comprehensive framework for Indigenous tobacco control in Australia comprised of the National Tobacco Strategy (NTS) and National Aboriginal and Torres Strait Islander Health Plan (NATSIHP) principles and priorities.,​ METHODS: MEDLINE,​ EMBASE,​ systematic review and Indigenous health databases were searched (2000 to Jan 2016) for reviews examining the effects of tobacco control interventions among Indigenous peoples. Two reviewers independently screened reviews,​ extracted data,​ and assessed review quality using Assessing the Methodological Quality of Systematic Reviews. Data were synthesised narratively by framework domain. Reporting followed the PRISMA statement.,​ RESULTS: Twenty-one reviews of varying quality were included. There was generally limited Indigenous-specific evidence of effective interventions for reducing smoking; however,​ many reviewers recommended multifaceted interventions which incorporate Indigenous leadership,​ partnership and engagement and cultural tailoring. Under the NTS priority areas,​ reviewers reported evidence for brief smoking cessation interventions and pharmacological support,​ mass media campaigns (on knowledge and attitudes) and reducing affordability and regulation of tobacco sales. Aspects of intervention implementation related to the NATSIHP domains were less well described and evidence was limited; however,​ reviewers suggested that cultural tailoring,​ holistic approaches and building workforce capacity were important strategies to address barriers. There was limited evidence regarding social media and mobile applications,​ for Indigenous youth,​ pregnant women and prisoners,​ and no evidence regarding interventions to protect communities from industry interference,​ the use of electronic cigarettes,​ interventions for people experiencing mental illness,​ juvenile justice,​ linguistic diversity or 'pubs,​ clubs and restaurants'.,​ CONCLUSIONS: There is limited Indigenous-specific evidence for most tobacco interventions. A 'comprehensive approach' incorporating NTS and NATSIHP Principles and Priorities of partnership and engagement,​ evidence from other settings,​ programme logic and responsive evaluation plans may improve intervention acceptability,​ effectiveness and implementation and mitigate risks of adapting tobacco evidence for Indigenous Australians. |
| 163 | Hayden McRobbie,​ Chris Bullen,​ Jamie Hartmann-Boyce,​ et al (2014) Electronic cigarettes for smoking cessation and reduction.. The Cochrane database of systematic reviews; .  Keywords: Cohort Studies,​Electronic Nicotine Delivery Systems/ae [Adverse Effects],​Electronic Nicotine Delivery Systems/is [Instrumentation],​*Electronic Nicotine Delivery Systems,​Humans,​Middle Aged,​Nicotine/ad [Administration & Dosage],​Nicotinic Agonists/ad [Administration & Dosage],​Publication Bias,​Randomized Controlled Trials as Topic,​Smoking/ep [Epidemiology],​*Smoking Cessation/mt [Methods],​*Smoking Prevention,​Tobacco Use Cessation Devices  Abstract: BACKGROUND: Electronic cigarettes (ECs) are electronic devices that heat a liquid - usually comprising propylene glycol and glycerol,​ with or without nicotine and flavours,​ stored in disposable or refillable cartridges or a reservoir - into an aerosol for inhalation. Since ECs appeared on the market in 2006 there has been a steady growth in sales. Smokers report using ECs to reduce risks of smoking,​ but some healthcare organisations have been reluctant to encourage smokers to switch to ECs,​ citing lack of evidence of efficacy and safety. Smokers,​ healthcare providers and regulators are interested to know if these devices can reduce the harms associated with smoking. In particular,​ healthcare providers have an urgent need to know what advice they should give to smokers enquiring about ECs.,​ OBJECTIVES: To examine the efficacy of ECs in helping people who smoke to achieve long-term abstinence; to examine the efficacy of ECs in helping people reduce cigarette consumption by at least 50% of baseline levels; and to assess the occurrence of adverse events associated with EC use.,​ SEARCH METHODS: We searched the Cochrane Tobacco Addiction Groups Trials Register,​ the Cochrane Central Register of Controlled Trials (CENTRAL),​ MEDLINE,​ Embase,​ and two other databases for relevant records from 2004 to July 2014,​ together with reference checking and contact with study authors.,​ SELECTION CRITERIA: We included randomized controlled trials (RCTs) in which current smokers (motivated or unmotivated to quit) were randomized to EC or a control condition,​ and which measured abstinence rates or changes in cigarette consumption at six months or longer. As the field of EC research is new,​ we also included cohort follow-up studies with at least six months follow-up. We included randomized cross-over trials and cohort follow-up studies that included at least one week of EC use for assessment of adverse events.,​ DATA COLLECTION AND ANALYSIS: One review author extracted data from the included studies and another checked them. Our main outcome measure was abstinence from smoking after at least six months follow-up,​ and we used the most rigorous definition available (continuous,​ biochemically validated,​ longest follow-up). For reduction we used a dichotomous approach (no change/reduction < 50% versus reduction by 50% or more of baseline cigarette consumption). We used a fixed-effect Mantel-Haenszel model to calculate the risk ratio (RR) with a 95% confidence interval (CI) for each study,​ and where appropriate we pooled data from these studies in meta-analyses.,​ MAIN RESULTS: Our search identified almost 600 records,​ from which we include 29 representing 13 completed studies (two RCTs,​ 11 cohort). We identified nine ongoing trials. Two RCTs compared EC with placebo (non-nicotine) EC,​ with a combined sample size of 662 participants. One trial included minimal telephone support and one recruited smokers not intending to quit,​ and both used early EC models with low nicotine content. We judged the RCTs to be at low risk of bias,​ but under the GRADE system the overall quality of the evidence for our outcomes was rated 'low' or 'very low' because of imprecision due to the small number of trials. A 'low' grade means that further research is very likely to have an important impact on our confidence in the estimate of effect and is likely to change the estimate. A 'very low' grade means we are very uncertain about the estimate. Participants using an EC were more likely to have abstained from smoking for at least six months compared with participants using placebo EC (RR 2.29,​ 95% CI 1.05 to 4.96; placebo 4% versus EC 9%; 2 studies; GRADE: low). The one study that compared EC to nicotine patch found no significant difference in six-month abstinence rates,​ but the confidence intervals do not rule out a clinically important difference (RR 1.26,​ 95% CI: 0.68 to 2.34; GRADE: very low). A higher number of people were able to reduce cigarette consumption by at least half with ECs compared with placebo ECs (RR 1.31,​ 95% CI 1.02 to 1.68,​ 2 studies; placebo: 27% versus EC: 36%; GRADE: low) and compared with patch (RR 1.41,​ 95% CI 1.20 to 1.67,​ 1 study; patch: 44% versus EC: 61%; GRADE: very low). Unlike smoking cessation outcomes,​ reduction results were not biochemically verified.None of the RCTs or cohort studies reported any serious adverse events (SAEs) that were considered to be plausibly related to EC use. One RCT provided data on the proportion of participants experiencing any adverse events. Although the proportion of participants in the study arms experiencing adverse events was similar,​ the confidence intervals are wide (ECs vs placebo EC RR 0.97,​ 95% CI 0.71 to 1.34; ECs vs patch RR 0.99,​ 95% CI 0.81 to 1.22). The other RCT reported no statistically significant difference in the frequency of AEs at three- or 12-month follow-up between the EC and placebo EC groups,​ and showed that in all groups the frequency of AEs (with the exception of throat irritation) decreased significantly over time.,​ AUTHORS' CONCLUSIONS: There is evidence from two trials that ECs help smokers to stop smoking long-term compared with placebo ECs. However,​ the small number of trials,​ low event rates and wide confidence intervals around the estimates mean that our confidence in the result is rated 'low' by GRADE standards. The lack of difference between the effect of ECs compared with nicotine patches found in one trial is uncertain for similar reasons. ECs appear to help smokers unable to stop smoking altogether to reduce their cigarette consumption when compared with placebo ECs and nicotine patches,​ but the above limitations also affect certainty in this finding. In addition,​ lack of biochemical assessment of the actual reduction in smoke intake further limits this evidence. No evidence emerged that short-term EC use is associated with health risk. |
| 166 | Xing Liu,​ Wan Lu,​ Sheng Liao,​ et al (2018) Efficiency and adverse events of electronic cigarettes: A systematic review and meta-analysis (PRISMA-compliant article).. Medicine; 97.  Keywords: *Electronic Nicotine Delivery Systems/sn [Statistics & Numerical Data],​Humans,​Smoking Cessation/mt [Methods],​Smoking Prevention/mt [Methods],​Treatment Outcome  Abstract: BACKGROUND: Electronic cigarettes (e-cigarettes) are a prevalent smoking cessation aid worldwide; however,​ a consensus regarding their efficacy and safety has yet to be reached.,​ METHODS: We conducted a systematic review of the literature from related studies written in English or Chinese and published between January 1,​ 2003,​ and July 30,​ 2017. Eligible studies reporting the number of smokers who reduced or quit smoking and suffered from adverse events after e-cigarette use were selected according to predefined criteria; pertinent data were then extracted for a meta-analysis.,​ RESULTS: Our search produced 198 articles; of these publications,​ 14 including 35,​665 participants were analyzed. The pooled efficacy rate of e-cigarettes ranged from 48.3% to 58.7% for smoking reduction and from 13.2% to 22.9% for smoking cessation. The pooled rate of adverse events associated with e-cigarettes ranged from 49.1% to 51.6% based on 11 studies including 16,​406 participants. The most prevalent adverse events were mouth or throat irritation,​ anxiety,​ depressed mood,​ nausea,​ and insomnia. No significant differences in overall CO2 exhalation (eCO) levels were observed after e-cigarette use according to the data from 5 studies.,​ CONCLUSION: Our findings suggest that e-cigarettes are moderately effective with regard to smoking reduction and smoking cessation. eCO levels are unreliable for evaluating the efficacy of e-cigarettes. E-cigarette related adverse events frequently occur,​ especially due to high-dose nicotine-containing cartridges. |
| 167 | Samane Zare,​ Mehdi Nemati,​ Yuqing Zheng (2018) A systematic review of consumer preference for e-cigarette attributes: Flavor,​ nicotine strength,​ and type.. PloS one; 13.  Keywords: Age Factors,​*Consumer Behavior,​Electronic Nicotine Delivery Systems/is [Instrumentation],​Electronic Nicotine Delivery Systems/mt [Methods],​*Electronic Nicotine Delivery Systems,​Flavoring Agents/an [Analysis],​Humans,​Nicotine/an [Analysis],​Sex Factors,​Sweetening Agents/an [Analysis],​Taste Perception  Abstract: OBJECTIVE: Systematic review of research examining consumer preference for the main electronic cigarette (e-cigarette) attributes namely flavor,​ nicotine strength,​ and type.,​ METHOD: A systematic search of peer-reviewed articles resulted in a pool of 12,​933 articles. We included only articles that meet all the selection criteria: (1) peer-reviewed,​ (2) written in English,​ and (3) addressed consumer preference for one or more of the e-cigarette attributes including flavor,​ strength,​ and type.,​ RESULTS: 66 articles met the inclusion criteria for this review. Consumers preferred flavored e-cigarettes,​ and such preference varied with age groups and smoking status. We also found that several flavors were associated with decreased harm perception while tobacco flavor was associated with increased harm perception. In addition,​ some flavor chemicals and sweeteners used in e-cigarettes could be of toxicological concern. Finally,​ consumer preference for nicotine strength and types depended on smoking status,​ e-cigarette use history,​ and gender.,​ CONCLUSION: Adolescents could consider flavor the most important factor trying e-cigarettes and were more likely to initiate vaping through flavored e-cigarettes. Young adults overall preferred sweet,​ menthol,​ and cherry flavors,​ while non-smokers in particular preferred coffee and menthol flavors. Adults in general also preferred sweet flavors (though smokers like tobacco flavor the most) and disliked flavors that elicit bitterness or harshness. In terms of whether flavored e-cigarettes assisted quitting smoking,​ we found inconclusive evidence. E-cigarette users likely initiated use with a cigarette like product and transitioned to an advanced system with more features. Non-smokers and inexperienced e-cigarettes users tended to prefer no nicotine or low nicotine e-cigarettes while smokers and experienced e-cigarettes users preferred medium and high nicotine e-cigarettes. Weak evidence exists regarding a positive interaction between menthol flavor and nicotine strength. |
| 176 | Yanina Zborovskaya (2017) E-Cigarettes and Smoking Cessation: A Primer for Oncology Clinicians.. Clinical journal of oncology nursing; 21.  Keywords: *Electronic Nicotine Delivery Systems/ae [Adverse Effects],​Electronic Nicotine Delivery Systems/sn [Statistics & Numerical Data],​Female,​Head and Neck Neoplasms/et [Etiology],​Head and Neck Neoplasms/pc [Prevention & Control],​Humans,​Lung Neoplasms/et [Etiology],​Lung Neoplasms/pc [Prevention & Control],​Male,​Medical Oncology/og [Organization & Administration],​Needs Assessment,​*Physician's Role,​Practice Patterns,​ Physicians'/og [Organization & Administration],​*Primary Prevention/og [Organization & Administration],​Public Health,​Smoking/ae [Adverse Effects],​Smoking/ep [Epidemiology],​*Smoking Cessation/mt [Methods],​United States  Abstract: BACKGROUND: Electronic cigarettes (e-cigarettes) are a psychosocial phenomenon of the 21st century with serious implications to public and individual health. The significant increase of their popularity and use has raised concerns in the healthcare community regarding their potential benefits and harm,​ particularly their use as a smoking cessation methodology.,​ OBJECTIVES: Current knowledge is presented about e-cigarettes,​ including the clinical implications of their use,​ and associated controversies are analyzed. In addition,​ practice recommendations and communication guidance are provided.,​ METHODS: An integrative review was performed.,​ FINDINGS: The long-term effect of e-cigarettes on individual and public health is unknown. Evidence from short-term studies,​ although limited,​ shows that e-cigarette use is less harmful than smoking. However,​ the evidence does not support the use of e-cigarettes as a smoking cessation methodology. Such recommendation remains a judgment call for the clinician based on each individual case. |
| 179 | Allison M Glasser,​ Lauren Collins,​ Jennifer L Pearson,​ et al (2017) Overview of Electronic Nicotine Delivery Systems: A Systematic Review.. American journal of preventive medicine; 52.  Keywords: Biomarkers/an [Analysis],​Cognition/de [Drug Effects],​Commerce,​*Electronic Nicotine Delivery Systems,​Humans,​Marketing,​*Nicotine/ae [Adverse Effects],​Smoking Cessation/ec [Economics],​*Smoking Cessation/mt [Methods],​Smoking Prevention/ec [Economics],​Smoking Prevention/lj [Legislation & Jurisprudence],​*Smoking Prevention/mt [Methods],​Tobacco Use Disorder/co [Complications],​Tobacco Use Disorder/et [Etiology],​*Tobacco Use Disorder/pc [Prevention & Control],​Vaping/ae [Adverse Effects],​Vaping/ec [Economics],​*Vaping/is [Instrumentation],​Vaping/lj [Legislation & Jurisprudence]  Abstract: CONTEXT: Rapid developments in e-cigarettes,​ or electronic nicotine delivery systems (ENDS),​ and the evolution of the overall tobacco product marketplace warrant frequent evaluation of the published literature. The purpose of this article is to report updated findings from a comprehensive review of the published scientific literature on ENDS.,​ EVIDENCE ACQUISITION: The authors conducted a systematic review of published empirical research literature on ENDS through May 31,​ 2016,​ using a detailed search strategy in the PubMed electronic database,​ expert review,​ and additional targeted searches. Included studies presented empirical findings and were coded to at least one of nine topics: (1) Product Features; (2) Health Effects; (3) Consumer Perceptions; (4) Patterns of Use; (5) Potential to Induce Dependence; (6) Smoking Cessation; (7) Marketing and Communication; (8) Sales; and (9) Policies; reviews and commentaries were excluded. Data from included studies were extracted by multiple coders (October 2015 to August 2016) into a standardized form and synthesized qualitatively by topic.,​ EVIDENCE SYNTHESIS: There were 687 articles included in this systematic review. The majority of studies assessed patterns of ENDS use and consumer perceptions of ENDS,​ followed by studies examining health effects of vaping and product features.,​ CONCLUSIONS: Studies indicate that ENDS are increasing in use,​ particularly among current smokers,​ pose substantially less harm to smokers than cigarettes,​ are being used to reduce/quit smoking,​ and are widely available. More longitudinal studies and controlled trials are needed to evaluate the impact of ENDS on population-level tobacco use and determine the health effects of longer-term vaping. Copyright © 2016 American Journal of Preventive Medicine. Published by Elsevier Inc. All rights reserved. |
| 180 | Riccardo Polosa,​ Pasquale Caponnetto (2017) E-cigarettes and smoking cessation: a critique of a New England Journal Medicine-commissioned case study.. Internal and emergency medicine; 12.  Keywords: Adult,​Electronic Nicotine Delivery Systems/mt [Methods],​*Electronic Nicotine Delivery Systems/sn [Statistics & Numerical Data],​Humans,​Male,​Meta-Analysis as Topic,​Risk Reduction Behavior,​*Smoking Cessation/mt [Methods],​Smoking Cessation/px [Psychology],​*Tobacco Use Cessation Devices/st [Standards]  Abstract: |
| 182 | Carlos Echevarria,​ Ian P Sinha (2017) Heterogeneity in the measurement and reporting of outcomes in studies of electronic cigarette use in adolescents: a systematic analysis of observational studies.. Tobacco control; 26.  Keywords: Adolescent,​Bias,​*Electronic Nicotine Delivery Systems/sn [Statistics & Numerical Data],​Humans,​Outcome Assessment (Health Care)/mt [Methods],​Prevalence,​Research Design,​*Smoking/ep [Epidemiology],​*Tobacco Products/sn [Statistics & Numerical Data]  Abstract: OBJECTIVE: To examine consistency between cross-sectional studies of conventional and electronic cigarette use among adolescents in terms of the measurement,​ analysis and reporting of parameters.,​ DESIGN: A systematic analysis of cross-sectional studies of conventional and electronic cigarette use in adolescents,​ to identify measured and reported parameters.,​ DATA SOURCES: Studies examining use of electronic and conventional cigarette use in adolescents were identified by searching the SCOPUS database in August 2015.,​ STUDY SELECTION: The selection criteria for studies were: cross-sectional studies,​ in English,​ on e-cigarette use in adolescents. Two reviewers independently selected relevant studies from the search. 60 abstracts were identified,​ from which 31 papers were eligible for review (23 unique studies).,​ DATA EXTRACTION: Measured and reported parameters were identified and tabulated. These included the prevalence of cigarette and/ or electronic cigarette use,​ and the definitions of terms. Data were extracted independently by two reviewers.,​ DATA SYNTHESIS: With regards basic parameters of 'ever' or 'current' use of electronic or conventional cigarettes,​ there were 31 unique measured parameters across 23 studies. Of 16/23 studies in which authors collected information on dual current use,​ prevalence was reported in 11/16,​ with six different definitions of 'dual use'.,​ CONCLUSIONS: There are substantial differences in measurement and reporting of parameters across observational studies of electronic and conventional cigarette use in adolescents. These studies are at risk of reporting bias,​ and results are difficult to interpret. A core outcome set that should be measured and reported in all observational studies is required,​ using structured consensus techniques. Copyright Published by the BMJ Publishing Group Limited. For permission to use (where not already granted under a licence) please go to http:www.bmj.com/company/products-services/rights-and-licensing/. |
| 186 | Gholamreza Heydari,​ Arezoo Ebn Ahmady,​ Fahimeh Chamyani,​ et al (2017) Electronic cigarette,​ effective or harmful for quitting smoking and respiratory health: A quantitative review papers.. Lung India : official organ of Indian Chest Society; 34.  Keywords:   Abstract: BACKGROUND: In recent years,​ electronic cigarettes (ECs) have been heavily advertised as an alternative smoking device as well as a possible cessation method. We aimed to review all published scientific literature pertaining to ECs and to present a simple conclusion about their effects for quitting smoking and respiratory health.,​ METHODS: This was a cross-sectional study with a search of PubMed,​ limited to English publications upto September 2014. The total number of papers which had ECs in its title and their conclusions positive or negative regarding ECs effects were computed. The number of negative papers was subtracted from the number of positive ones to make a score.,​ RESULTS: Of the 149 articles,​ 137 (91.9%) were accessible,​ of which 68 did not have inclusion criteria. In the 69 remaining articles,​ 24 studies supported ECs and 45 considered these to be harmful. Finally,​ based on this evidence,​ the score of ECs (computed result with positive minus negative) was -21.,​ CONCLUSION: Evidence to suggest that ECs may be effective and advisable for quitting smoking or a safe alternative for smoking is lacking and may instead harm the respiratory system. However,​ further studies are needed. |
| 189 | Konstantinos E Farsalinos,​ Riccardo Polosa (2014) Safety evaluation and risk assessment of electronic cigarettes as tobacco cigarette substitutes: a systematic review.. Therapeutic advances in drug safety; 5.  Keywords:   Abstract: Electronic cigarettes are a recent development in tobacco harm reduction. They are marketed as less harmful alternatives to smoking. Awareness and use of these devices has grown exponentially in recent years,​ with millions of people currently using them. This systematic review appraises existing laboratory and clinical research on the potential risks from electronic cigarette use,​ compared with the well-established devastating effects of smoking tobacco cigarettes. Currently available evidence indicates that electronic cigarettes are by far a less harmful alternative to smoking and significant health benefits are expected in smokers who switch from tobacco to electronic cigarettes. Research will help make electronic cigarettes more effective as smoking substitutes and will better define and further reduce residual risks from use to as low as possible,​ by establishing appropriate quality control and standards. |
| 191 | Brad Rodu (2011) The scientific foundation for tobacco harm reduction,​ 2006-2011.. Harm reduction journal; 8.  Keywords:   Abstract: Over the past five years there has been exponential expansion of interest in tobacco harm reduction (THR),​ with a concomitant increase in the number of published studies. The purpose of this manuscript is to review and analyze influential contributions to the scientific and medical literature relating to THR,​ and to discuss issues that continue to stimulate debate. Numerous epidemiologic studies and subsequent meta-analyses confirm that smokeless tobacco (ST) use is associated with minimal risks for cancer and for myocardial infarction; a small increased risk for stroke cannot be excluded. Studies from Sweden document that ST use is not associated with benign gastrointestinal disorders and chronic inflammatory diseases. Although any form of nicotine should be avoided during pregnancy,​ the highest risks for the developing baby are associated with smoking. It is documented that ST use has been a key factor in the declining rates of smoking and of smoking-related diseases in Sweden and Norway. For other countries,​ the potential population health benefits of ST are far greater than the potential risks. In follow-up studies,​ dual users of cigarettes and ST are less likely than exclusive smokers to achieve complete tobacco abstinence,​ but they are also less likely to be smoking. The health risks from dual use are probably lower than those from exclusive smoking. E-cigarette users are not exposed to the many toxicants,​ carcinogens and abundant free radicals formed when tobacco is burned. Although laboratory studies have detected trace concentrations of some contaminants,​ it is a small problem amenable to improvements in quality control and manufacturing that are likely with FDA regulation as tobacco products. There is limited evidence from clinical trials that e-cigarettes deliver only small doses of nicotine compared with conventional cigarettes. However,​ e-cigarette use emulates successfully the cigarette handling rituals and cues of cigarette smoking,​ which produces suppression of craving and withdrawal that is not entirely attributable to nicotine delivery. THR has been described as having "the potential to lead to one of the greatest public health breakthroughs in human history by fundamentally changing the forecast of a billion cigarette-caused deaths this century." |
| 195 | Jieming Zhong,​ Shuangshuang Cao,​ Weiwei Gong,​ et al (2016) Electronic Cigarettes Use and Intention to Cigarette Smoking among Never-Smoking Adolescents and Young Adults: A Meta-Analysis.. International journal of environmental research and public health; 13.  Keywords: Adolescent,​Adult,​Child,​Electronic Nicotine Delivery Systems/px [Psychology],​*Electronic Nicotine Delivery Systems/sn [Statistics & Numerical Data],​Humans,​*Intention,​Odds Ratio,​*Smoking/px [Psychology],​Young Adult  Abstract: Electronic cigarettes (e-cigarettes) use is becoming increasingly common,​ especially among adolescents and young adults,​ and there is little evidence on the impact of e-cigarettes use on never-smokers. With a meta-analysis method,​ we explore the association between e-cigarettes use and smoking intention that predicts future cigarette smoking. Studies were identified by searching three databases up to January 2016. The meta-analysis results were presented as pooled odds ratio (OR) with 95% confidence interval (CI) calculated by a fixed-effects model. A total of six studies (91,​051 participants,​ including 1452 with ever e-cigarettes use) were included in this meta-analysis study. We found that never-smoking adolescents and young adults who used e-cigarettes have more than 2 times increased odds of intention to cigarette smoking (OR = 2.21,​ 95% CI: 1.86-2.61) compared to those who never used,​ with low evidence of between-study heterogeneity (p = 0.28,​ I2 = 20.1%). Among never-smoking adolescents and young adults,​ e-cigarettes use was associated with increased smoking intention. |
| 196 | Muhannad Malas,​ Jan van der Tempel,​ Robert Schwartz,​ et al (2016) Electronic Cigarettes for Smoking Cessation: A Systematic Review.. Nicotine & tobacco research : official journal of the Society for Research on Nicotine and Tobacco; 18.  Keywords: Electronic Nicotine Delivery Systems/ae [Adverse Effects],​*Electronic Nicotine Delivery Systems/sn [Statistics & Numerical Data],​Humans,​Randomized Controlled Trials as Topic,​*Smoking Cessation/mt [Methods]  Abstract: BACKGROUND AND AIMS: Electronic cigarettes (e-cigarettes) have been steadily increasing in popularity among smokers,​ most of whom report using them to quit smoking. This study systematically reviews the current literature on the effectiveness of e-cigarettes as cessation aids.,​ METHODS: We searched PubMed,​ MEDLINE,​ PsycINFO,​ CINAHL,​ ERIC,​ ROVER,​ Scopus,​ ISI Web of Science,​ Cochrane Library,​ the Ontario Tobacco Research Unit (OTRU) library catalogue,​ and various gray literature sources. We included all English-language,​ empirical quantitative and qualitative papers that investigated primary cessation outcomes (smoking abstinence or reduction) or secondary outcomes (abstinence-related withdrawal symptoms and craving reductions) and were published on or before February 1,​ 2016.,​ RESULTS: Literature searches identified 2855 references. After removing duplicates and screening for eligibility,​ 62 relevant references were reviewed and appraised. In accordance with the GRADE system,​ the quality of the evidence in support of e-cigarettes' effectiveness in helping smokers quit was assessed as very low to low,​ and the evidence on smoking reduction was assessed as very low to moderate. The majority of included studies found that e-cigarettes,​ especially second-generation types,​ could alleviate smoking withdrawal symptoms and cravings in laboratory settings.,​ CONCLUSIONS: While the majority of studies demonstrate a positive relationship between e-cigarette use and smoking cessation,​ the evidence remains inconclusive due to the low quality of the research published to date. Well-designed randomized controlled trials and longitudinal,​ population studies are needed to further elucidate the role of e-cigarettes in smoking cessation.,​ IMPLICATIONS: This is the most comprehensive systematic evidence review to examine the relationship between e-cigarette use and smoking cessation among smokers. This review offers balanced and rigorous qualitative and quantitative analyses of published evidence on the effectiveness of e-cigarette use for smoking abstinence and reduction as well as important outcomes such as withdrawal symptoms and craving to smoke. While inconclusive due to low quality,​ overall the existing literature suggests e-cigarettes may be helpful for some smokers for quitting or reducing smoking. However,​ more carefully designed and scientifically sound studies are urgently needed to establish unequivocally the long-term cessation effects of e-cigarettes and to better understand of how and when e-cigarettes may be helpful. Copyright © The Author 2016. Published by Oxford University Press on behalf of the Society for Research on Nicotine and Tobacco. All rights reserved. For permissions,​ please e-mail: journals.permissions@oup.com. |
| 199 | S Khoudigian,​ T Devji,​ L Lytvyn,​ et al (2016) The efficacy and short-term effects of electronic cigarettes as a method for smoking cessation: a systematic review and a meta-analysis.. International journal of public health; 61.  Keywords: *Electronic Nicotine Delivery Systems,​Humans,​Nicotine/ad [Administration & Dosage],​Nicotine/ae [Adverse Effects],​*Nicotine,​Smoking/ae [Adverse Effects],​*Smoking Cessation/mt [Methods],​Substance Withdrawal Syndrome,​Tobacco Use Cessation Devices  Abstract: OBJECTIVES: E-cigarettes are increasingly popular as smoking cessation aids. This review assessed the efficacy of e-cigarettes for smoking cessation as well as desire to smoke,​ withdrawal symptoms,​ and adverse events in adult smokers.,​ METHODS: A systematic review was conducted. Studies comparing e-cigarettes to other nicotine replacement therapies or placebo were included. Data were pooled using meta-analysis.,​ RESULTS: Of 569 articles,​ 5 were eligible. Study participants were more likely to stop smoking when using nicotine e-cigarettes (43/489,​ 9 %) versus placebo e-cigarettes (8/173,​ 5 %); however,​ this difference was not statistically significant (RR 2.02; 95 % CI 0.97,​ 4.22). The pooled effect estimates for the desire to smoke (RR -0.22; 95 % CI -0.80,​ 0.36),​ irritability (RR -0.03; 95% CI -0.38,​ 0.31),​ restlessness (RR -0.03; 95 % CI -0.42,​ 0.35),​ poor concentration (RR -0.01; 95 % CI -0.35,​ 0.32),​ depression (RR -0.01; 95 % CI -0.22,​ 0.20),​ hunger (RR -0.01; 95 % CI -0.32,​ 0.30),​ and average number of non-serious adverse events (RR -0.09; 95 % CI -0.28,​ 0.46) were not statistically significantly different. Only one study reported serious adverse events with no apparent association with e-cigarette use.,​ CONCLUSIONS: Limited low-quality evidence of a non-statistically significant trend toward smoking cessation in adults using nicotine e-cigarettes exists compared with other therapies or placebo. Larger,​ high-quality studies are needed to inform policy decisions. |
| 200 | Sara Kalkhoran,​ Stanton A Glantz (2016) E-cigarettes and smoking cessation in real-world and clinical settings: a systematic review and meta-analysis.. The Lancet. Respiratory medicine; 4.  Keywords: *Electronic Nicotine Delivery Systems/sn [Statistics & Numerical Data],​Humans,​Motivation,​Odds Ratio,​Prevalence,​*Smoking/ep [Epidemiology],​Smoking/th [Therapy],​*Smoking Cessation/sn [Statistics & Numerical Data]  Abstract: BACKGROUND: Smokers increasingly use e-cigarettes for many reasons,​ including attempts to quit combustible cigarettes and to use nicotine where smoking is prohibited. We aimed to assess the association between e-cigarette use and cigarette smoking cessation among adult cigarette smokers,​ irrespective of their motivation for using e-cigarettes.,​ METHODS: PubMed and Web of Science were searched between April 27,​ 2015,​ and June 17,​ 2015. Data extracted included study location,​ design,​ population,​ definition and prevalence of e-cigarette use,​ comparison group (if applicable),​ cigarette consumption,​ level of nicotine dependence,​ other confounders,​ definition of quitting smoking,​ and odds of quitting smoking. The primary endpoint was cigarette smoking cessation. Odds of smoking cessation among smokers using e-cigarettes compared with smokers not using e-cigarettes were assessed using a random effects meta-analysis. A modification of the ACROBAT-NRSI tool and the Cochrane Risk of Bias Tool were used to assess bias. This meta-analysis is registered with PROSPERO (number CRD42015020382).,​ FINDINGS: 38 studies (of 577 studies identified) were included in the systematic review; all 20 studies with control groups (15 cohort studies,​ three cross-sectional studies,​ and two clinical trials) were included in random effects meta-analysis and sensitivity analyses. Odds of quitting cigarettes were 28% lower in those who used e-cigarettes compared with those who did not use e-cigarettes (odds ratio [OR] 0.72,​ 95% CI 0.57-0.91). Association of e-cigarette use with quitting did not significantly differ among studies of all smokers using e-cigarettes (irrespective of interest in quitting cigarettes) compared with studies of only smokers interested in cigarette cessation (OR 0.63,​ 95% CI 0.45-0.86 vs 0.86,​ 0.60-1.23; p=0.94). Other study characteristics (design,​ population,​ comparison group,​ control variables,​ time of exposure assessment,​ biochemical verification of abstinence,​ and definition of e-cigarette use) were also not associated with the overall effect size (p>=0.77 in all cases).,​ INTERPRETATION: As currently being used,​ e-cigarettes are associated with significantly less quitting among smokers.,​ FUNDING: National Institutes of Health,​ National Cancer Institute,​ FDA Center for Tobacco Products. Copyright © 2016 Elsevier Ltd. All rights reserved. |
| 201 | Meng Wang,​ Jian-Wei Wang,​ Shuang-Shuang Cao,​ et al (2016) Cigarette Smoking and Electronic Cigarettes Use: A Meta-Analysis.. International journal of environmental research and public health; 13.  Keywords: Adolescent,​Adult,​*Electronic Nicotine Delivery Systems/sn [Statistics & Numerical Data],​Europe/ep [Epidemiology],​Humans,​Models,​ Statistical,​Odds Ratio,​*Smoking/ep [Epidemiology],​United States/ep [Epidemiology]  Abstract: Increasing evidence indicates that cigarette smoking is a strong predictor of electronic cigarettes (e-cigarettes) use,​ particularly in adolescents,​ yet the effects has not be systematically reviewed and quantified. Relevant studies were retrieved by searching three databases up to June 2015. The meta-analysis results were presented as pooled odds ratios (ORs) with 95% confidence intervals (CIs) calculated by a random-effects model. Current smokers were more likely to use e-cigarette currently (OR: 14.89,​ 95% CI: 7.70-28.78) and the probability was greater in adolescents than in adults (39.13 vs. 7.51). The probability of ever e-cigarettes use was significantly increased in smokers (OR: 14.67,​ 95% CI: 11.04-19.49). Compared with ever smokers and adults,​ the probabilities were much greater in current smokers (16.10 vs. 9.47) and adolescents (15.19 vs. 14.30),​ respectively. Cigarette smoking increases the probability of e-cigarettes use,​ especially in current smokers and adolescents. |
| 203 | Charlotte Leduc,​ Elisabeth Quoix (2016) Is there a role for e-cigarettes in smoking cessation?.. Therapeutic advances in respiratory disease; 10.  Keywords: Administration,​ Inhalation,​*Electronic Nicotine Delivery Systems,​Humans,​*Nicotine/ad [Administration & Dosage],​Nicotine/ae [Adverse Effects],​*Nicotinic Agonists/ad [Administration & Dosage],​Nicotinic Agonists/ae [Adverse Effects],​Randomized Controlled Trials as Topic,​Risk Factors,​Smoking/ae [Adverse Effects],​*Smoking Cessation/mt [Methods],​*Smoking Prevention,​Tobacco Use Disorder/di [Diagnosis],​*Tobacco Use Disorder/dt [Drug Therapy],​Treatment Outcome  Abstract: The use of e-cigarettes has dramatically increased over the past few years and their role in smoking cessation remains controversial. Several clinical studies have evaluated their efficacy in smoking cessation but most of them are prospective cohort studies. Only two randomized,​ controlled trials have compared e-cigarettes versus placebo or patches. A meta-analysis of these two randomized,​ controlled trials has been performed. Nicotine-containing e-cigarettes appear to help smokers unable to stop smoking altogether to reduce their cigarette consumption when compared with placebo. However,​ these results are rated 'low' by GRADE standards. Many cohort studies have been conducted,​ with contradictory results. For some,​ e-cigarettes could increase the risk of nonsmokers developing nicotine dependence and of current smokers maintaining their dependence. The debate remains open and more randomized trials are needed with long-term data about the efficacy and safety of e-cigarettes. Copyright © The Author(s),​ 2015. |
| 207 | Carrie D Patnode,​ Jillian T Henderson,​ Jamie H Thompson,​ et al (2015) Behavioral Counseling and Pharmacotherapy Interventions for Tobacco Cessation in Adults,​ Including Pregnant Women: A Review of Reviews for the U.S. Preventive Services Task Force.. Annals of internal medicine; 163.  Keywords: Adult,​*Behavior Therapy,​Bupropion/ae [Adverse Effects],​Bupropion/tu [Therapeutic Use],​*Counseling,​Electronic Nicotine Delivery Systems/ae [Adverse Effects],​Female,​Humans,​Nicotinic Agonists/ae [Adverse Effects],​Nicotinic Agonists/tu [Therapeutic Use],​Pregnancy,​*Smoking Cessation/mt [Methods],​*Smoking Prevention,​Tobacco Use Cessation Devices/ae [Adverse Effects],​*Tobacco Use Cessation Devices,​United States,​Varenicline/ae [Adverse Effects],​Varenicline/tu [Therapeutic Use]  Abstract: BACKGROUND: Tobacco use is the leading cause of preventable death in the United States.,​ PURPOSE: To review the effectiveness and safety of pharmacotherapy and behavioral interventions for tobacco cessation.,​ DATA SOURCES: 5 databases and 8 organizational Web sites were searched through 1 August 2014 for systematic reviews,​ and PubMed was searched through 1 March 2015 for trials on electronic nicotine delivery systems.,​ STUDY SELECTION: Two reviewers examined 114 articles to identify English-language reviews that reported health,​ cessation,​ or adverse outcomes.,​ DATA EXTRACTION: One reviewer abstracted data from good- and fair-quality reviews,​ and a second checked for accuracy.,​ DATA SYNTHESIS: 54 reviews were included. Behavioral interventions increased smoking cessation at 6 months or more (physician advice had a pooled risk ratio [RR] of 1.76 [95% CI,​ 1.58 to 1.96]). Nicotine replacement therapy (RR,​ 1.60 [CI,​ 1.53 to 1.68]),​ bupropion (RR,​ 1.62 [CI,​ 1.49 to 1.76]),​ and varenicline (RR,​ 2.27 [CI,​ 2.02 to 2.55]) were also effective for smoking cessation. Combined behavioral and pharmacotherapy interventions increased cessation by 82% compared with minimal intervention or usual care (RR,​ 1.82 [CI,​ 1.66 to 2.00]). None of the drugs were associated with major cardiovascular adverse events. Only 2 trials addressed efficacy of electronic cigarettes for smoking cessation and found no benefit. Among pregnant women,​ behavioral interventions benefited cessation and perinatal health; effects of nicotine replacement therapy were not significant.,​ LIMITATION: Evidence published after each review's last search date was not included.,​ CONCLUSION: Behavioral and pharmacotherapy interventions improve rates of smoking cessation among the general adult population,​ alone or in combination. Data on the effectiveness and safety of electronic nicotine delivery systems are limited.,​ PRIMARY FUNDING SOURCE: Agency for Healthcare Research and Quality. |
| 210 | Muhammad Aziz Rahman,​ Nicholas Hann,​ Andrew Wilson,​ et al (2015) E-cigarettes and smoking cessation: evidence from a systematic review and meta-analysis.. PloS one; 10.  Keywords: *Electronic Nicotine Delivery Systems,​Humans,​Nicotine/an [Analysis],​Outcome Assessment (Health Care),​*Smoking Cessation/mt [Methods]  Abstract: BACKGROUND: E-cigarettes are currently being debated regarding their possible role in smoking cessation and as they are becoming increasingly popular,​ the research to date requires investigation.,​ OBJECTIVES: To investigate whether the use of e-cigarettes is associated with smoking cessation or reduction,​ and whether there is any difference in efficacy of e-cigarettes with and without nicotine on smoking cessation.,​ DATA SOURCES: A systematic review of articles with no limit on publication date was conducted by searching PubMed,​ Web of Knowledge and Scopus databases.,​ METHODS: Published studies,​ those reported smoking abstinence or reduction in cigarette consumption after the use of e-cigarettes,​ were included. Studies were systematically reviewed,​ and meta-analyses were conducted using Mantel-Haenszel fixed-effect and random-effects models. Degree of heterogeneity among studies and quality of the selected studies were evaluated.,​ RESULTS: Six studies were included involving 7,​551 participants. Meta-analyses included 1,​242 participants who had complete data on smoking cessation. Nicotine filled e-cigarettes were more effective for cessation than those without nicotine (pooled Risk Ratio 2.29,​ 95%CI 1.05-4.97). Amongst 1,​242 smokers,​ 224 (18%) reported smoking cessation after using nicotine-enriched e-cigarettes for a minimum period of six months. Use of such e-cigarettes was positively associated with smoking cessation with a pooled Effect Size of 0.20 (95%CI 0.11-0.28). Use of e-cigarettes was also associated with a reduction in the number of cigarettes used.,​ LIMITATIONS: Included studies were heterogeneous,​ due to different study designs and gender variation. Whilst we were able to comment on the efficacy of nicotine vs. non-nicotine e-cigarettes for smoking cessation,​ we were unable to comment on the efficacy of e-cigarettes vs. other interventions for cessation,​ given the lack of comparator groups in the studies included in this meta-analysis.,​ CONCLUSIONS: Use of e-cigarettes is associated with smoking cessation and reduction. More randomised controlled trials are needed to assess effectiveness against other cessation methods. |
| 215 | Maria Rosaria Gualano,​ Stefano Passi,​ Fabrizio Bert,​ et al (2015) Electronic cigarettes: assessing the efficacy and the adverse effects through a systematic review of published studies.. Journal of public health (Oxford,​ England); 37.  Keywords: *Electronic Nicotine Delivery Systems/ae [Adverse Effects],​Electronic Nicotine Delivery Systems/px [Psychology],​Humans,​Smoking/ep [Epidemiology],​Smoking/px [Psychology]  Abstract: BACKGROUND: To investigate the efficacy and the adverse effects (AEs) of the electronic cigarette,​ we performed a systematic review of published studies.,​ METHODS: We selected experimental and observational studies examining the efficacy (as reduction of desire to smoke and/or number of cigarettes smoked and/or quitting or as reduction of nicotine withdrawal symptoms) and the safety of EC (AEs self-reported or clinical/laboratory). The following search engines were used: PubMed,​ ISI Web of Knowledge and Cochrane Controlled Trials Register.,​ RESULTS: Finally,​ six experimental studies and six cohort studies were included. In the prospective 12-month,​ randomized controlled trial,​ smoking reduction was documented in 22.3 and 10.3% at Weeks 12 and 52,​ respectively (P < 0.001 versus baseline). Moreover,​ two cohort studies reported a reduction in the number of cigarette/day (from 50 to 80%) after the introduction of the EC. 'Mouth and throat irritation',​ 'nausea',​ 'headache' and 'dry cough' were the most frequently AEs reported.,​ CONCLUSIONS: The use of the EC can reduce the number of cigarettes smoked and withdrawal symptoms,​ but the AEs reported are mainly related to a short period of use. Long-term studies are needed to evaluate the effects of the EC usage after a chronic exposure. Copyright © The Author 2014. Published by Oxford University Press on behalf of Faculty of Public Health. All rights reserved. For permissions,​ please e-mail: journals.permissions@oup.com. |
| 217 | Katherine Kelly Orr,​ Nicole J Asal (2014) Efficacy of electronic cigarettes for smoking cessation.. The Annals of pharmacotherapy; 48.  Keywords: *Electronic Nicotine Delivery Systems,​Humans,​Nicotine/ae [Adverse Effects],​*Smoking Cessation/mt [Methods],​Substance Withdrawal Syndrome/pc [Prevention & Control],​Tobacco Use Cessation Devices  Abstract: OBJECTIVE: To review data demonstrating effective smoking cessation with electronic cigarettes (e-cigarettes).,​ DATA SOURCES: A literature search of MEDLINE/PubMed (1946-March 2014) was performed using the search terms e-cigarettes,​ electronic cigarettes,​ and smoking cessation. Additional references were identified from a review of literature citations.,​ STUDY SELECTION AND DATA EXTRACTION: All English-language clinical studies assessing efficacy of e-cigarettes compared with baseline,​ placebo,​ or other pharmacological methods to aid in withdrawal symptoms,​ smoking reduction,​ or cessation were evaluated.,​ DATA SYNTHESIS: A total of 6 clinical studies were included in the review. In small studies,​ e-cigarettes significantly decreased desire to smoke,​ number of cigarettes smoked per day,​ and exhaled carbon monoxide levels. Symptoms of nicotine withdrawal and adverse effects were variable. The most common adverse effects were nausea,​ headache,​ cough,​ and mouth/throat irritation. Compared with nicotine patches,​ e-cigarettes were associated with fewer adverse effects and higher adherence. Most studies showed a significant decrease in cigarette use acutely; however,​ long-term cessation was not sustained at 6 months.,​ CONCLUSIONS: There is limited evidence for the effectiveness of e-cigarettes in smoking cessation; however,​ there may be a place in therapy to help modify smoking habits or reduce the number of cigarettes smoked. Studies available provided different administration patterns such as use while smoking,​ instead of smoking,​ or as needed. Short-term studies reviewed were small and did not necessarily evaluate cessation with a focus on parameters associated with cessation withdrawal symptoms. Though long-term safety is unknown,​ concerns regarding increased poisoning exposures among adults in comparison with cigarettes are alarming. Copyright © The Author(s) 2014. |
| 218 | Dennis Nowak,​ Rudolf A Jorres,​ Tobias Ruther (2014) E-cigarettes--prevention,​ pulmonary health,​ and addiction.. Deutsches Arzteblatt international; 111.  Keywords: Causality,​Comorbidity,​*Electronic Nicotine Delivery Systems/mo [Mortality],​*Electronic Nicotine Delivery Systems/sn [Statistics & Numerical Data],​Evidence-Based Medicine,​Humans,​Incidence,​*Lung Diseases/mo [Mortality],​Risk Assessment,​*Smoking/mo [Mortality],​Smoking Cessation/mt [Methods],​*Smoking Cessation/sn [Statistics & Numerical Data],​*Smoking Prevention,​Survival Rate,​*Tobacco Use Disorder/mo [Mortality],​Treatment Outcome  Abstract: BACKGROUND: E-cigarettes are coming into wider use. They are advertised as an aid to smoking cessation,​ but there is concern that they may also serve as a gateway drug for cigarette smoking.,​ METHOD: The authors systematically searched the PubMed database for relevant publications on the mechanism of action of e-cigarettes,​ the nature of their emissions,​ their assessment by potential users,​ their efficacy in smoking cessation,​ and their potential for addiction.,​ RESULTS: There have been many reports of epidemiologically uninformative case series in which smokers were helped to stop smoking by the use of e- cigarettes. Only two controlled trials have shown that e-cigarettes have approximately the same effect as nicotine substitution therapy when used as an aid to smoking cessation. The effect is nearly independent of nicotine content. E-cigarettes are also consumed,​ to a small extent,​ by nonsmokers. As far as can be estimated toxicologically at present,​ the danger to active and passive smokers of e-cigarettes is presumably orders of magnitude less than that of tobacco smokers,​ although the variable composition of the fluids used in e-cigarettes introduces a degree of uncertainty.,​ CONCLUSION: Preclinical and initial clinical data,​ including some data from randomized controlled trials,​ indicate that e-cigarettes may be useful as an aid to smoking cessation or as a means of lowering risk in high-risk groups. In contrast to the demonstrated efficacy of multimodal smoking-cessation programs with pharmacological and psychotherapeutic support,​ the efficacy of e-cigarettes in smoking cessation has not yet been satisfactorily shown. Valid and informative clinical trials are urgently needed. These should also be designed to determine what predisposition(s),​ if any,​ might make the use of e-cigarettes more or less successful than that of other aids to smoking cessation. Moreover,​ e-cigarettes might be a gateway drug for cigarette smoking; thus,​ no clear recommendation about their use can be made at present. |
| 222 | Jessica K Pepper,​ Noel T Brewer (2014) Electronic nicotine delivery system (electronic cigarette) awareness,​ use,​ reactions and beliefs: a systematic review.. Tobacco control; 23.  Keywords: *Attitude,​Electronic Nicotine Delivery Systems/sn [Statistics & Numerical Data],​*Electronic Nicotine Delivery Systems,​Electronics,​Humans,​*Motivation,​*Nicotine/ad [Administration & Dosage],​*Smoking Cessation,​*Smoking Prevention,​Tobacco Products  Abstract: OBJECTIVE: We sought to systematically review the literature on electronic nicotine delivery systems (ENDS,​ also called electronic cigarettes) awareness,​ use,​ reactions and beliefs.,​ DATA SOURCES: We searched five databases for articles published between 2006 and 1 July 2013 that contained variations of the phrases 'electronic cigarette',​ 'e-cigarette' and 'electronic nicotine delivery'.,​ STUDY SELECTION: Of the 244 abstracts identified,​ we excluded articles not published in English,​ articles unrelated to ENDS,​ dissertation abstracts and articles without original data on prespecified outcomes.,​ DATA EXTRACTION: Two reviewers coded each article for ENDS awareness,​ use,​ reactions and beliefs.,​ DATA SYNTHESIS: 49 studies met inclusion criteria. ENDS awareness increased from 16% to 58% from 2009 to 2011,​ and use increased from 1% to 6%. The majority of users were current or former smokers. Many users found ENDS satisfying,​ and some engaged in dual use of ENDS and other tobacco. No longitudinal studies examined whether ENDS serve as 'gateways' to future tobacco use. Common reasons for using ENDS were quitting smoking and using a product that is healthier than cigarettes. Self-reported survey data and prospective trials suggest that ENDS might help cigarette smokers quit,​ but no randomised controlled trials with probability samples compared ENDS with other cessation tools. Some individuals used ENDS to avoid smoking restrictions.,​ CONCLUSIONS: ENDS use is expanding rapidly despite experts' concerns about safety,​ dual use and possible 'gateway' effects. More research is needed on effective public health messages,​ perceived health risks,​ validity of self-reports of smoking cessation and the use of different kinds of ENDS. Copyright Published by the BMJ Publishing Group Limited. For permission to use (where not already granted under a licence) please go to http:group.bmj.com/group/rights-licensing/permissions. |
| 226 | Brent Caldwell,​ Walt Sumner,​ Julian Crane (2012) A systematic review of nicotine by inhalation: is there a role for the inhaled route?.. Nicotine & tobacco research : official journal of the Society for Research on Nicotine and Tobacco; 14.  Keywords: Administration,​ Inhalation,​Adult,​Female,​Humans,​Lung,​Male,​Metered Dose Inhalers,​*Nicotine/ad [Administration & Dosage],​*Smoking/th [Therapy],​*Smoking Cessation/mt [Methods],​Tobacco Products  Abstract: INTRODUCTION: A considerable minority of adults remain addicted to smoking cigarettes despite substantial education and public health efforts. Nicotine replacement therapies have only modest long-term quit rates. The pulmonary route of nicotine delivery has advantages over other routes. However,​ there are regulatory and technical barriers to the development of pulmonary nicotine delivery devices,​ and hence,​ none are commercially available. Current knowledge about pulmonary nicotine delivery is scattered throughout the literature and other sources such as patent applications. This review draws together what is currently known about pulmonary nicotine delivery and identifies potential ways that deep lung delivery can be achieved with a simple portable device.,​ AIMS: To systematically review clinical trials of nicotine inhalers,​ determine whether they delivered nicotine via the lung,​ and identify ways that pulmonary delivery of medicinal nicotine might be achieved and the technical issues involved.,​ METHODS: Systematic search of Medline and Embase.,​ RESULTS: Thirty-eight trials met the inclusion criteria. Cough,​ reflex interruption of smooth inspiration,​ and throat scratch limited the usefulness of nicotine inhalers. The pharmacokinetic profiles of portable nicotine inhalers were inferior to smoking,​ but among commercially available products,​ electronic cigarettes are currently the most promising.,​ CONCLUSIONS: Pulmonary nicotine delivery might be maximized by use of nicotine salts,​ which have a more physiological pH than pure nicotine,​ by ensuring the mass of the particles is optimal for alveolar absorption,​ and by adding flavoring agents. Metered-dose inhalers potentially can deliver nicotine more efficiently than other nicotine products,​ facilitating smoking cessation and improving smokers' lives. |
| 241 | Theodore Lloyd (2015) Stubbing out smoking in schizophrenia.. The Psychologist; 28.  Keywords: *Schizophrenia,​*Tobacco Smoking,​Health,​Risk Factors,​Smoking Cessation,​Treatment Guidelines  Abstract: Discusses smoking in schizophrenia. A worldwide meta-analysis has estimated that 62 per cent of those with a diagnosis of schizophrenia are smokers,​ nearly three times more than the general population. No one theory definitively explains the relationship,​ although many exist. Broadly speaking,​ these fall into one of three categories: (a) some aspect of schizophrenia may lead more people with the illness to smoke; (b) tobacco smoking is itself a risk factor for schizophrenia; or (c) genetic and/or environmental factors might lead to an addiction to nicotine and also to schizophrenia. Within this,​ hypotheses range from psychological social and biological in nature,​ with some evidence to support them all as plausible explanations. Environmental and social factors certainly play some role in the excessively high smoking rates. Unemployment,​ poverty,​ limited education,​ peer influence and even the mental health treatment system all increase the risk of smoking for people with schizophrenia. Suggestions are made for mental health professionals,​ with the recommendation that all should be offered support to help stop smoking. However,​ guidelines are noticeably silent on the subject of e-cigarettes. While not proposing e-cigarettes as a solution,​ the author suggests that they might offer a partial solution. (PsycINFO Database Record (c) 2017 APA,​ all rights reserved) |
| 245 | A Glasser,​ H Abudayyeh,​ J Cantrell,​ et al (2019) Patterns of E-Cigarette Use Among Youth and Young Adults: Review of the Impact of E-Cigarettes on Cigarette Smoking.. Nicotine & tobacco research : official journal of the Society for Research on Nicotine and Tobacco; 21.  Keywords:   Abstract: There is concern that e-cigarette use among youth and young adults (YAs) may lead to future cigarette or other combustible tobacco product use. A synthesis of the literature on this topic is needed because existing longitudinal studies are limited in number and not consistent in their conclusions. We conducted a search in PubMed through December 31,​ 2017 for peer-reviewed studies related to e-cigarette patterns of use. Of 588 relevant studies,​ 26 had a youth or YA sample,​ were longitudinal in design,​ and assessed e-cigarette use at baseline and cigarette smoking at follow-up. Most studies followed a sample over time and compared cigarette smoking at follow-up between baseline e-cigarette users and nonusers. Other studies examined the difference at follow-up in cigarette smoking status among smokers according to e-cigarette use at baseline. Results suggest that,​ among never smokers,​ e-cigarette use is associated with the future (6 months to 2.5 years) cigarette trial; however,​ firm conclusions cannot be drawn because of limitations including small sample size,​ measurement of experimental use (ie,​ ever use,​ past 30-day use) rather than established use,​ and inadequate controls for potentially confounding variables. Conclusions also cannot be drawn from studies examining the impact of e-cigarette use among smokers due to the limited number of studies and additional limitations. A comprehensive understanding of this literature is needed to inform policy makers and consumers for evidence-based decision-making and to guide future research on e-cigarette use among youth and young adults. IMPLICATIONS: The present article provides a review of the impact of e-cigarette use on subsequent cigarette smoking among youth and YAs. Studies presented here suggest that e-cigarette use among nonsmokers is associated with subsequent cigarette smoking,​ but study designs are subject to numerous limitations. Future research should focus on addressing the characteristics that put youth and YAs at the risk of using either product and how appeal and accessibility of these products are related to product use in order to inform future policy-making. |
| 246 | PT Harrell,​ VN Simmons,​ JB Correa,​ et al (2014) Electronic nicotine delivery systems ("e-cigarettes"): review of safety and smoking cessation efficacy.. Otolaryngology--head and neck surgery : official journal of American Academy of Otolaryngology-Head and Neck Surgery; 151.  Keywords:   Abstract: BACKGROUND AND OBJECTIVES: Cigarette smoking is common among cancer patients and is associated with negative outcomes. Electronic nicotine delivery systems ("e-cigarettes") are rapidly growing in popularity and use,​ but there is limited information on their safety or effectiveness in helping individuals quit smoking. DATA SOURCES: The authors searched PubMed,​ Web of Science,​ and additional sources for published empirical data on safety and use of electronic cigarettes as an aid to quit smoking. REVIEW METHODS: We conducted a structured search of the current literature up to and including November 2013. RESULTS: E-cigarettes currently vary widely in their contents and are sometimes inconsistent with labeling. Compared to tobacco cigarettes,​ available evidence suggests that e-cigarettes are often substantially lower in toxic content,​ cytotoxicity,​ associated adverse effects,​ and secondhand toxicity exposure. Data on the use of e-cigarettes for quitting smoking are suggestive but ultimately inconclusive. CONCLUSIONS: Clinicians are advised to be aware that the use of e-cigarettes,​ especially among cigarette smokers,​ is growing rapidly. These devices are unregulated,​ of unknown safety,​ and of uncertain benefit in quitting smoking. IMPLICATIONS FOR PRACTICE: In the absence of further data or regulation,​ oncologists are advised to discuss the known and unknown safety and efficacy information on e-cigarettes with interested patients and to encourage patients to first try FDA-approved pharmacotherapies for smoking cessation. |
| 247 | Selph,​ Shelley,​ Patnode,​ et al (2020) . #journal#; .  Keywords:   Abstract: BACKGROUND: Interventions to discourage use of tobacco products among children and adolescents may help decrease tobacco-related illness. Tobacco products for this review include electronic nicotine delivery systems,​ often referred to as e-cigarettes.,​ PURPOSE: To systematically update the 2013 U.S. Preventive Services Task Force (USPSTF) review on primary care relevant interventions for tobacco use prevention and cessation in children and adolescents.,​ DATA SOURCES: We searched the Cochrane Central Register of Controlled Trials and Cochrane Database of Systematic Reviews,​ MEDLINE,​ PsycINFO,​ and EMBASE (September 1,​ 2012 to June 25,​ 2019) with surveillance through February 7,​ 2020.,​ STUDY SELECTION: We selected primary care relevant studies based on inclusion and exclusion criteria developed for each key question. We included randomized and nonrandomized controlled trials of children and adolescents up to 18 years of age for cessation and 25 years of age for prevention. Trials that compared behavioral or pharmacological interventions with a no or minimal smoking intervention control group (e.g.,​ usual care,​ attention control,​ wait list) were included.,​ DATA EXTRACTION: One investigator abstracted data and a second investigator checked data abstraction for accuracy. Two investigators independently assessed study quality using methods developed by the USPSTF.,​ DATA SYNTHESIS (RESULTS): Twenty-six trials met inclusion criteria. Behavioral interventions were associated with decreased likelihood of smoking initiation compared with control interventions (k=13,​ n=21,​700; 7.4% vs. 9.2%; relative risk [RR] 0.82,​ 95% confidence interval [CI] 0.73 to 0.92). In trials restricted to smokers,​ behavioral interventions had no effect on smoking prevalence (k=9,​ n=2,​516,​ 80.7% vs. 84.1% continued smoking,​ RR 0.97,​ 95% CI,​ 0.93 to 1.01). Behavioral interventions were more effective than control interventions at decreasing smoking prevalence in trials of smokers and nonsmokers (k=7,​ n=10,​533; 16.8% vs. 20.1%; RR 0.91,​ 95% CI,​ 0.83 to 0.995). However,​ these results were sensitive to inclusion of two trials of very intensive interventions. Two trials of bupropion and one trial of nicotine replacement therapy found no significant benefits of medication on likelihood of smoking cessation. One trial each found no evidence for a beneficial intervention effect on health outcomes or on adult smoking.,​ LIMITATIONS: Few trials addressed the prevention or cessation of tobacco products other than cigarettes; no trials evaluated effects of interventions on e-cigarette use. Trials of pharmacotherapy were few and had small sample sizes.,​ CONCLUSIONS: Behavioral interventions can reduce the likelihood of smoking initiation in nonsmoking youth and young adults. Research is needed to identify effective behavioral interventions for youth who smoke or who use other tobacco products and to understand the effectiveness of pharmacotherapy on cessation. Due to the rapid escalation of e-cigarette use among youth,​ both prevention and cessation trials that target and/or include e-cigarettes are imminently needed. |
| 251 | Arakeri,​ G,​ Patil,​ et al (2020) Are electronic nicotine delivery systems (ENDs) helping cigarette smokers quit?-Current evidence. Journal of Oral Pathology and Medicine; 49.  Keywords: *cigarette smoking,​comparative study,​human,​Newcastle-Ottawa scale,​priority journal,​review,​risk assessment,​smokeless tobacco,​smoking,​*smoking cessation,​systematic review,​tobacco,​tobacco use,​nicotine,​nicotine gum,​nicotine patch,​*electronic cigarette  Abstract: Background: Electronic cigarettes (e-cigarettes) and other electronic vaping devices are commonly used as a method to help tobacco smoking cessation. However,​ the data on their safety and efficacy are currently scarce. The aim of this review was to explore the role of e-cigarettes in tobacco cessation among tobacco users. Types of Studies Reviewed: PubMed,​ EMBASE,​ Scopus,​ Web of Science,​ and grey literature from January 1990 were searched up to and including September 2018. Two independent reviewers performed the study selection according to eligibility criteria. Result(s): A total of 13 studies that met the eligibility criteria were included. The majority of studies were done in the USA,​ and all studies were longitudinal cohort studies. The odds of increased smoking cessation in association with e-cigarette use ranged from onefold to sixfolds. No significant increase in smoking cessation was found among e-cigarette users compared with non-e-cigarette users. Pattern of e-cigarette had positive influence on smoking cessation among users. Conclusions and Practical Implications: E-cigarette use was not significantly associated with increased smoking cessation among cigarette smokers. We also found that pattern of e-cigarette may have significant effect on smoking cessation capability of e-cigarettes. Well-designed randomized controlled clinical trials are needed to assess the clinical efficacy of e-cigarettes in comparison with approved smoking cessation therapies.Copyright © 2019 John Wiley & Sons A/S. Published by John Wiley & Sons Ltd |
| 264 | Asher,​ Tim,​ Belden,​ et al (2019) Does using e-cigarettes increase cigarette smoking in adolescents?. The Journal of family practice; 68.  Keywords: Adolescent,​*Adolescent Behavior/px [Psychology],​*Cigarette Smoking/px [Psychology],​Cohort Studies,​Electronic Nicotine Delivery Systems,​Female,​Humans,​Male,​Prospective Studies,​Risk Factors,​*Vaping/ae [Adverse Effects],​*Vaping/px [Psychology]  Abstract: Probably. Electronic cigarette (e-cigarette) use by adolescents is associated with a 2- to 4-fold increase in cigarette smoking over the next year (strength of recommendation: A,​ meta-analysis and subsequent prospective cohort studies). |
| 271 | Villalobos,​ RE,​ Ambrocio,​ et al (2019) Electronic cigarettes for smoking cessation: An individual patient meta-analysis of randomized controlled trials. European Respiratory Journal; 54.  Keywords: adult,​conference abstract,​controlled study,​*electronic cigarette,​female,​human,​incidence,​male,​meta analysis,​patient coding,​randomized controlled trial (topic),​*smoking cessation,​systematic review,​placebo  Abstract: Background: Electronic cigarettes have been gaining popularity as smoking cessation tools worldwide but its safety and effectiveness compared to placebo and nicotine replacement therapy (NRT) is still undefined. Randomized controlled trials are severely lacking and conflicting in results. Method(s): We conducted a meta-analysis of individual patient data involved in randomized controlled trials examining the effectiveness of e-cigarettes vs. NRT or placebo primarily in terms of sustained abstinence rates at six months. Safety was assessed via the incidence of any serious adverse events. Result(s): Six randomized controlled trials were included in the analysis. The most recent study was published in 2019. Six-month sustained abstinence rate was higher in the e-cigarette versus NRT group,​ 1.69 (1.30,​ 2.21). Consistently,​ six-month abstinence rate in e-cigarette group was superior to placebo,​ 2.37 (1.19,​ 4.74). There was a trend toward increased total serious adverse in the e-cigarette group vs. NRT,​ although this is not significant,​ RR= 1.42 (0.94,​ 2.12). Conclusion(s): In our meta-analysis,​ we illustrated that e-cigarettes were superior to both NRT and placebo in inducing sustained smoking abstinence. However,​ the safety of e-cigarettes is not clearly assessed and more randomized controlled trials are definitely needed. (Figure Presented) . |
| 281 | Campbell,​ Katarzyna,​ Coleman-Haynes,​ et al (2020) Factors influencing the uptake and use of nicotine replacement therapy and e-cigarettes in pregnant women who smoke: a qualitative evidence synthesis. The Cochrane database of systematic reviews; 5.  Keywords:   Abstract: BACKGROUND: Nicotine replacement therapy (NRT) delivers nicotine without the toxic chemicals present in tobacco smoke. It is an effective smoking cessation aid in non-pregnant smokers,​ but there is less evidence of effectiveness in pregnancy. Systematic review evidence suggests that pregnant women do not adhere to NRT as prescribed,​ which might undermine effectiveness. Electronic cigarettes (e-cigarettes) have grown in popularity,​ but effectiveness and safety in pregnancy are not yet established. The determinants of uptake and use of NRT and e-cigarettes in pregnancy are unknown.,​ OBJECTIVES: To explore factors affecting uptake and use of NRT and e-cigarettes in pregnancy.,​ SEARCH METHODS: We searched MEDLINE(R),​ CINAHL and PsycINFO on 1 February 2019. We manually searched OpenGrey database and screened references of included studies and relevant reviews. We also conducted forward citation searches of included studies.,​ SELECTION CRITERIA: We selected studies that used qualitative methods of data collection and analysis,​ included women who had smoked in pregnancy,​ and elicited participants' views about using NRT/e-cigarettes for smoking cessation or harm reduction (i.e. to smoke fewer cigarettes) during pregnancy.,​ DATA COLLECTION AND ANALYSIS: We identified determinants of uptake and use of NRT/e-cigarettes in pregnancy using a thematic synthesis approach. Two review authors assessed the quality of included studies with the Wallace tool. Two review authors used the CERQual approach to assess confidence in review findings. The contexts of studies from this review and the relevant Cochrane effectiveness review were not similar enough to fully integrate findings; however,​ we created a matrix to juxtapose findings from this review with the descriptions of behavioural support from trials in the effectiveness review.,​ MAIN RESULTS: We included 21 studies: 15 focused on NRT,​ 3 on e-cigarettes,​ and 3 on both. Studies took place in five high-income countries. Most studies contributed few relevant data; substantially fewer data were available on determinants of e-cigarettes. Many studies focused predominantly on issues relating to smoking cessation,​ and determinants of NRT/e-cigarette use was often presented as one of the themes. We identified six descriptive themes and 18 findings within those themes; from these we developed three overarching analytical themes representing key determinants of uptake and adherence to NRT and/or e-cigarettes in pregnancy. The analytical themes show that women's desire to protect their unborn babies from harm is one of the main reasons they use these products. Furthermore,​ women consider advice from health professionals when deciding whether to use NRT or e-cigarettes; when health professionals tell women that NRT or e-cigarettes are safer than smoking and that it is okay for them to use these in pregnancy,​ women report feeling more confident about using them. Conversely,​ women who are told that NRT or e-cigarettes are as dangerous or more dangerous than smoking and that they should not use them during pregnancy feel less confident about using them. Women's past experiences with NRT can also affect their willingness to use NRT in pregnancy; women who feel that NRT had worked for them (or someone they know) in the past were more confident about using it again. However,​ women who had negative experiences were more reluctant to use NRT. No trials on e-cigarette use in pregnancy were included in the Cochrane effectiveness review,​ so we considered only NRT findings when integrating results from this review and the effectiveness review. No qualitative studies were conducted alongside trials,​ making full integration of the findings challenging. Women enrolled in trials would have agreed to being allocated to NRT or control group and would have received standardised information on NRT at the start of the trial. Overall,​ the findings of this synthesis are less relevant to women's decisions about starting NRT in trials and more likely to help explain trial participants' adherence to NRT after starting . We considered most findings to be of moderate certainty; we assessed findings on NRT use as being of higher certainty than those on e-cigarette use. This was mainly due to the limited data from fewer studies (only in the UK and USA) that contributed to e-cigarette findings. Overall,​ we judged studies to be of acceptable quality with only minor methodological issues.,​ AUTHORS' CONCLUSIONS: Consistent messages from health professionals,​ based on high-quality evidence and clearly explaining the safety of NRT and e-cigarettes compared to smoking in pregnancy,​ could help women use NRT and e-cigarettes more consistently/as recommended. This may improve their attitudes towards NRT or e-cigarettes,​ increase their willingness to use these in their attempt to quit,​ and subsequently encourage them to stay smoke-free. Copyright © 2020 The Cochrane Collaboration. Published by John Wiley & Sons,​ Ltd. |
| 289 | Meernik,​ Clare,​ Baker,​ et al (2019) Impact of non-menthol flavours in e-cigarettes on perceptions and use: an updated systematic review. BMJ open; 9.  Keywords:   Abstract: OBJECTIVES: Given the exponential increase in the use of e-cigarettes among younger age groups and in the growth in research on e-cigarette flavours,​ we conducted a systematic review examining the impact of non-menthol flavoured e-cigarettes on e-cigarette perceptions and use among youth and adults.,​ DESIGN: PubMed,​ Embase,​ PyscINFO and CINAHL were systematically searched for studies published and indexed through March 2018.,​ ELIGIBILITY CRITERIA: Quantitative observational and experimental studies that assessed the effect of non-menthol flavours in e-cigarettes on perceptions and use behaviours were included. Specific outcome measures assessed are appeal,​ reasons for use,​ risk perceptions,​ susceptibility,​ intention to try,​ initiation,​ preference,​ current use,​ quit intentions and cessation.,​ DATA EXTRACTION AND SYNTHESIS: Three authors independently extracted data related to the impact of flavours in tobacco products. Data from a previous review were then combined with those from the updated review for final analysis. Results were then grouped and analysed by outcome measure.,​ RESULTS: The review included 51 articles for synthesis,​ including 17 published up to 2016 and an additional 34 published between 2016 and 2018. Results indicate that non-menthol flavours in e-cigarettes decrease harm perceptions (five studies) and increase willingness to try and initiation of e-cigarettes (six studies). Among adults,​ e-cigarette flavours increase product appeal (seven studies) and are a primary reason many adults use the product (five studies). The role of flavoured e-cigarettes on smoking cessation remains unclear (six studies).,​ CONCLUSION: This review provides summary data on the role of non-menthol flavours in e-cigarette perceptions and use. Consistent evidence shows that flavours attract both youth and adults to use e-cigarettes. Given the clear findings that such flavours increase product appeal,​ willingness to try and initiation among youth,​ banning non-menthol flavours in e-cigarettes may reduce youth e-cigarette use. Longitudinal research is needed to examine any role flavours may play in quit behaviours among adults. Copyright © Author(s) (or their employer(s)) 2019. Re-use permitted under CC BY-NC. No commercial re-use. See rights and permissions. Published by BMJ. |
| 293 | Aladeokin,​ Adewale,​ Haighton,​ et al (2019) Is adolescent e-cigarette use associated with smoking in the United Kingdom?: A systematic review with meta-analysis. Tobacco prevention & cessation; 5.  Keywords:   Abstract: INTRODUCTION: Though smoking is a public health problem the use of e-cigarettes has been associated with a reduction in smoking in developed countries. However,​ public health experts have raised concerns about the association of e-cigarette use with an increase in traditional cigarette smoking in adolescents. Review-level evidence is generally supportive of this concern,​ but as it is mainly based on studies from the USA we investigated if e-cigarette use is associated with traditional cigarette smoking in adolescents (aged 10-19 years) in the UK.,​ METHODS: We conducted a systematic review of empirical studies. Databases (PubMed,​ Medline via ProQuest,​ CINAHL and SCOPUS) were searched between January 2005 and May 2018 using search terms based on the concepts: adolescents,​ traditional cigarette smoking,​ e-cigarettes,​ and UK. Using pre-defined inclusion and exclusion criteria,​ a total of eight studies (involving 73076 adolescents) were included in this review. Three of the included studies were eligible to be combined in a meta-analysis. The CASP appraisal tool was used to assess study quality while risk of bias was assessed using ROBINS-I.,​ RESULTS: Studies included in the meta-analysis showed that adolescents who use e-cigarettes are up to six times more likely to smoke traditional cigarettes. Furthermore,​ results showed that traditional cigarette smoking can also precede e-cigarette use in adolescents and there was increased likelihood of an increase in initial product use (e-cigarette or traditional cigarette) when the alternate product was initiated.,​ CONCLUSIONS: Public health policy makers in the UK still need clear conclusions about the effects and safety of e-cigarettes. Copyright © 2019 Aladeokin A. |
| 295 | Khouja,​ JasmineN,​ Suddell,​ et al (2020) Is e-cigarette use in non-smoking young adults associated with later smoking? A systematic review and meta-analysis. Tobacco control; .  Keywords:   Abstract: OBJECTIVE: The aim of this review was to investigate whether e-cigarette use compared with non-use in young non-smokers is associated with subsequent cigarette smoking.,​ DATA SOURCES: PubMed,​ Embase,​ Web of Science,​ Wiley Cochrane Library databases,​ and the 2018 Society for Research on Nicotine and Tobacco and Society for Behavioural Medicine conference abstracts.,​ STUDY SELECTION: All studies of young people (up to age 30 years) with a measure of e-cigarette use prior to smoking and an outcome measure of smoking where an OR could be calculated were included (excluding reviews and animal studies).,​ DATA EXTRACTION: Independent extraction was completed by multiple authors using a preprepared extraction form.,​ DATA SYNTHESIS: Of 9199 results,​ 17 studies were included in the meta-analysis. There was strong evidence for an association between e-cigarette use among non-smokers and later smoking (OR: 4.59,​ 95% CI: 3.60 to 5.85) when the results were meta-analysed in a random-effects model. However,​ there was high heterogeneity (I2 =88%).,​ CONCLUSIONS: Although the association between e-cigarette use among non-smokers and subsequent smoking appears strong,​ the available evidence is limited by the reliance on self-report measures of smoking history without biochemical verification. None of the studies included negative controls which would provide stronger evidence for whether the association may be causal. Much of the evidence also failed to consider the nicotine content of e-liquids used by non-smokers meaning it is difficult to make conclusions about whether nicotine is the mechanism driving this association. Copyright © Author(s) (or their employer(s)) 2020. Re-use permitted under CC BY. Published by BMJ. |
| 300 | Huang,​ J (2019) MS15.04 Approaching Cessation in the Patient Using Electronic Cigarettes. Journal of Thoracic Oncology; 14.  Keywords: adult,​attention,​berry,​carpenter,​Cinahl,​cohort analysis,​combustion,​controlled study,​counseling,​current smoker,​data extraction,​Embase,​female,​flavor,​follow up,​harm reduction,​health hazard,​human,​juvenile,​male,​Medline,​motivation,​nicotine replacement therapy  Abstract: Background: Electronic nicotine delivery systems (ENDS or e-cigarettes) have transformed the US tobacco market since mid-2000s'. Today,​ ENDS are the most-used tobacco products among American youth,​ and over two thirds of adult smokers have tried or are currently using ENDS. The role of ENDS in public health has been heatedly debated in recent years. While ENDS proponents argue for their potential in tobacco harm reduction by obsoleting combusted tobacco,​ the most dangerous form of tobacco,​ the sceptics are concerned about the uncertainty of long-term health risks of ENDS use and their role in increasing nicotine initiation among youth,​ which may act as a gateway to other forms of more harmful tobacco products and other substance abuses. The two key assumptions underlying the tobacco harm reduction argument are 1) the health risks associated with ENDS use are substantially less than that with combusted tobacco,​ and 2) ENDS use will lead to significant higher cessation rates compared with non-ENDS use among combusted tobacco users. This study examines the evidence related to the second assumption on the role of ENDS in increasing population level smoking cessation rate. The number of studies that examine whether and to what extent ENDS use increases smoking cessation rates is growing rapidly in recent years,​ however,​ the results from these studies are often mixed,​ with some studies showing ENDS use may increase smoking cessation rates compared with FDA-approved nicotine replacement therapy (NRT) products,​ while other studies showing no effect of ENDS use in increasing successfully quit rates. The goal of this study is to review and summarize these studies,​ paying particular attention to study methods,​ factors that may influence ENDS use,​ such as use frequency,​ device types,​ reasons for use,​ and flavors,​ and make recommendations on how health professionals should advise smokers about the role of ENDS in smoking cessation. Method(s): We conducted searches of MEDLINE,​ EMBASE,​ PsycInfo,​ CINAHL,​ CENTRAL and Web of Science up to May,​ 31 2019 using the combination of keywords "ENDS,​ electronic nicotine delivery systems,​ e-cigarette(s),​ electronic cigarette(s),​ vaping,​ electronic vaping product,​" and "cessation,​ smoking cessation,​ quitting." Our study selection criteria include randomized controlled trials (RCTs) in which current smokers (motivated or unmotivated to quit) were randomized to different conditions (not necessarily based on ENDS use),​ and which measured abstinence rates at three months or longer. We also included prospective cohort follow-up studies with at least three months follow-up. We followed standard systematic review methods for screening and data extraction,​ with two reviewers independently screened potentially eligible articles,​ extracted data from included studies on populations,​ methods,​ interventions,​ and outcomes and assessed their risk of bias. Our main outcome measure was abstinence from smoking after at least three months follow-up,​ and the outcome include both biochemically validated and self-reported smoking abstinence. Result(s): We identified seven RCTs and 22 prospective cohort follow-up studies that satisfy our study selection criteria. Among the seven RCTs,​ three RCT showed favorable outcomes for ENDS use. One RCT (Hajek et al.,​ 2019) found that ENDS use increased smoking cessation rates compared with NRT products (1-year abstinence rate 18.0% vs 9.9%,​ relative risk,​ 1.83; 95% CI,​ 1.30 to 2.58; P<0.001),​ another RCT (Masiero et al.,​ 2019) found that smokers used ENDS had higher smoking cessation rate compared with the control group (3-month abstinence rate 25% vs 10%),​ and a third RCT (Caponnetto et al.,​ 2014) found smoking cessation rate at 52-week follow up was higher among smokers who used ENDS compared with those who use no-nicotine ENDS. Two RCTs (Bullen et al.,​ 2103; Carpenter et al.,​ 2017) found that cessation behaviors (quit attempts,​ abstinence) were numerically but not statistically favored ENDS participants,​ and two RCTs (Halpern et al.,​ 2018; Adriaens et al.,​ 2014) found that |
| 307 | Claire,​ Ravinder,​ Chamberlain,​ et al (2020) Pharmacological interventions for promoting smoking cessation during pregnancy. The Cochrane database of systematic reviews; 3.  Keywords:   Abstract: BACKGROUND: Tobacco smoking in pregnancy causes serious health problems for the developing fetus and mother. When used by non-pregnant smokers,​ pharmacotherapies (nicotine replacement therapy (NRT),​ bupropion,​ and varenicline) are effective for increasing smoking cessation,​ however their efficacy and safety in pregnancy remains unknown. Electronic cigarettes (ECs) are becoming widely used,​ but their efficacy and safety when used for smoking cessation in pregnancy are also unknown.,​ OBJECTIVES: To determine the efficacy and safety of smoking cessation pharmacotherapies and ECs used during pregnancy for smoking cessation in later pregnancy and after childbirth,​ and to determine adherence to smoking cessation pharmacotherapies and ECs for smoking cessation during pregnancy.,​ SEARCH METHODS: We searched the Cochrane Pregnancy and Childbirth Group's Trials Register (20 May 2019),​ trial registers,​ and grey literature,​ and checked references of retrieved studies.,​ SELECTION CRITERIA: Randomised controlled trials (RCTs) conducted in pregnant women,​ comparing smoking cessation pharmacotherapy or EC use with either placebo or no pharmacotherapy/EC control. We excluded quasi-randomised,​ cross-over,​ and within-participant designs,​ and RCTs with additional intervention components not matched between trial arms.,​ DATA COLLECTION AND ANALYSIS: We followed standard Cochrane methods. The primary efficacy outcome was smoking cessation in later pregnancy; safety was assessed by 11 outcomes (principally birth outcomes) that indicated neonatal and infant well-being. We also collated data on adherence to trial treatments. We calculated the risk ratio (RR) or mean difference (MD) and the 95% confidence intervals (CI) for each outcome for each study,​ where possible. We grouped eligible studies according to the type of comparison. We carried out meta-analyses where appropriate.,​ MAIN RESULTS: We included 11 trials that enrolled a total of 2412 pregnant women who smoked at enrolment,​ nine trials of NRT and two trials of bupropion as adjuncts to behavioural support,​ with comparable behavioural support provided in the control arms. No trials investigated varenicline or ECs. We assessed four trials as at low risk of bias overall. The overall certainty of the evidence was low across outcomes and comparisons as assessed using GRADE,​ with reductions in confidence due to risk of bias,​ imprecision,​ and inconsistency. Compared to placebo and non-placebo (behavioural support only) controls,​ there was low-certainty evidence that NRT increased the likelihood of smoking abstinence in later pregnancy (RR 1.37,​ 95% CI 1.08 to 1.74; I2 = 34%,​ 9 studies,​ 2336 women). However,​ in subgroup analysis by comparator type,​ there was a subgroup difference between placebo-controlled and non-placebo controlled RCTs (test for subgroup differences P = 0.008). There was unclear evidence of an effect in placebo-controlled RCTs (RR 1.21,​ 95% CI 0.95 to 1.55; I2 = 0%,​ 6 studies,​ 2063 women),​ whereas non-placebo-controlled trials showed clearer evidence of a benefit (RR 8.55,​ 95% CI 2.05 to 35.71; I2 = 0%,​ 3 studies,​ 273 women). An additional subgroup analysis in which studies were grouped by the type of NRT used found no difference in the effectiveness of NRT in those using patches or fast-acting NRT (test for subgroup differences P = 0.08). There was no evidence of a difference between NRT and control groups in rates of miscarriage,​ stillbirth,​ premature birth,​ birthweight,​ low birthweight,​ admissions to neonatal intensive care,​ caesarean section,​ congenital abnormalities,​ or neonatal death. In one study infants born to women who had been randomised to NRT had higher rates of 'survival without developmental impairment' at two years of age compared to the placebo group. Non-serious adverse effects observed with NRT included headache,​ nausea,​ and local reactions (e.g. skin irritation from patches or foul taste from gum),​ but data could not be pooled. Adherence to NRT treatment regimens was generally low. We identified low-certainty evidence that there was no difference in s oking abstinence rates observed in later pregnancy in women using bupropion when compared to placebo control (RR 0.74,​ 95% CI 0.21 to 2.64; I2 = 0%,​ 2 studies,​ 76 women). Evidence investigating the safety outcomes of bupropion use was sparse,​ but the existing evidence showed no difference between the bupropion and control group.,​ AUTHORS' CONCLUSIONS: NRT used for smoking cessation in pregnancy may increase smoking cessation rates in late pregnancy. However,​ this evidence is of low certainty,​ as the effect was not evident when potentially biased,​ non-placebo-controlled RCTs were excluded from the analysis. Future studies may therefore change this conclusion. We found no evidence that NRT has either positive or negative impacts on birth outcomes; however,​ the evidence for some of these outcomes was also judged to be of low certainty due to imprecision and inconsistency. We found no evidence that bupropion may be an effective aid for smoking cessation during pregnancy,​ and there was little evidence evaluating its safety in this population. Further research evidence on the efficacy and safety of pharmacotherapy and EC use for smoking cessation in pregnancy is needed,​ ideally from placebo-controlled RCTs that achieve higher adherence rates and that monitor infants' outcomes into childhood. Future RCTs of NRT should investigate higher doses than those tested in the studies included in this review. Copyright © 2020 The Cochrane Collaboration. Published by John Wiley & Sons,​ Ltd. |
| 311 | Us Preventive Services Task Force,​ Owens,​ Douglas K,​ et al (2020) Primary Care Interventions for Prevention and Cessation of Tobacco Use in Children and Adolescents: US Preventive Services Task Force Recommendation Statement. JAMA; 323.  Keywords: Adolescent,​*Behavior Therapy/mt [Methods],​Child,​*Counseling,​Humans,​*Patient Education as Topic,​*Primary Health Care,​*Smoking Cessation/mt [Methods],​*Smoking Prevention/mt [Methods],​*Tobacco Use/pc [Prevention & Control],​Vaping/pc [Prevention & Control]  Abstract: Importance: Tobacco use is the leading cause of preventable death in the US. An estimated annual 480000 deaths are attributable to tobacco use in adults,​ including from secondhand smoke. It is estimated that every day about 1600 youth aged 12 to 17 years smoke their first cigarette and that about 5.6 million adolescents alive today will die prematurely from a smoking-related illness. Although conventional cigarette use has gradually declined among children in the US since the late 1990s,​ tobacco use via electronic cigarettes (e-cigarettes) is quickly rising and is now more common among youth than cigarette smoking. e-Cigarette products usually contain nicotine,​ which is addictive,​ raising concerns about e-cigarette use and nicotine addiction in children. Exposure to nicotine during adolescence can harm the developing brain,​ which may affect brain function and cognition,​ attention,​ and mood; thus,​ minimizing nicotine exposure from any tobacco product in youth is important.,​ Objective: To update its 2013 recommendation,​ the USPSTF commissioned a review of the evidence on the benefits and harms of primary care interventions for tobacco use prevention and cessation in children and adolescents. The current systematic review newly included e-cigarettes as a tobacco product.,​ Population: This recommendation applies to school-aged children and adolescents younger than 18 years.,​ Evidence Assessment: The USPSTF concludes with moderate certainty that primary care-feasible behavioral interventions,​ including education or brief counseling,​ to prevent tobacco use in school-aged children and adolescents have a moderate net benefit. The USPSTF concludes that there is insufficient evidence to determine the balance of benefits and harms of primary care interventions for tobacco cessation among school-aged children and adolescents who already smoke,​ because of a lack of adequately powered studies on behavioral counseling interventions and a lack of studies on medications.,​ Recommendation: The USPSTF recommends that primary care clinicians provide interventions,​ including education or brief counseling,​ to prevent initiation of tobacco use among school-aged children and adolescents. (B recommendation) The USPSTF concludes that the current evidence is insufficient to assess the balance of benefits and harms of primary care-feasible interventions for the cessation of tobacco use among school-aged children and adolescents. (I statement). |
| 312 | Selph,​ Shelley,​ Patnode,​ et al (2020) Primary Care-Relevant Interventions for Tobacco and Nicotine Use Prevention and Cessation in Children and Adolescents: Updated Evidence Report and Systematic Review for the US Preventive Services Task Force. JAMA; 323.  Keywords: Adolescent,​Adult,​Behavior Therapy/mt [Methods],​*Behavior Therapy,​Child,​Counseling,​Humans,​*Patient Education as Topic,​Practice Guidelines as Topic,​*Primary Health Care,​*Smoking Cessation/mt [Methods],​*Smoking Prevention/mt [Methods],​*Tobacco Use/pc [Prev  Abstract: Importance: Interventions to discourage the use of tobacco products (including electronic nicotine delivery systems or e-cigarettes) among children and adolescents may help decrease tobacco-related illness and injury.,​ Objective: To update the 2013 review on primary care-relevant interventions for tobacco use prevention and cessation in children and adolescents to inform the US Preventive Services Task Force.,​ Data Sources: The Cochrane Central Register of Controlled Trials and Cochrane Database of Systematic Reviews,​ MEDLINE,​ PsyINFO,​ and EMBASE (September 1,​ 2012,​ to June 25,​ 2019),​ with surveillance through February 7,​ 2020.,​ Study Selection: Primary care-relevant studies; randomized clinical trials and nonrandomized controlled intervention studies of children and adolescents up to age 18 years for cessation and age 25 years for prevention. Trials comparing behavioral or pharmacological interventions with no or a minimal tobacco use intervention control group (eg,​ usual care,​ attention control,​ wait list) were included.,​ Data Extraction and Synthesis: One investigator abstracted data and a second investigator checked data abstraction for accuracy. Two investigators independently assessed study quality. Studies were pooled using random-effects meta-analysis.,​ Main Outcomes and Measures: Tobacco use initiation; tobacco use cessation; health outcomes; harms.,​ Results: Twenty-four randomized clinical trials (N = 44521) met inclusion criteria. Behavioral interventions were associated with decreased likelihood of cigarette smoking initiation compared with control interventions at 7 to 36 months' follow-up (13 trials,​ n = 21700; 7.4% vs 9.2%; relative risk [RR],​ 0.82 [95% CI,​ 0.73-0.92]). There was no statistically significant difference between behavioral interventions and controls in smoking cessation when trials were restricted to smokers (9 trials,​ n = 2516; 80.7% vs 84.1% continued smoking; RR,​ 0.97 [95% CI,​ 0.93-1.01]). There were no significant benefits of medication on likelihood of smoking cessation in 2 trials of bupropion at 26 weeks (n = 523; 17% [300 mg] and 6% [150 mg] vs 10% [placebo]; 24% [150 mg] vs 28% [placebo]) and 1 trial of nicotine replacement therapy at 12 months (n = 257; 8.1% vs 8.2%). One trial each (n = 2586 and n = 1645) found no beneficial intervention effect on health outcomes or on adult smoking. No trials of prevention in young adults were identified. Few trials addressed prevention or cessation of tobacco products other than cigarettes; no trials evaluated effects of interventions on e-cigarette use. There were few trials of pharmacotherapy,​ and they had small sample sizes.,​ Conclusions and Relevance: Behavioral interventions may reduce the likelihood of smoking initiation in nonsmoking children and adolescents. Research is needed to identify effective behavioral interventions for adolescents who smoke cigarettes or who use other tobacco products and to understand the effectiveness of pharmacotherapy. |
| 323 | Doshi,​ P,​ Pineles,​ et al (2020) Systematic review of systematic reviews: Do ecigarettes affect smoking cessation?. Journal of Investigative Medicine; 68.  Keywords: A Measurement Tool to Assess Systematic Reviews,​adult,​conference abstract,​electronic cigarette,​epidemic,​female,​filtration,​harm reduction,​human,​male,​meta analysis,​patient care,​public health,​risk assessment,​risk factor,​*smoking cessation,​systematic review  Abstract: Purpose of study With the global tobacco epidemic accounting for 6 million premature deaths each year,​ there is ongoing urgency to reduce smoking. When e-cigarettes were introduced,​ unsubstantiated claims were made that they facilitated smoking cessation and harm reduction,​ which led to multiple research investigations since 2011. Although there is a paucity of high-quality evidence,​ systematic reviews have been published at a rapid rate. Given the billion potential market value by 2025,​ the weight of evidence for e-cigarettes in smoking cessation is critical. Methods used We searched several databased from inception up to August 2018 with key terms related to e-cigarettes and smoking cessation,​ filtering for systematic reviews/meta-analyses. Each included review was classified and ranked by study design using a validated instrument called the AMSTAR tool. Summary of results The original search yielded 522 unique studies,​ of which 11 met inclusion criteria,​ and 7 of which also included summary estimates from a meta-analysis. All except 2 studies received a 'Low' or 'Critically Low' AMSTAR quality score. The number of studies,​ as well as methodology,​ cited by each review varied widely from 4 to 19,​ of which only two studies were RCTs. The estimated relative risk of successful smoking cessation in e-cigarette users compared to non-users ranges from 0.61 to 2.29 in the seven meta-analyses,​ a range with qualitatively different implications at its extremes. Furthermore,​ The authors of the studies provided conclusions that varied widely,​ but nearly all agreed that the evidence was 'limited.' Conclusions Two findings from our analysis are striking: (1) the 11 systematic reviews covered substantially different primary studies,​ even for those with overlapping timespans for literature identification,​; and (2) the reviews reached different conclusions even if considering the same evidence. With inclusion of anywhere from 8% to 52% of available articles,​ none of the systematic reviews presented a comprehensive analysis of the literature. Our review shows that those making policy and patient care decisions need to approach the literature cautiously. For such a critical public health topic,​ we propose that new,​ carefully documented systematic reviews will be needed. |
| 331 | Lee,​ Stella Juhyun,​ Rees,​ et al (2020) Youth and Young Adult Use of Pod-Based Electronic Cigarettes From 2015 to 2019: A Systematic Review. JAMA pediatrics; .  Keywords:   Abstract: Importance: The use of electronic cigarettes (e-cigarettes) has rapidly increased among youth and young adults,​ but knowledge gaps exist on the potential health effects of using recently introduced pod-based e-cigarettes.,​ Objective: To conduct a systematic review of recent peer-reviewed scientific literature on pod-based e-cigarettes.,​ Evidence Review: A search of online databases,​ including PubMed,​ Web of Science,​ Embase,​ and EBSCO HOST,​ was conducted to identify pod-based e-cigarette-associated articles from June 2015 (the time when JUUL [JUUL Labs] was introduced) to June 2019. We included English-language articles that presented primary data on pod-based e-cigarettes.,​ Findings: Pod-based e-cigarettes represent a substantial evolution in design by increasing the efficient delivery of nicotine. While these products may contain less harmful constituents than other types of e-cigarettes and cigarettes,​ there is no evidence that the levels found are safe among youth. There is evidence for higher nicotine dependence associated with their use. Pod-based e-cigarette brands,​ compared with other e-cigarette brands,​ have targeted youth and young adults with social media marketing. There was less discussion about the use of these products as smoking cessation devices or their health risks on social media. The social acceptability and favorable perceptions of pod-based e-cigarettes may underlie the use of these products.,​ Conclusions and Relevance: The appeal and dependence potential of pod-based e-cigarettes for youth emphasize the need for stronger regulations on product design,​ social media,​ marketing channels,​ and youth access together with health communications that emphasize the risks of nicotine dependence. |

**APPENDIX 3: Full-text articles excluded (with reasons for exclusion).**

| **Result #** | **Reference** | **Exclusion Criteria** |
| --- | --- | --- |
| 1 | **Underner M.,Perriot J.,Brousse G.,de Chazeron I.,Schmitt A.,Peiffer G.,Harika-Germaneau G.,Jaafari N.**. Stopping and reducing smoking in patients with schizophrenia. *Encephale.* 2019. 45:345 | Articles in which the full text is non-English. |
| 12 | **Signes-Costa J.,de Granda-Orive J.I.,Ramos Pinedo A.,Camarasa Escrig A.,de Higes Martinez E.,Rabade Castedo C.,Cabrera Cesar E.,Jimenez-Ruiz C.A.**. Official Statement of the Spanish Society of Pulmonology and Thoracic Surgery (SEPAR) on Electronic Cigarettes and IQOS. *Archivos de Bronconeumologia.* 2019. #volume#: | Articles that are not systematic reviews with or without meta-analyses. |
| 13 | **Fadus M.C.,Smith T.T.,Squeglia L.M.**. The rise of e-cigarettes, pod mod devices, and JUUL among youth: Factors influencing use, health implications, and downstream effects. *Drug and Alcohol Dependence.* 2019. 201:85 | Articles that are not systematic reviews with or without meta-analyses. |
| 14 | **Heiss C.**. Electronic cigarettes: Replacing one evil with another?. *European Journal of Preventive Cardiology.* 2019. 26:1217 | Articles that are not systematic reviews with or without meta-analyses. |
| 15 | **Wallace A.M.,Foronjy R.E.**. Electronic cigarettes: Not evidence-based cessation. *Translational Lung Cancer Research.* 2019. 8:S7 | Articles that are not systematic reviews with or without meta-analyses. |
| 16 | **Villanti A.C.,Feirman S.P.,Niaura R.S.,Pearson J.L.,Glasser A.M.,Collins L.K.,Abrams D.B.**. How do we determine the impact of e-cigarettes on cigarette smoking cessation or reduction? Review and recommendations for answering the research question with scientific rigor. *Addiction (Abingdon, England).* 2018. 113:391 | Articles that do not assess the associations between e-cigarette use and cigarette smoking cessation or initiation. |
| 21 | **Krusemann E.,Boesveldt S.,De Graaf K.,Talhout R.**. An overview of the role of flavors in e-cigarette addiction. *Tobacco Induced Diseases.* 2018. 16:84 | Articles that are not systematic reviews with or without meta-analyses. |
| 32 | **Livingston C.J.,Freeman R.J.,Costales V.C.,Westhoff J.L.,Caplan L.S.,Sherin K.M.,Niebuhr D.W.**. Electronic Nicotine Delivery Systems or E-cigarettes: American College of Preventive Medicine's Practice Statement. *American Journal of Preventive Medicine.* 2019. 56:167 | Articles that are not systematic reviews with or without meta-analyses. |
| 33 | **Albano C.,Yang F.,Buckley B.,Williams L.**. A systematic review on the health and safety of electronic cigarettes. *Journal of Managed Care and Specialty Pharmacy.* 2016. 22:S88 | Articles that are not systematic reviews with or without meta-analyses. |
| 36 | **Mayel M.**. Are vapes an effective device for smoking cessation or a gateway to conventional tobacco smoking?. *Canadian Journal of Respiratory Therapy.* 2018. 54:55 | Articles that are not systematic reviews with or without meta-analyses. |
| 40 | **Hamee R.H.**. Human health effects of electronic cigarettes: A review. *Indian Journal of Public Health Research and Development.* 2018. 9:1387 | Articles that do not assess the associations between e-cigarette use and cigarette smoking cessation or initiation. |
| 41 | **Kaur G.,Pinkston R.,McLemore B.,Dorsey W.C.,Batra S.**. Immunological and toxicological risk assessment of e-cigarettes. *European Respiratory Review.* 2018. 27:170119 | Articles that do not assess the associations between e-cigarette use and cigarette smoking cessation or initiation. |
| 42 | **Ghamri R.A.**. Identification of the most effective pharmaceutical products for smoking cessation: A literature review. *Journal of Substance Use.* 2018. 23:670 | Articles that do not assess the associations between e-cigarette use and cigarette smoking cessation or initiation. |
| 48 | **Chatterjee K.,Alzghoul B.,Innabi A.,Meena N.**. Is vaping a gateway to smoking: A review of the longitudinal studies. *International Journal of Adolescent Medicine and Health.* 2018. 30:20160033 | Articles that are not systematic reviews with or without meta-analyses. |
| 51 | **Onor I.O.,Stirling D.L.,Williams S.R.,Bediako D.,Borghol A.,Harris M.B.,Darensburg T.B.,Clay S.D.,Okpechi S.C.,Sarpong D.F.**. Clinical effects of cigarette smoking: Epidemiologic impact and review of pharmacotherapy options. *International Journal of Environmental Research and Public Health.* 2017. 14:1147 | Articles that are not systematic reviews with or without meta-analyses. |
| 53 | **Soneji S.,Primack B.A.,Pierce J.P.,Sung H.-Y.,Sargent J.D.**. Re: Modeling the effects of e-cigarettes on smoking behavior: Implications for future adult smoking prevalence. *Epidemiology.* 2017. 28:e1 | Articles that are not systematic reviews with or without meta-analyses. |
| 56 | **Gage S.H.,Maynard O.M.**. Smoke-free policies in psychiatric hospitals need resources. *The Lancet Psychiatry.* 2017. 4:509 | Articles that are not systematic reviews with or without meta-analyses. |
| 58 | **Orellana-Barrios M.A.,Payne D.,Medrano-Juarez R.M.,Yang S.,Nugent K.**. Electronic Cigarettes for Smoking Cessation. *American Journal of the Medical Sciences.* 2016. 352:420 | Articles that are not systematic reviews with or without meta-analyses. |
| 59 | **Orellana-Barrios M.A.,Payne D.,Nugent K.**. E-cigarettes and smoking cessation. *The Lancet Respiratory Medicine.* 2016. 4:e24 | Articles that are not systematic reviews with or without meta-analyses. |
| 60 | **Greenhill R.,Dawkins L.,Notley C.,Finn M.D.,Turner J.J.D.**. Adolescent Awareness and Use of Electronic Cigarettes: A Review of Emerging Trends and Findings. *Journal of Adolescent Health.* 2016. 59:612 | Articles that do not assess the associations between e-cigarette use and cigarette smoking cessation or initiation. |
| 62 | **Cobb N.K.,Sonti R.**. E-cigarettes: The science behind the smoke and mirrors. *Respiratory Care.* 2016. 61:1122 | Articles that are not systematic reviews with or without meta-analyses. |
| 64 | **Tuan R.S.**. Prenatal substance use and developmental disorders: Overview and highlights. *Birth Defects Research Part C - Embryo Today: Reviews.* 2016. 108:106 | Articles that are not systematic reviews with or without meta-analyses. |
| 65 | **Printz C.**. UCSF study: E-cigarettes are not helping smokers quit. *Cancer.* 2016. 122:1636 | Articles that are not systematic reviews with or without meta-analyses. |
| 66 | **Feirman S.P.,Lock D.,Cohen J.E.,Holtgrave D.R.,Li T.**. Flavored Tobacco Products in the United States: A Systematic Review Assessing Use and Attitudes. *Nicotine and Tobacco Research.* 2016. 18:739 | Articles that do not assess the associations between e-cigarette use and cigarette smoking cessation or initiation. |
| 67 | **Jhanjee S.**. Putting tobacco harm reduction in perspective: Is it a viable alternative?. *Indian Journal of Medical Research.* 2016. 143:25 | Articles that are not systematic reviews with or without meta-analyses. |
| 68 | **Ioakeimidis N.,Vlachopoulos C.,Tousoulis D.**. Efficacy and safety of electronic cigarettes for smoking cessation: A critical approach. *Hellenic Journal of Cardiology.* 2016. 57:1 | Articles that are not systematic reviews with or without meta-analyses. |
| 69 | **Bernstein S.L.**. Electronic cigarettes: More light, less heat needed. *The Lancet Respiratory Medicine.* 2016. 4:85 | Articles that are not systematic reviews with or without meta-analyses. |
| 71 | **Vanderkam P.,Boussageon R.,Underner M.,Langbourg N.,Brabant Y.,Binder P.,Freche B.,Jaafari N.**. Efficacy and security of electronic cigarette for tobacco harm reduction: Systematic review and meta-analysis. *Presse Medicale.* 2016. 45:971 | Articles in which the full text is non-English. |
| 72 | **David T.,Tharyan P.**. Electronic cigarettes for smoking cessation and reduction. Summary of the evidence and implications for public health programmes. *Clinical Epidemiology and Global Health.* 2016. 4:146 | Articles that are not systematic reviews with or without meta-analyses. |
| 73 | **Lindson-Hawley N.,Hartmann-Boyce J.,Fanshawe T.R.,Begh R.,Farley A.,Lancaster T.**. Interventions to reduce harm from continued tobacco use. *Cochrane Database of Systematic Reviews.* 2016. 2016:CD005231 | Articles that do not assess the associations between e-cigarette use and cigarette smoking cessation or initiation. |
| 77 | **The Lancet Psychiatry**. Ceci n'est pas une cigarette. *The Lancet Psychiatry.* 2015. 2:1043 | Articles that are not systematic reviews with or without meta-analyses. |
| 78 | **Meernik C.,Goldstein A.O.**. A critical review of smoking, cessation, relapse and emerging research in pregnancy and post-partum. *British Medical Bulletin.* 2015. 114:135 | Articles that are not systematic reviews with or without meta-analyses. |
| 81 | **Anonymous.**. Electronic nicotine delivery systems. *Pediatrics.* 2015. 136:1018 | Articles that are not systematic reviews with or without meta-analyses. |
| 83 | **Abrams D.B.,Niaura R.**. The importance of science-informed policy and what the data really tell us about e-cigarettes. *Israel Journal of Health Policy Research.* 2015. 4:22 | Articles that are not systematic reviews with or without meta-analyses. |
| 84 | **Waghel R.C.,Battise D.M.,Ducker M.L.**. Effectiveness of electronic cigarettes as a tool for smoking cessation or reduction. *Journal of Pharmacy Technology.* 2015. 31:8 | Articles that are not systematic reviews with or without meta-analyses. |
| 85 | **Brown J.**. A positive view on e-cigarettes. *BMJ (Online).* 2015. 351:h3864 | Articles that are not systematic reviews with or without meta-analyses. |
| 88 | **Lee A.H.Y.,Stater B.J.,Close L.,Rahmati R.**. Are e-cigarettes effective in smoking cessation?. *Laryngoscope.* 2015. 125:785 | Articles that are not systematic reviews with or without meta-analyses. |
| 89 | **Born H.,Persky M.,Kraus D.H.,Peng R.,Amin M.R.,Branski R.C.**. Electronic cigarettes: A primer for clinicians. *Otolaryngology - Head and Neck Surgery (United States).* 2015. 153:5 | Articles that are not systematic reviews with or without meta-analyses. |
| 90 | **Annamalai A.,Singh N.,O'Malley S.S.**. Smoking use and cessation among people with serious mental illness. *Yale Journal of Biology and Medicine.* 2015. 88:271 | Articles that are not systematic reviews with or without meta-analyses. |
| 91 | **Polosa R.,Caponnetto P.**. What to advise to respiratory patients intending to use electronic cigarettes. *Discovery Medicine.* 2015. 20:155 | Articles that are not systematic reviews with or without meta-analyses. |
| 93 | **Brose L.S.**. E-cigarettes may help smokers stop or reduce smoking. *Evidence-Based Medicine.* 2015. 20:134 | Articles that are not systematic reviews with or without meta-analyses. |
| 94 | **Begh R.,Lindson-Hawley N.,Aveyard P.**. Does reduced smoking if you can't stop make any difference?. *BMC Medicine.* 2015. 13:257 | Articles that are not systematic reviews with or without meta-analyses. |
| 95 | **Stubbs B.,Vancampfort D.,Bobes J.,De Hert M.,Mitchell A.J.**. How can we promote smoking cessation in people with schizophrenia in practice? A clinical overview. *Acta Psychiatrica Scandinavica.* 2015. 132:122 | Articles that do not assess the associations between e-cigarette use and cigarette smoking cessation or initiation. |
| 97 | **Foley N.C.,Lindsay P.**. The use of electronic cigarettes is not associated with cessation of smoking: A systematic review and meta-analysis. *International Journal of Stroke.* 2015. 10:64 | Articles that are not systematic reviews with or without meta-analyses. |
| 98 | **Allehebi R.O.,Khan M.,Stanbrook M.B.**. Efficacy and safety of electronic cigarettes for smoking cessation: A systematic review. *American Journal of Respiratory and Critical Care Medicine.* 2015. 191: | Articles that are not systematic reviews with or without meta-analyses. |
| 100 | **Meo S.A.,Al Asiri S.A.**. Effects of electronic cigarette smoking on human health. *European Review for Medical and Pharmacological Sciences.* 2014. 18:3315 | Articles that do not assess the associations between e-cigarette use and cigarette smoking cessation or initiation. |
| 101 | **Prochaska J.J.**. Quitting smoking is associated with long term improvements in mood. *BMJ (Online).* 2014. 348:g1562 | Articles that are not systematic reviews with or without meta-analyses. |
| 102 | **Rahman M.A.,Hann N.,Wilson A.,Worrall-Carter L.**. Electronic cigarettes: Patterns of use, health effects, use in smoking cessation and regulatory issues. *Tobacco Induced Diseases.* 2014. 12:21 | Articles that are not systematic reviews with or without meta-analyses. |
| 104 | **Ratschen E.**. Electronic cigarettes in mental health settings - Solving a conundrum?. *Psychiatric Bulletin.* 2014. 38:226 | Articles that are not systematic reviews with or without meta-analyses. |
| 105 | **Chapman S.**. E-cigarettes: The best and the worst case scenarios for public health - An essay by Simon Chapman. *BMJ (Online).* 2014. 349:g5512 | Articles that are not systematic reviews with or without meta-analyses. |
| 106 | **Rahman M.A.,Hann N.R.,Wilson A.M.,Mnatzaganian G.,Worrall-Carter L.**. Electronic cigarettes are effective for smoking cessation: Evidence from a systematic review and meta-analysis. *Circulation.* 2014. 130: | Articles that are not systematic reviews with or without meta-analyses. |
| 109 | **Pratt A.,Su L.,Audera-Lopez C.,Rarick J.,Mercado S.**. The rise of e-cigarettes: An emerging threat to the tobacco endgame?. *Respiratory Medicine.* 2013. 107:S9 | Articles that are not systematic reviews with or without meta-analyses. |
| 111 | **Tahiri M.,Mottillo S.,Joseph L.,Pilote L.,Eisenberg M.J.**. Unconventional smoking cessation aids: A metaanalysis of randomized controlled trials. *American Journal of Epidemiology.* 2011. 173:S141 | Articles that are not systematic reviews with or without meta-analyses. |
| 119 | **Carrie D Patnode, Jillian T Henderson, Jamie H Thompson, Caitlyn A Senger, Stephen P Fortmann, Evelyn P Whitlock**. . *#journal#.* 2015. #volume#: | Articles that do not assess the associations between e-cigarette use and cigarette smoking cessation or initiation. |
| 124 | **Sarah Wolf, Senushi O'Sullivan, Roselynn Dean, Tomas Owens**. Does utilization of electronic cigarettes facilitate smoking cessation compared to other interventions?.. *The Journal of the Oklahoma State Medical Association.* 2019. 112:34 | Articles that are not systematic reviews with or without meta-analyses. |
| 125 | **Caitlin Notley, Tracey J Brown, Linda Bauld, Wendy Hardeman, Richard Holland, Felix Naughton, Sophie Orton, Michael Ussher**. Development of a Complex Intervention for the Maintenance of Postpartum Smoking Abstinence: Process for Defining Evidence-Based Intervention.. *International journal of environmental research and public health.* 2019. 16: | Articles that are not systematic reviews with or without meta-analyses. |
| 127 | **Michael D Klein, Natasha A Sokol, Laura R Stroud**. Electronic Cigarettes: Common Questions and Answers.. *American family physician.* 2019. 100:227 | Articles that are not systematic reviews with or without meta-analyses. |
| 133 | **Robert West, Kathryn Coyle, Lesley Owen, Doug Coyle, Subhash Pokhrel,EQUIPT Study Group**. Estimates of effectiveness and reach for 'return on investment' modelling of smoking cessation interventions using data from England.. *Addiction (Abingdon, England).* 2018. 113 Suppl 1:19 | Articles that do not assess the associations between e-cigarette use and cigarette smoking cessation or initiation. |
| 148 | **Caroline Franck, Kristian B Filion, Mark J Eisenberg**. Smoking Cessation in Patients With Acute Coronary Syndrome.. *The American journal of cardiology.* 2018. 121:1105 | Articles that do not assess the associations between e-cigarette use and cigarette smoking cessation or initiation. |
| 154 | **Tim Coleman, Catherine Chamberlain, Mary-Ann Davey, Sue E Cooper, Jo Leonardi-Bee**. Pharmacological interventions for promoting smoking cessation during pregnancy.. *The Cochrane database of systematic reviews.* 2015. #volume#:CD010078 | Articles that do not assess the associations between e-cigarette use and cigarette smoking cessation or initiation. |
| 161 | **Catherine Chamberlain, Susan Perlen, Sue Brennan, Lucie Rychetnik, David Thomas, Raglan Maddox, Noore Alam, Emily Banks, Andrew Wilson, Sandra Eades**. Evidence for a comprehensive approach to Aboriginal tobacco control to maintain the decline in smoking: an overview of reviews among Indigenous peoples.. *Systematic reviews.* 2017. 6:135 | Articles that do not assess the associations between e-cigarette use and cigarette smoking cessation or initiation. |
| 167 | **Samane Zare, Mehdi Nemati, Yuqing Zheng**. A systematic review of consumer preference for e-cigarette attributes: Flavor, nicotine strength, and type.. *PloS one.* 2018. 13:e0194145 | Articles that do not assess the associations between e-cigarette use and cigarette smoking cessation or initiation. |
| 176 | **Yanina Zborovskaya**. E-Cigarettes and Smoking Cessation: A Primer for Oncology Clinicians.. *Clinical journal of oncology nursing.* 2017. 21:54 | Articles that are not systematic reviews with or without meta-analyses. |
| 179 | **Allison M Glasser, Lauren Collins, Jennifer L Pearson, Haneen Abudayyeh, Raymond S Niaura, David B Abrams, Andrea C Villanti**. Overview of Electronic Nicotine Delivery Systems: A Systematic Review.. *American journal of preventive medicine.* 2017. 52:e33 | Articles that do not assess the associations between e-cigarette use and cigarette smoking cessation or initiation. |
| 180 | **Riccardo Polosa, Pasquale Caponnetto**. E-cigarettes and smoking cessation: a critique of a New England Journal Medicine-commissioned case study.. *Internal and emergency medicine.* 2017. 12:129 | Articles that are not systematic reviews with or without meta-analyses. |
| 182 | **Carlos Echevarria, Ian P Sinha**. Heterogeneity in the measurement and reporting of outcomes in studies of electronic cigarette use in adolescents: a systematic analysis of observational studies.. *Tobacco control.* 2017. 26:247 | Articles that do not assess the associations between e-cigarette use and cigarette smoking cessation or initiation. |
| 186 | **Gholamreza Heydari, Arezoo Ebn Ahmady, Fahimeh Chamyani, Mohammadreza Masjedi, Lida Fadaizadeh**. Electronic cigarette, effective or harmful for quitting smoking and respiratory health: A quantitative review papers.. *Lung India : official organ of Indian Chest Society.* 2017. 34:25 | Articles that are not systematic reviews with or without meta-analyses. |
| 189 | **Konstantinos E Farsalinos, Riccardo Polosa**. Safety evaluation and risk assessment of electronic cigarettes as tobacco cigarette substitutes: a systematic review.. *Therapeutic advances in drug safety.* 2014. 5:67 | Articles that do not assess the associations between e-cigarette use and cigarette smoking cessation or initiation. |
| 191 | **Brad Rodu**. The scientific foundation for tobacco harm reduction, 2006-2011.. *Harm reduction journal.* 2011. 8:19 | Articles that are not systematic reviews with or without meta-analyses. |
| 195 | **Jieming Zhong, Shuangshuang Cao, Weiwei Gong, Fangrong Fei, Meng Wang**. Electronic Cigarettes Use and Intention to Cigarette Smoking among Never-Smoking Adolescents and Young Adults: A Meta-Analysis.. *International journal of environmental research and public health.* 2016. 13: | Articles that are not systematic reviews with or without meta-analyses. |
| 201 | **Meng Wang, Jian-Wei Wang, Shuang-Shuang Cao, Hui-Qin Wang, Ru-Ying Hu**. Cigarette Smoking and Electronic Cigarettes Use: A Meta-Analysis.. *International journal of environmental research and public health.* 2016. 13: | Articles that do not assess the associations between e-cigarette use and cigarette smoking cessation or initiation. |
| 203 | **Charlotte Leduc, Elisabeth Quoix**. Is there a role for e-cigarettes in smoking cessation?.. *Therapeutic advances in respiratory disease.* 2016. 10:130 | Articles that are not systematic reviews with or without meta-analyses. |
| 207 | **Carrie D Patnode, Jillian T Henderson, Jamie H Thompson, Caitlyn A Senger, Stephen P Fortmann, Evelyn P Whitlock**. Behavioral Counseling and Pharmacotherapy Interventions for Tobacco Cessation in Adults, Including Pregnant Women: A Review of Reviews for the U.S. Preventive Services Task Force.. *Annals of internal medicine.* 2015. 163:608 | Articles that do not assess the associations between e-cigarette use and cigarette smoking cessation or initiation. |
| 217 | **Katherine Kelly Orr, Nicole J Asal**. Efficacy of electronic cigarettes for smoking cessation.. *The Annals of pharmacotherapy.* 2014. 48:1502 | Articles that are not systematic reviews with or without meta-analyses. |
| 218 | **Dennis Nowak, Rudolf A Jorres, Tobias Ruther**. E-cigarettes--prevention, pulmonary health, and addiction.. *Deutsches Arzteblatt international.* 2014. 111:349 | Articles that are not systematic reviews with or without meta-analyses. |
| 226 | **Brent Caldwell, Walt Sumner, Julian Crane**. A systematic review of nicotine by inhalation: is there a role for the inhaled route?.. *Nicotine & tobacco research : official journal of the Society for Research on Nicotine and Tobacco.* 2012. 14:1127 | Articles that do not assess the associations between e-cigarette use and cigarette smoking cessation or initiation. |
| 241 | **Theodore Lloyd**. Stubbing out smoking in schizophrenia.. *The Psychologist.* 2015. 28:32 | Articles that are not systematic reviews with or without meta-analyses. |
| 247 | **Selph, Shelley, Patnode, Carrie D., Bailey, Steffani R., Pappas, Miranda, Stoner, Ryan, Hart, Erica, Chou, Roger**. . *#journal#.* 2020. #volume#:#pages# | Articles that do not assess the associations between e-cigarette use and cigarette smoking cessation or initiation. |
| 264 | **Asher, Tim, Belden, Jenna Leah, Kelsberg, Gary, Safranek, Sarah**. Does using e-cigarettes increase cigarette smoking in adolescents?. *The Journal of family practice.* 2019. 68:E12 | Articles that are not systematic reviews with or without meta-analyses. |
| 271 | **Villalobos, R. E., Ambrocio, G. P. L., Fernandez, L.**. Electronic cigarettes for smoking cessation: An individual patient meta-analysis of randomized controlled trials. *European Respiratory Journal.* 2019. 54:#pages# | Articles that are not systematic reviews with or without meta-analyses. |
| 281 | **Campbell, Katarzyna, Coleman-Haynes, Thomas, Bowker, Katharine, Cooper, Sue E., Connelly, Sarah, Coleman, Tim**. Factors influencing the uptake and use of nicotine replacement therapy and e-cigarettes in pregnant women who smoke: a qualitative evidence synthesis. *The Cochrane database of systematic reviews.* 2020. 5:CD013629 | Articles that do not assess the associations between e-cigarette use and cigarette smoking cessation or initiation. |
| 300 | **Huang, J.**. MS15.04 Approaching Cessation in the Patient Using Electronic Cigarettes. *Journal of Thoracic Oncology.* 2019. 14:S186 | Articles that are not systematic reviews with or without meta-analyses. |
| 307 | **Claire, Ravinder, Chamberlain, Catherine, Davey, Mary-Ann, Cooper, Sue E., Berlin, Ivan, Leonardi-Bee, Jo, Coleman, Tim**. Pharmacological interventions for promoting smoking cessation during pregnancy. *The Cochrane database of systematic reviews.* 2020. 3:CD010078 | Articles that do not assess the associations between e-cigarette use and cigarette smoking cessation or initiation. |
| 311 | **Us Preventive Services Task Force, Owens, Douglas K, Davidson, Karina W, Krist, Alex H, Barry, Michael J, Cabana, Michael, Caughey, Aaron B, Curry, Susan J, Donahue, Katrina, Doubeni, Chyke A, Epling, John W Jr, Kubik, Martha, Ogedegbe, Gbenga, Pbert, Lori, Silverstein, Michael, Simon, Melissa A, Tseng, Chien-Wen, Wong**. Primary Care Interventions for Prevention and Cessation of Tobacco Use in Children and Adolescents: US Preventive Services Task Force Recommendation Statement. *JAMA.* 2020. 323:1590 | Articles that do not assess the associations between e-cigarette use and cigarette smoking cessation or initiation. |
| 312 | **Selph, Shelley, Patnode, Carrie, Bailey, Steffani R., Pappas, Miranda, Stoner, Ryan, Chou, Roger**. Primary Care-Relevant Interventions for Tobacco and Nicotine Use Prevention and Cessation in Children and Adolescents: Updated Evidence Report and Systematic Review for the US Preventive Services Task Force. *JAMA.* 2020. 323:1599 | Articles that do not assess the associations between e-cigarette use and cigarette smoking cessation or initiation. |
| 323 | **Doshi, P., Pineles, B., Buran, M., Samet, J.**. Systematic review of systematic reviews: Do ecigarettes affect smoking cessation?. *Journal of Investigative Medicine.* 2020. 68:706 | Articles that are not systematic reviews with or without meta-analyses. |
| 331 | **Lee, Stella Juhyun, Rees, Vaughan W., Yossefy, Noam, Emmons, Karen M., Tan, Andy S. L.**. Youth and Young Adult Use of Pod-Based Electronic Cigarettes From 2015 to 2019: A Systematic Review. *JAMA pediatrics.* 2020. #volume#:#pages# | Articles that do not assess the associations between e-cigarette use and cigarette smoking cessation or initiation. |

**APPENDIX 4: A list of the included studies.**

| **Result #** | **Reference** |
| --- | --- |
| 82 | Lam C, West A. Are electronic nicotine delivery systems an effective smoking cessation tool? Can J Respir Ther. 2015 Fall;51(4):93-8. Review. |
| 103 | Franck C, Budlovsky T, Windle SB, Filion KB, Eisenberg MJ. Electronic cigarettes in North America: history, use, and implications for smoking cessation. Circulation. 2014 May 13;129(19):1945-52. |
| 128 | Gentry S, Forouhi NG, Notley C. Are Electronic Cigarettes an Effective Aid to Smoking Cessation or Reduction Among Vulnerable Groups? A Systematic Review of Quantitative and Qualitative Evidence. Nicotine Tob Res. 2019 Apr 17;21(5):602-616. |
| 140 | Hartmann-Boyce J, McRobbie H, Bullen C, Begh R, Stead LF, Hajek P. Electronic cigarettes for smoking cessation. Cochrane Database Syst Rev. 2016 Sep 14;9:CD010216. |
| 158 | El Dib R, Suzumura EA, Akl EA, Gomaa H, Agarwal A, Chang Y, Prasad M, Ashoorion V, Heels-Ansdell D, Maziak W, Guyatt G. Electronic nicotine delivery systems and/or electronic non-nicotine delivery systems for tobacco smoking cessation or reduction: a systematic review and meta-analysis. BMJ Open. 2017 Feb 23;7(2):e012680. |
| 160 | Soneji S, Barrington-Trimis JL, Wills TA, Leventhal AM, Unger JB, Gibson LA, Yang J, Primack BA, Andrews JA, Miech RA, Spindle TR, Dick DM, Eissenberg T, Hornik RC, Dang R, Sargent JD. Association Between Initial Use of e-Cigarettes and Subsequent Cigarette Smoking Among Adolescents and Young Adults: A Systematic Review and Meta-analysis. JAMA Pediatr. 2017 Aug 1;171(8):788-797. |
| 163 | McRobbie H, Bullen C, Hartmann-Boyce J, Hajek P. Electronic cigarettes for smoking cessation and reduction. Cochrane Database Syst Rev. 2014;(12):CD010216. |
| 166 | Liu X, Lu W, Liao S, Deng Z, Zhang Z, Liu Y, Lu W. Efficiency and adverse events of electronic cigarettes: A systematic review and meta-analysis (PRISMA-compliant article). Medicine (Baltimore). 2018 May;97(19):e0324. |
| 196 | Malas M, van der Tempel J, Schwartz R, Minichiello A, Lightfoot C, Noormohamed A, Andrews J, Zawertailo L, Ferrence R. Electronic Cigarettes for Smoking Cessation: A Systematic Review. Nicotine Tob Res. 2016 Oct;18(10):1926-1936. |
| 199 | Khoudigian S, Devji T, Lytvyn L, Campbell K, Hopkins R, O'Reilly D. The efficacy and short-term effects of electronic cigarettes as a method for smoking cessation: a systematic review and a meta-analysis. Int J Public Health. 2016 Mar;61(2):257-67. |
| 200 | Kalkhoran S, Glantz SA. E-cigarettes and smoking cessation in real-world and clinical settings: a systematic review and meta-analysis. Lancet Respir Med. 2016 Feb;4(2):116-28. |
| 210 | Rahman MA, Hann N, Wilson A, Mnatzaganian G, Worrall-Carter L. E-cigarettes and smoking cessation: evidence from a systematic review and meta-analysis. PLoS One. 2015 Mar 30;10(3):e0122544. |
| 215 | Gualano MR, Passi S, Bert F, La Torre G, Scaioli G, Siliquini R. Electronic cigarettes: assessing the efficacy and the adverse effects through a systematic review of published studies. J Public Health (Oxf). 2015 Sep;37(3):488-97. |
| 222 | Pepper JK, Brewer NT. Electronic nicotine delivery system (electronic cigarette) awareness, use, reactions and beliefs: a systematic review. Tob Control. 2014 Sep;23(5):375-84. |
| 245 | Harrell PT, Simmons VN, Correa JB, Padhya TA, Brandon TH. Electronic nicotine delivery systems ("e-cigarettes"): review of safety and smoking cessation efficacy. Otolaryngol Head Neck Surg. 2014 Sep;151(3):381-93. |
| 246 | Glasser A, Abudayyeh H, Cantrell J, Niaura R. Patterns of E-Cigarette Use Among Youth and Young Adults: Review of the Impact of E-Cigarettes on Cigarette Smoking. Nicotine Tob Res. 2019 Sep 19;21(10):1320-1330. |
| 251 | Patil S, Arakeri G, Patil S, et al. Are electronic nicotine delivery systems (ENDs) helping cigarette smokers quit?-Current evidence. J Oral Pathol Med. 2020;49(3):181-189. |
| 289 | Meernik C, Baker HM, Kowitt SD, et al. Impact of non-menthol flavours in e-cigarettes on perceptions and use: an updated systematic review. BMJ Open. 2019;9:e031598. |
| 293 | Aladeokin A, Haighton C. Is adolescent e-cigarette use associated with smoking in the United Kingdom?: A systematic review with meta-analysis. Tob Prev Cessat. 2019;5:15. |
| 295 | Khouja JN, Suddell SF, Peters SE, Taylor AE, Munafò MR. Is e-cigarette use in non-smoking young adults associated with later smoking? A systematic review and meta-analysis. Tob Control. 2020;tobaccocontrol-2019-055433. |

**APPENDIX 5:** **Characteristics of Systematic Reviews on E-cigarette Use and Combustible Cigarette Smoking Initiation or Cessation.**

| **Characteristic** | **n (%)** |
| --- | --- |
| Systematic review |  |
| With meta-analysis | 10 (50.0%) |
| Without meta-analysis | 10 (50.0%) |
| Year |  |
| 2014 | 4 (20.0%) |
| 2015 | 3 (15.0%) |
| 2016 | 4 (20.0%) |
| 2017 | 2 (10.0%) |
| 2018 | 1 (5.0%) |
| 2019 | 4 (20.0%) |
| 2020 | 2 (10.0%) |
| Country of first author |  |
| United States | 6 (30.0%) |
| Canada | 4 (20.0%) |
| United Kingdom | 5 (25.0%) |
| China | 1 (5.0%) |
| Australia | 1 (5.0%) |
| Italy | 1 (5.0%) |
| Brazil with cross-appointment in Canada | 1 (5.0%) |
| Saudi Arabia | 1 (5.0%) |
| Statistical heterogeneity methods* |  |
| *I*^2^ | 10 (50.0%) |
| χ^2^ | 1 (5.0%) |
| Not assessed/not reported | 10 (50.0%) |
| Publication bias |  |
| GRADE | 3 (15.0%) |
| Funnel plot | 3 (15.0%) |
| Copas selection model | 1 (5.0%) |
| Not assessed/not reported | 13 (65.0%) |
| Funding source reported |  |
| Yes | 11 (55.0%) |
| No funding source | 4 (20.0%) |
| Not reported | 5 (25.0%) |
| Journal impact factor |  |
| <5.0 | 13 (65.0%) |
| 5.1-10.0 | 4 (20.0%) |
| 10.1-15.0 | 1 (5.0%) |
| >15.0 | 2 (10.0%) |
| Cochrane review |  |
| Yes | 2 (10.0%) |
| No | 18 (90.0%) |
| Study designs included* |  |
| RCTs only | 1 (5.0%) |
| RCTs and NRSIs | 15 (75.0%) |
| Preclinical/animal models | 1 (5.0%) |
| No study restriction | 2 (10.0%) |
| Not reported | 4 (20.0%) |
| Languages included |  |
| English only | 10 (50.0%) |
| English and French | 1 (5.0%) |
| English and Chinese | 1 (5.0%) |
| Unrestricted | 7 (35.0%) |
| Not reported | 1 (5.0%) |
| Search methods (in addition to comprehensive electronic database search)* |  |
| Manual search of journal articles, Google Scholar, or regulatory websites | 5 (25.0%) |
| Conference proceedings/abstracts | 3 (15.0%) |
| Experts/corresponding authors | 3 (15.0%) |
| Reference lists of relevant articles | 9 (45.0%) |
| metaRegister of controlled trials database | 2 (10.0%) |
| Continued literature surveillance post-search | 2 (10.0%) |
| Other | 2 (10.0%) |
| None reported | 4 (20.0%) |
| Search strategy provided |  |
| Yes | 6 (30.0%) |
| Partially met criteria | 1 (5.0%) |
| Keywords only | 11 (55.0%) |
| No search terms provided | 2 (10.0%) |
| Review topic |  |
| Intention | 0 |
| Initiation | 3 (15.0%) |
| Dependence | 0 |
| Cessation | 15 (75.0%) |
| Initiation and Cessation | 2 (10.0%) |

*Does not equal 100%, as some reviews contained data in more than one category.

GRADE = Grades of Recommendation, Assessment, Development, and Evaluation; NRSI = non-randomized study of interventions; RCT = randomized controlled trial

Note: percentages were rounded to one decimal place.

**APPENDIX 6: Characteristics of Included Studies.**

| **Author (Publication year)** | **Journal Name** | **Smoking Cessation or Initiation** | **Article Type** | **Funding source and conflicts of interest of the review** | **Written protocol prior to review?** |
| --- | --- | --- | --- | --- | --- |
| **Lam and West (2015)(1)** | Canadian Journal of Respiratory Therapy | Cessation | Systematic Review | No financial disclosures or conflicts of interest to declare. | No |
| **Franck et al. (2014)(2)** | Circulation | Cessation | Systematic Review | Funding: Knowledge Synthesis grant from the Canadian Institutes of Health Research;  Conflicts of Interest: One author received funding from Pfizer Canada Inc. to conduct the Evaluation of Varenicline (Champix) in Smoking Cessation for Patients Post-Acute Coronary Syndrome (EVITA) trial (NCT00794573) of varenicline vs placebo after acute coronary syndrome. The other authors report no conflicts. | No |
| **Gentry et al. (2019)(3)** | Nicotine & Tobacco Research | Cessation | Systematic Review | Funding: not reported;  Conflicts of Interest: none to declare. | Yes |
| **Hartmann-Boyce et al. (2016)(4)** | Cochrane Database of Systematic Reviews | Cessation | Systematic Review and Meta-Analysis | Funding: (internal) Queen Mary University of London, UK and University of Auckland, New Zealand; no external sources of funding supplied;  Conflicts of Interest: Within the last 3 yrs one author received honoraria for speaking at research symposia and received beneﬁts in kind and travel support from, and has provided consultancy to, the manufacturers of smoking cessation medications. Within the last 3 yrs one author provided consultancy for and received research funding from GSK, Pﬁzer, Novartis, and other manufacturers of smoking cessation medications. Two authors have additional declarations: two authors were investigators on a study of e-cigarettes from an e-cigarette manufacturer (Ruyan Group, Beijing and Hong Kong). Ruyan supplied the e-cigarettes used in the trial and contracted with Health New Zealand Ltd. to undertake the study. Health New Zealand Ltd funded The University of Auckland to conduct the trial, independently of Ruyan Group (Holdings) Ltd. The trial design conduct, analysis and interpretation of results were conducted independently of the sponsors. Two authors were investigators on the ASCEND e-cigarette trial funded by the Health Research Council of New Zealand that used product supplied at no charge from PGM international, a retailer of e-cigarettes. The other authors have no conﬂicts of interest to declare. | Yes |
| **El Dib et al. (2017)(5)** | BMJ Open | Cessation | Systematic Review and Meta-Analysis | Funding: WHO grant; the funding agencies played no role in the conduct of the research or preparation of the manuscript;  Conflicts of Interest: author received a Brazilian Research Council scholarship. | No |
| **Soneji et al. (2017)(6)** | JAMA Pediatrics | Initiation | Systematic Review and Meta-Analysis | Funding: National Cancer Institute at the NIH, US FDA Center for Tobacco Products, and the National Institute on Drug Abuse at the NIH; the funders had no role in the design and conduct of the study; collection, management, analysis, and interpretation of the data; preparation, review, or approval of the manuscript; or decision to submit manuscript for publication;  Conflicts of Interest: paid consultant in litigation against the tobacco industry and reported being named on a patent application for a device that measures the puffing behavior of e-cigarette users. | No |
| **McRobbie et al. (2014)(7)** | Cochrane Database of Systematic Reviews | Cessation | Systematic Review and Meta-Analysis | Funding: (internal) Queen Mary University of London, UK, and the University of Auckland, New Zealand; no external sources of support supplied;  Conflicts of Interest: one author undertook educational sessions sponsored by Pfizer and Johnson & Johnson (manufacturers of smoking cessation medications); provided consultancy to GSK, Pfizer, and Johnson & Johnson (manufacturers of smoking cessation medications); investigators on a study of e-cigarettes from an e-cigarette manufacturer (Ruyan Group, Beijing and Hong Kong); investigators on the ASCEND e-cigarette trial funded by the Health Research Council of New Zealand that used product suppled at no charge from PGM international (a retailer of e-cigarettes). | Yes |
| **Liu et al. (2018)(8)** | Medicine | Cessation | Systematic Review and Meta-Analysis | Funding: not reported;  Conflicts of Interest: none to declare. | No |
| **Malas et al. (2016)(9)** | Nicotine & Tobacco Research | Cessation | Systematic Review | Funding: Ministry of Health and Long-Term Care (Ontario, Canada);  Conflicts of Interest: none to declare. | Yes |
| **Khoudigian et al. (2016)(10)** | International Journal of Public Health | Cessation | Systematic Review and Meta-Analysis | Funding: the authors received no financial support for the research, authorship, and/or publication of this article;  Conflicts of Interest: none to declare. | Unclear |
| **Kalkhoran and Glantz (2016)(11)** | Lancet Respiratory Medicine | Cessation | Systematic Review and Meta-Analysis | Funding: NIH, National Cancer Institute, FDA Center for Tobacco Products; the funders had no role in the study design, collection, analysis, or interpretation of the data, or writing of the report;  Conflicts of Interest: one author is a Truth Initiative Foundation Distinguished Professor of Tobacco Control; authors declare no competing interests. | Protocol was registered in PROSPERO after search began |
| **Rahman et al. (2015)(12)** | PLoS ONE | Cessation | Systematic Review and Meta-Analysis | Funding: the authors had no support or funding to report;  Conflicts of Interest: none to declare. | No |
| **Gualano et al. (2015)(13)** | Journal of Public Health | Cessation | Systematic Review | Not reported | No |
| **Pepper and Brewer (2014)(14)** | Tobacco Control | Cessation and Initiation (initiation included but no longitudinal studies investigated initiation; one gateway study included) | Systematic Review | Funding: Cancer Control Education Program at University of North Carolina Lineberger Comprehensive Cancer Center;  Conflicts of Interest: not reported. | No |
| **Glasser et al. (2019)(15)** | Nicotine & Tobacco Research | Cessation and Initiation | Systematic Review | Funding: Truth Initiative (the views in the article do not necessarily represent those of Truth Initiative);  Conflicts of Interest: none to declare. | No |
| **Harrell et al. (2014)(16)** | Otolaryngology‑Head and Neck Surgery | Cessation | Systematic Review | Funding: National Cancer Institute Behavioral Oncology;  Conflicts of Interest: one author receives support from Pfizer, Inc.; the authors alone are responsible for the content and writing of this paper. | No |
| **Patil et al. (2020)(17)** | Journal of Oral Pathology & Medicine | Cessation | Systematic Review | Funding: Not reported  Conflicts of Interest: Not reported | No |
| **Meernik et al. (2019)(18)** | BMJ Open | Cessation | Systematic Review | Funding: No sources of funding.  Conflicts of Interest: No conflicts of interest. | No |
| **Aladeokin and Haighton (2019)(19)** | Tobacco Prevention & Cessation | Intention and Initiation | Systematic Review and Meta-Analysis | Funding: No sources of funding.  Conflicts of Interest: No conflicts of interest. | Yes |
| **Khouja et al. (2020)(20)** | Tobacco Control (BMJ Group) | Initiation | Systematic Review and Meta-Analysis | Funding: UK Centre for Tobacco and Alcohol Studies; British Heart Foundation; Cancer Research UK; Economic and Social Research Council; Medical Research Council; NIHR; the Medical Research Centre Integrative Epidemiology Unit at the University of Bristol; and the NIHR Biomedical Research Centre at University Hospitals Bristol NHS Foundation Trust and the University of Bristol.  Conflicts of Interest: Two authors receive grants from Pfizer, Inc., outside of this publication. | Yes |

BMJ = British Medical Journal; e-cigarette = electronic cigarette; FDA = Food and Drug Administration; NIH = National Institutes of Health; NIHR = National Institute for Health Research; NHS = National Health Service; PROSPERO = Prospective Register of Systematic Reviews; UK = United Kingdom; US = United States; vs = versus; WHO = World Health Organization; yr = year

**APPENDIX 7: Methodology and Included Studies in the Included Systematic Reviews.**

| **Author (Publication year)** | **Search strategy methods and databases searched** | **Review inclusion and exclusion criteria** | **Study selection and data extraction methodology of the review** | **# of articles included in the review** | **# of articles included in meta-analysis** | **List of excluded articles from the review and justification** | **Description of studies included in the review** |
| --- | --- | --- | --- | --- | --- | --- | --- |
| **Lam and West (2015)(1)** | Databases searched: PubMed, Scopus, and Web of Science Core Collection;  A primary search was conducted in February 2015. Keywords for the search included “electronic cigarette,” “e-cigarette,” “cigarette vapor,” “electronic nicotine delivery system,” “smoking cessation,” “electronic cigarette smoking cessation,” “quit smoking,” and “tobacco cessation.” A secondary search was then conducted by reviewing references of topic-related articles. All searches excluded non-English publications and were limited to RCTs. No limitations with respect to the date of publication were applied. | Studies were included in the review only if they were RCTs in which the effectiveness of ENDS as a smoking cessation tool was investigated. All methodologies other than RCTs were excluded. With respect to the outcome measures of these RCTs, more specific inclusion/exclusion criteria were not applied to maximize sensitivity of the search to identify relevant articles. This was particularly important given the limited number of publications expected in this emerging research area. | A single investigator conducted the primary search and abstract review. Data pertaining to study design, objective, population and intervention were extracted by one reviewer and verified by a second reviewer. Articles were selected through abstract review based on confirmation of the research design and other inclusion criteria. Both investigators then independently conducted full-text reviews to exclude relevant publications that did not meet inclusion criteria. Any disagreements between the investigators were resolved by consensus. No attempt was made to contact authors for missing information. The characteristics of each included publication (design, objective, sample, intervention and primary findings) were then tabulated for qualitative analysis. | 4 articles | N/A | No | Study setting and duration: setting not specified in the review; follow-up ranged from 12 weeks to 52 weeks with one study measuring short-term effects of an electronic nicotine delivery device after 60 min;  Population and participant characteristics: adult patients 18‑70 yrs with smoking history of a minimum of 10 factory-made cigarettes per day for at least 1 yr with or without the motivation to quit;  Interventions: ENDS;  Comparators: conventional cigarette smokers without treatment, nicotine inhaler, nicotine patches, non-nicotine ENDS;  Outcomes: smoking cessation;  Study design: RCTs. |
| **Franck et al. (2014)(2)** | Databases searched: PubMed;  The authors conducted a systematic search to identify experimental trials examining the efficacy or safety of e-cigarettes for smoking cessation published before September 15, 2013. The search was conducted according to the PRISMA guidelines for systematic reviews. Key search terms included “electronic cigarette” and “e-cigarette.” The authors searched PubMed, Google Scholar, and regulatory websites (e.g., Health Canada, US FDA) manually for additional articles providing context for the awareness and use of e-cigarettes, their regulation, and potential ethical issues. | Included publications from the systematic search contained experimental studies examining the efficacy or safety of an e-cigarette for smoking cessation. Exclusion criteria included articles without original data, articles limited to pharmacokinetic analyses, abstracts, conference proceedings, and articles not published in English or French. | Data were extracted by two reviewers, with disagreements resolved by consensus or by a third reviewer. Extracted data included country of origin, study methods, study population, treatment type and duration, duration of follow-up, study results, and key takeaways. | 7 articles | N/A | No | Study setting and duration: New Zealand, Italy, UK; treatment durations ranged from 1 day to 52 weeks;  Population and participant characteristics: current smokers with or without motivation to quit (or with no information on motivation), including one study in schizophrenic inpatients; sample sizes ranging from 14 to 657 patients;  Interventions: e-cigarettes;  Comparators: placebo e-cigarette, just-hold e-cigarette, nicotine patch, nicotine inhaler, cigarette;  Outcomes: smoking cessation;  Study design: RCTs; mixed experimental design; randomized, repeated-measures crossover; non-randomized, interventional design. |
| **Gentry et al. (2019)(3)** | Databases searched: MEDLINE;  Following searches from similar systematic reviews, a strategy was developed using MeSH and free text terms, tested against a sample of relevant papers and adapted for other databases. Searches were from 2004, when modern e-cigarettes became available, to March 2017. Reference lists of included studies and systematic reviews were screened. Searches were not restricted by language, but studies without a full text available in English would have been excluded, although none relevant were identified. Articles not referring to any included vulnerable group(s) or to e-cigarettes by any recognizable name, in the title/abstract, were excluded. | Inclusion criteria:  Study design: A range of designs were included as scoping searches suggested limited available controlled evidence. The following study designs were eligible:   - For assessing effectiveness: RCTs, cluster RCTs, quasi-RCTs, controlled before and after studies, interrupted time series, cohort studies, case–control studies, and uncontrolled before and after studies. - For assessing quantitative data on barriers and facilitators to e-cigarette use: longitudinal, cross-sectional, or cohort surveys. - For assessing qualitative data on barriers and facilitators to e-cigarette use: qualitative studies with any recognized method of data collection (e.g., interviews, focus groups) and analysis from any discipline or theoretical tradition (e.g., grounded theory, thematic analysis).   Participants: Participants and carers of any age in any country/setting in at least one of the following vulnerable groups: mental illness, substance misuse, homeless, or criminal justice system.  Interventions: Studies investigating e-cigarettes, defined as “electronic devices that heat a liquid into an aerosol for inhalation. “Heat not burn” products, in which heated tobacco is vaporized, were excluded.  Comparison group: another type of nicotine or non-nicotine e-cigarette, smoking cessation intervention (e.g., NRT or behavioral intervention), or no or delayed intervention.  Outcome measures: studies reporting on any of the primary or secondary outcomes were included. Primary outcomes: smoking cessation at longest follow-up, serious or non-serious adverse events, and perceived barriers and facilitators to e-cigarette use. Secondary outcomes: smoking reduction, retention in a smoking cessation, substance misuse, mental health, or other treatment program, and health economics outcomes. | Search results were merged using Endnote and de-duplicated. Titles and abstracts were screened according to pre-specified inclusion/exclusion criteria by one author with 10% double screened by a second author. There were two discrepancies, which were resolved by discussion. Potentially included full text articles were retrieved and reviewed and 10% double screened, with no discrepancies. Data were extracted using a standardized data extraction sheet and a sample (four studies) double checked by a second reviewer, with no discrepancies. Double screening and data extraction of only a sample was necessary due to resource limitations, and has been done in similar reviews. | 9 studies (9 articles and 1 conference abstract) | N/A | No | Study setting and duration: US, Australia, Italy, New Zealand, and international posters online; follow-up ranged from 4 weeks to 18 months for quantitative studies;  Population and participant characteristics: cigarette smokers with mental illness, homeless participants that are cigarette smokers, or substance misusers that are cigarette smokers. Mental illness populations were heterogeneous and included people reporting being prescribed one or more psychiatric medications, serious mental illness diagnosis, schizophrenia, and acute psychiatric admissions. Qualitative studies included community mental health clients and posters discussing e-cigarettes in the context of mental illness online. Two qualitative and no quantitative studies involving homeless populations were identified. One study recruited from homeless shelters. The second study included homeless parents living in family shelters. Only one quantitative study and no qualitative studies focused on substance misusers. The study involved people on methadone and may not be representative of users of other substances;  Interventions: free provision of e-cigarettes (with or without behavioral support). Only one study included behavioral support (low intensity voluntary telephone counseling). One study offered e-cigarette use instructions plus telephone technical and medical assistance. The remaining studies provided only instructions for use. No explicit theoretical basis for interventions were described. One study emphasized collecting “real-life” data hence no encouragement or motivational support was provided. All suggested e-cigarettes may be considered a harm reduction strategy;  Comparators: placebo nicotine cartridges, nicotine patch, non-e-cigarette users;  Outcomes: smoking cessation, adverse events, smoking reduction, and barriers and facilitators to e-cigarette use;  Study design: five quantitative studies were included (total participants n = 1089). Of the included quantitative interventional studies (total participants n = 133), there was one secondary analysis of an RCT, and three uncontrolled before and after studies (one study was reported in both a conference abstract and a full article). One cohort observational study was included (n = 956). Four qualitative studies were included; three involving focus groups (n = 128) and one qualitative analysis of online postings. |
| **Hartmann-Boyce et al. (2016)(4)** | Databases searched: Cochrane Tobacco Addiction Group Specialized Register, Cochrane Central Register of Controlled Trials (CENTRAL) (the Cochrane Library, 2016, Issue 1), MEDLINE (OVID SP) (2004 to 2016 January week 2, & MEDLINE in process/In data review February 1, 2016), Embase (OVID SP) (2004 to 2016 week 5), and PsycINFO (OVID SP) (2004 to 2016 January week 4);  The authors performed the search in January 2016. The search terms were broad and included e-cig$ OR elect$ cigar$ OR electronic nicotine as well as the terms vape or vaper or vapers or vaping. The search date parameters are limited to 2004 to the present, due to the fact that e-cigarettes were not available before 2004.  The authors searched the reference lists of studies found in the literature search and the metaRegister of controlled trials database (www.isrctn.com/page/mrct). The authors also contacted authors of known trials and other published e-cigarette studies. | RCTs in which smokers are randomized to e-cigarettes or to a control condition, and which measure abstinence rates at 6 months or longer, to determine the efﬁcacy of e-cigarettes in aiding smoking cessation and reduction. Additionally, the results from cohort follow-up studies with 6 months’ or longer follow-up were considered. | Study selection and data extraction were performed in duplicate. Data were extracted on author; date and place of publication; study design; inclusion and exclusion criteria; setting; summary of study participant characteristics; summary of intervention and control conditions; number of participants in each arm; smoking cessation outcomes; type of biochemical validation (if any); adverse events, serious adverse events, and relevant biomarkers; assessment time points; Risk of Bias; and additional comments. Data were entered into Review Manager 5 and verified by a second author. | 2 studies included in meta-analysis; 22 observational studies narratively synthesized; 27 ongoing studies | 2 studies | Yes | Study setting and duration: New Zealand, US, Italy, international survey, Switzerland, and UK; follow-up ranged from 6 months to 24 months (for cessation data);  Population and participant characteristics: smokers with or without motivation to quit (including 1 study of patients with schizophrenia);  Interventions: e-cigarettes (with or without bupropion and varenicline);  Comparators: nicotine patches, non-nicotine e-cigarettes, no treatment/no e-cigarette use;  Outcomes: smoking abstinence at 6 months or longer and/or adverse events;  Study design: RCTs, cohort studies, intervention studies, and non-intervention studies. |
| **El Dib et al. (2017)(5)** | Authors used MeSH terms based on the terms “electronic nicotine,” “smoking-cessation,” “tobacco-use-disorder,” “tobacco-smoking,” and “quit.” Medline, EMBASE, PsycInfo, CINAHL, Cochrane Central Register of Controlled Trials, ISI Web of Science, and the trial registry (clinicaltrials.gov) were searched. The authors also established a literature surveillance strategy based on the weekly search alerts by Centers for Disease Control and Prevention's Smoking and Health Resource Library of published articles as well as the Gene Borio's daily news items. The surveillance strategy started from the time of running the comprehensive literature search up to the time of the submission of the manuscript. | Eligibility criteria:  Study designs: RCTs and prospective cohort studies;  Participants: cigarette smokers, regardless of whether the users were using them as part of a quit attempt;  Interventions: ENDS or ENNDS;  Comparators: no smoking cessation aid, alternative non-electronic smoking cessation aid, alternative electronic smoking cessation aid;  Outcomes: tobacco smoking cessation (with preference to biochemically validated outcomes) measured at 6 months or longer follow-up, reduction of cigarette use of at least 50%, serious and non-serious adverse events measured at 1 week or longer follow-up. | Three pairs of reviewers underwent calibration exercises and used standardized pilot-tested screening forms. They worked in teams of two and independently screened all titles and abstracts identiﬁed by the literature search, obtained full-text articles of all potentially eligible studies and evaluated them for eligibility. Reviewers also worked in pairs to independently extract data from included studies, using a pretested data extraction form for the following data: study design, participants, interventions, comparators, outcome assessed, and relevant statistical data. Reviewers resolved disagreement by discussion or, if necessary, with third party adjudication. Study authors were contacted for additional information. | 12 studies (19 publications), including 3 RCTs (8 publications) and 9 cohort studies (11 publications) | 8 studies included in meta-analysis for smoking cessation | No | Study setting and duration: New Zealand, US, UK, and Europe; studies followed patients from 4 weeks to 36 months;  Population and participant characteristics: cigarette smokers with or without motivation to quit. Randomized trials sample size ranged from 50 to 657, and observational studies from 100 to 3891. Typical participants were women in their 40s and 50s;  Interventions: ENDS;  Comparators: Of the three RCTs, one compared ENDS with NRT and ENNDS, another compared different concentrations of ENDS with ENNDS and the third compared different types of ENDS. Only one study included participants who were also currently receiving other behavioral and pharmacologic treatment. The participants from one study were all enrolled in a state quitline program that provided behavioral treatment and in some cases NRT. All nine cohort studies compared ENDS with no use of ENDS or tobacco cigarettes only; in one, exposure and non-exposure groups received behavioral and other pharmacologic treatment;  Outcomes: smoking cessation and tobacco quit rates;  Study design: parallel RCTs and cohort studies. |
| **Soneji et al. (2017)(6)** | Databases searched: MEDLINE’s PubMed (1946 to present), EMBASE (1974 to present), Wiley’s Cochrane Library (2016 issue 7), and Web of Science (1900 to present) between February 7 and February 17, 2017; comprehensive search of the 2016 Society for Research on Nicotine and Tobacco 22^nd^ Annual Meeting abstracts, the 2016 Society of Behavioral Medicine 37^th^ Annual Meeting & Scientific Sessions abstracts, and the 2016 NIH Tobacco Regulatory Science Program Conference.  The search included indexed terms and text words to capture concepts associated with e-cigarettes and traditional cigarettes. There were no language or study design restrictions. The search strategy was adjusted for the syntax appropriate for each database. | Inclusion criteria: studies evaluating the association between e-cigarette use among never cigarette smokers at baseline and cigarette smoking initiation between baseline and follow-up; studies that evaluated the association between past 30-day e-cigarette use at baseline and past 30-day cigarette smoking at follow-up; longitudinal studies;  Exclusion criteria: news summaries, cross-sectional study designs, letters, review articles, editorials, commentaries, qualitatives, studied cigarette smoking cessation, prospective cohort studies but did not assess cigarette smoking initiation by e-cigarette status, prospective cohort study but baseline respondents already cigarette smokers, and prospective cohort study but temporal order of e-cigarette use and cigarette smoking initiation could not be established. | Three investigators reviewed the titles, abstracts, and texts (interrater agreement by Fleiss kappa, 86.1%). When the investigators disagreed, they discussed to reach consensus.  Data extracted included the following: study location, comparison group (e.g., never e-cigarette users), time between baseline and follow-up, and a list of demographic, psychosocial, and behavioral characteristics included in each study’s multivariable statistical analysis. Demographic characteristics included age, sex, race/ethnicity, and parental educational level. Psychosocial and behavioral characteristics included levels of self-esteem, sensation seeking, rebelliousness, delinquent behavior, depressive symptoms, impulsivity, smoking susceptibility, peer smoking, parental smoking, and use of other substances (alcohol, illicit drugs, and other tobacco products). | 9 studies | 9 studies | No | Study setting and duration: US (including 5 studies that were regional: Los Angeles, California; Oahu, Hawaii; Richmond, Virginia), US national-based samples recruited through random-digit dial, nationally representative online panels, and nationally representative school-based samples; follow-up ranged from 6 to 18 months;  Population and participant characteristics: never smokers at baseline. The 9 included studies comprised 16621 adolescents and young adults (14‑30 yrs at baseline); 56% were female;  Interventions: ever e-cigarette use; past 30-day e-cigarette use;  Comparators: never e-cigarette users, non-past 30-day e-cigarette users;  Outcomes: cigarette smoking initiation between baseline and follow-up and past 30-day cigarette smoking;  Study design: longitudinal surveys. |
| **McRobbie et al. (2014)(7)** | Databases searched: Cochrane Tobacco Addiction Group Specialised Register, Cochrane Central Register of Controlled Trials (*The Cochrane Library*, Issue 7, 2014), MEDLINE (OVID SP) (2004 to July 2014), EMBASE (OVID SP) (2004 to July 2014), PsycINFO (OVID SP) (2004 to July 2014), and CINAHL (EBSCO Host) (2004 to July 2014);  Databases were searched in July 2014. The search terms were broad and included e-cig$ OR elect$ cigar$ OR electronic nicotine. | Inclusion/exclusion criteria:  Types of studies: RCTs in which smokers were randomized to e-cigarettes or a control condition, and which measure abstinence rates or changes in cigarette consumption at 6 months or longer; randomized cross-over trials and cohort follow-up studies with follow-up greater than 1 week for assessment of adverse events; cross-sectional studies were excluded; included studies regardless of publication status or language;  Participants: current smokers at enrolment, either motivated or unmotivated to quit;  Interventions: e-cigarettes;  Comparators: placebo e-cigarettes, alternative smoking cessation aids (including NRT or no intervention), and standard smoking cessation treatment (behavioral, pharmacological, or both);  Outcomes: cessation at least 6 months from the start of the intervention, reduction in cigarette use at least 6 months from the start of the intervention, adverse events at 1 week or more after the start of the intervention. | Two review authors independently pre-screened all titles and abstracts using a screening checklist. When there was a disagreement, the full-text version was obtained, and the disagreement was resolved by discussion with a third review author. Full-text versions of the potentially relevant papers were obtained and independently screened for inclusion by two reviewers. Any disagreement was resolved with a third reviewer.  Two review authors extracted data from the included studies and checked them against each other. A third reviewer was available to review and resolve any discrepancies. Data were extracted for the following: author, date and place of publication, study design, inclusion and exclusion criteria, setting, summary of study participant characteristics, summary of intervention and control conditions, number of participants in each arm, smoking cessation outcomes, cigarette use per day, type of biochemical validation (if any), adverse events and serious adverse events, assessment time points, RoB, and additional comments. Data were entered into Review Manager 5 software for analyses. | 29 records (representing 13 completed and 9 ongoing studies) | 2 studies | Yes | Study setting and duration: New Zealand, Italy, international survey, Switzerland, and UK; follow-up ranged from 6 months to 24 months (for cessation data);  Population and participant characteristics: smokers with or without motivation to quit (one study included patients with schizophrenia);  Interventions: e-cigarettes (with or without bupropion and varenicline)  Comparators: nicotine patches, non-nicotine e-cigarettes;  Outcomes: changes in smoking behavior over time and adverse events;  Study design: RCTs and prospective cohort studies. |
| **Liu et al. (2018)(8)** | Databases searched: PubMed, EMBASE, web of science, Google scholar, the Chinese Medical Citation Index, and the CENTRAL database of the Cochrane Library;  The search terms included: “electronic cigarette(s),” “e-cigarette(s),” “e-cig(s),” “smoking alternatives,” “electronic cigarettes vapor,” “e-cigarettes liquid,” “e-cig composition,” “e-cig chemicals,” “e-cig chemical composition,” “electronic cigarettes gas,” “electronic cigars,” “electronic nicotine delivery device,” and “electronic nicotine delivery systems.” The language of the papers was restricted to English and Chinese. The search covered the literature published from January 2003 to July 2017. | Inclusion criteria: (met all of the following criteria) smoking reduction and smoking cessation attributed to e-cigarettes; smokers had a consumption history of both e-cigarettes and cigarettes; the chemical constituent of the e-cigarettes was reported; and a quantitative assessment of the efficiency of e-cigarettes was presented by calculating the accurate values of smoking reduction, smoking cessation, and quit failures;  Exclusion criteria: (met any of the following criteria) participants who had diseases reported in studies; description of the baseline conditions of participants and e-cigarettes was unclear; endpoint indicators of smoking reduction, smoking cessation, and adverse events was absent; and any duplicated publications, reviews, and systematic reviews. | Data were independently extracted by two reviewers. Data extracted included: description of the study population (age, gender, sample size, and groups), study details (first author, year of publication, country of publication, study designs, and endpoint indicators), and pooled data (rates of smoking reduction, smoking cessation, adverse events, and measured CO_2_ exhalation levels). Any disagreements were resolved after consulting a third researcher and reaching a consensus. | 14 studies | 14 studies | No | Study setting and duration: not specified in the review;  Population and participant characteristics: cigarette smokers;  Interventions: e-cigarette use;  Comparators: not specified in the review;  Outcomes: smoking reduction, smoking cessation, and adverse events;  Study design: RCTs, observational studies, and surveys. |
| **Malas et al. (2016)(9)** | Databases searched: PubMed, MEDLINE, PsycINFO, CINAHL, ERIC, ROVER, Scopus, ISI Web of Science, Cochrane Library, and the Ontario Tobacco Research Unit library catalogue. Grey literature was searched using Grey Matters, OAIster, Open Grey, the NYAM website, the Legacy Library, BIOSIS Previews, Conference Papers Index, ISI Proceedings, Dissertation Abstracts International, CIHI, and Grey Net International.  Where possible, searches queried both title and keyword for official and slang terms related to e-cigarettes and smoking cessation. Queries searched publications up to February 1, 2016. Authors of abstracts, posters, and presentations were contacted for further information; those who did not respond or showed potential conflict of interest were excluded. | Any English-language publications that contained original data related to e-cigarettes and smoking cessation (except studies on public awareness only), excluding literature reviews, business reports, commentaries and discussion papers, news articles, fact sheets, and position/policy statements. | References were screened by two reviewers using DistillerSR. Inclusion required one reviewer and exclusion required two reviewers (blind to each other). Conflicts were automatically flagged and resolved through discussion or by a third reviewer. | 62 articles | N/A | No | Study setting and duration: study setting was not specified in the review for all studies but study settings included: Kansas (US), International Tobacco Control Four-Country Survey (US, UK, Canada, and Australia), a representative sample from two metropolitan US regions, and England; duration not reported in all studies, however, follow-up was 6 months or longer where reported;  Population and participant characteristics: smokers from the general population;  Interventions: e-cigarette use;  Comparators: NRTs, no control, no smoking cessation aid, placebo, nonusers;  Outcomes: smoking abstinence or reduction, withdrawal symptoms, cravings or urges to smoke;  Study design: RCTs, experimental studies, longitudinal studies, and cross-sectional studies. |
| **Khoudigian et al. (2016)(10)** | Databases searched: (using the OVID interface, up to May 26, 2014) MEDLINE (1946-present), EMBASE (1974-present), PsychINFO (1860-present), and the Cochrane Central Registry of Controlled Trials (CENTRAL; April 2014). Gray literature was identiﬁed through searching the websites of health technology assessment and related agencies, as well as reports of major smoking cessation conference proceedings. The Google search engine was used to search for additional Web-based materials and information. These searches were supplemented by reviewing the bibliographies of key papers.  Terminology was used to search controlled vocabularies (MeSH and EMTREE) and keywords on the concept of "electronic cigarette" or "e-nicotine." No limits on year, language, or human subjects were applied. | Inclusion criteria: RCTs or comparative observational studies; comparing interventions with nicotine-containing e-cigarettes (any brand, any dose) to other NRTs (e.g., nicotine patches, nicotine gums, nicotine inhalers etc.) or placebo-containing e-cigarettes; healthy adults (≥18 yrs old); current smokers (≥10 cigarettes per day) regardless of whether they were considering quitting; and reports of any of the following outcomes: smoking abstinence for at least 6 months from the start of e-cigarette use; desire to smoke for at least 1 h after e-cigarette use; number of cigarettes smoked per day; withdrawal symptoms (i.e., irritability, restlessness, poor concentration, anxiety, depression, and hunger); serious and non-serious adverse events.  Exclusion criteria: trials with non-human subjects; subjects with comorbidities or other health complications; no comparison group; non-intervention trials (e.g., review, conference abstract, case report, comment, editorial, news, survey, recommendation, or expert opinion). | The reviewers independently screened study titles and abstracts based on the pre-specified inclusion and exclusion criteria. The full text articles of potentially eligible studies were retrieved and assessed by both reviewers independently to confirm inclusion or exclusion. Disagreements were resolved through discussion and consensus.  The reviewers independently extracted data from included studies using predesigned and piloted forms, including details on the following: patient demographics, intervention, comparator, study outcomes, country, year, and length of follow-up. Furthermore, information on the methodology of the study and the funding source(s) was also extracted for quality assessment. The authors were contacted when data were reported in graphical form, were unclear, or missing. A second reviewer veriﬁed the data abstraction. | 5 studies | 5 studies | No | Study setting and duration: New Zealand, UK, and Italy; follow-up ranged from 1 day to 9 months after quit date;  Population and participant characteristics: adult smokers (18 yrs or older; smoked at least 10 cigarettes per day) with or without motivation to quit (or did not specify motivation);  Interventions: e-cigarettes;  Comparators: placebo e-cigarettes, nicotine patches, nicotine inhaler, just hold e-cigarettes, and cigarettes;  Outcomes: smoking abstinence, cigarettes smoked/day, adverse events, cigarette smoking reduction, desire to smoke, withdrawal symptoms, and adverse events;  Study design: RCTs and controlled before-and-after studies. |
| **Kalkhoran and Glantz (2016)(11)** | Databases searched: PubMed and the Web of Science Core Collection.  The search was performed between April 27, 2015 and June 17, 2015. Search terms included “electronic cigarette,” “e-cigarette,” “electronic nicotine delivery,” “stop,” “quit,” “cessation,” “abstain,” and “abstinence.” Search results were not limited by language. There was no search limitation on publication dates. The authors continued to monitor the scientific literature after completing the formal search. Both abstracts and full manuscripts were considered. | Clinical trials, whether randomized and controlled or not, cohort studies, and cross-sectional studies were all considered. Studies that evaluated the relationship between e-cigarette use and cigarette smoking cessation among adult cigarette smokers were included. All study populations that were defined as “adult” by the study authors were considered (youngest age varied from 15 to 30 yrs in the studies that defined “adult”). Studies of participants who were interested in quitting cigarette smoking and studies of all smokers irrespective of interest in quitting were included.  Studies that included cigarette smoking cessation as a primary outcome were evaluated for inclusion. The definitions of cigarette smoking cessation included both self-reported abstinence from smoking cigarettes and biochemically-validated measures of abstinence (e.g., cotinine or exhaled carbon monoxide measurements). All studies were included irrespective of the duration of abstinence from cigarettes. Those who quit cigarettes could have still been using e-cigarettes; quitting e-cigarettes was not used as an outcome. | One investigator did the search, data extraction, and RoB assessment, which was subsequently reviewed by a second investigator. Data extracted from each study included study location, design, population, definition and prevalence of e-cigarette use, comparison group (if applicable), cigarette consumption, level of nicotine dependence, other confounders measured, definition of quitting smoking, and odds of quitting smoking. Study investigators were contacted for missing information. | 38 studies | 20 studies | No | Study setting and duration: US, UK, Italy, Canada, Australia, and New Zealand; duration not specified in the review for all studies, but ranged from 3 months to approximately 2‑3 yrs follow-up where reported;  Population and participant characteristics: adult smokers (including smokers with cancer in a tobacco treatment program, smokers with serious mental illness, hospitalized cigarette smokers, smokers enrolled in a smoking cessation program, college students who had not tried e-cigarettes at baseline, current or former smokers, adult tobacco quitline callers, current or past daily tobacco users with head and neck cancer, smokers interested in quitting, and smokers accessing Stop Smoking Services);  Interventions: e-cigarettes;  Comparators: no e-cigarette use in the past 30 days at baseline, no e-cigarette use in the past 3 months, never tried e-cigarettes or tried no more than two times, NRT, no smoking cessation aid, non-e-cigarette users, those reporting “will never use e-cigarettes” at baseline and follow-up, never tried e-cigarettes or currently do not use e-cigarettes, did not use e-cigarettes as part of their quit program, nicotine patches, non-nicotine e-cigarettes;  Outcomes: smoking cessation  Study design: longitudinal cohort studies, cross-sectional studies, and clinical trials. |
| **Rahman et al. (2015)(12)** | Databases searched: PubMed, Web of Knowledge, and Scopus;  The authors searched the databases using the following terms: “electronic cigarettes OR e-cigarettes” AND “smoking cessation OR quit smoking.” Further search criteria were studies published in English and conducted on humans. There was no limit on publication date. The databases were last searched in May, 2014. | The authors reviewed published studies, including RCTs, cohort, case-control and cross-sectional studies, if they assessed the efficacy of e-cigarettes in achieving smoking abstinence or reduction in cigarette consumption, among current smokers who had used the devices for 6 months or more. Studies with additional outcomes pertaining to smoking reduction were permitted, while those with primary endpoints concerning other issues, such as attenuation of withdrawal symptoms were excluded. | Two researchers conducted a comprehensive literature search according to the method recommended by the Cochrane Collaboration. | 6 studies | 6 studies | No | Study setting and duration: study setting not specified in the review; follow-up ranged from 6 months to 24 months;  Population and participant characteristics: The combined sample size from the selected studies was 7,551. Inclusion and exclusion criteria were also largely common to all selected studies. The predominant inclusion criteria were adult current smokers (although exact definition of smoking status varied), while the main exclusion criteria were comorbid cardiovascular disease, diabetes, major depression and other psychiatric disorders. In two of the studies participants were not intending to quit smoking prior to use of an e-cigarette, while remaining studies’ participants were either intending to quit or had mixed intentions. There was slight variation between the studies in the way smoking status was defined. Most studies defined current smokers as those who had smoked at least 10 cigarettes per day for a period of years. Two studies did not explicitly define current smoking status, however, participants self-reported themselves as either daily or occasional smokers. Finally, one study defined it as participants having smoked ≥100 cigarettes in their lifetime. Recruitment criteria regarding participants’ intention to quit varied across studies; two studies recruited those who were intending to quit, two not intending to quit, and the remaining two studies’ participants had mixed intentions;  Interventions: The intervention implemented in three of the six studies was *ad libitum* use of e-cigarettes, which were provided to participants for the duration of the study period. The remaining studies were cross-sectional and cohort in design, and did not include an intervention, but similarly investigated participants’ *ad libitum* use of e-cigarettes over the study period;  Comparators: Two of the selected studies (both RCTs) included formal comparator groups or interventions. One used groups assigned to nicotine patches and placebo e-cigarettes as comparators, while the other just used a group assigned to placebo e-cigarettes as a comparator. One of the cross-sectional studies compared smoking cessation rates among e-cigarette users to those using NRT and those quitting unaided;  Outcomes: The primary outcome for five of the studies was either abstinence from smoking at the end of the designated study period or reduction in smoking (as measured by number of cigarettes per day). The remaining study primarily investigated longitudinal usage patterns of e-cigarettes but measured smoking cessation and reduction as a secondary outcome. Adverse events and withdrawal symptoms were specifically assessed in both RCTs and in one cohort study;  Study design: RCTs, cross-sectional studies, and prospective cohort studies. |
| **Gualano et al. (2015)(13)** | Databases searched: PubMed, ISI Web of Knowledge, Scopus and Cochrane Controlled Trials Register;  Databases were searched using the following keywords: “electronic nicotine delivery system,” “nicotine device,” “electronic cigarettes,” “e-cigarettes,” and “electronic cigarette smoking” until April 2014. All articles written in English were considered. | Both experimental and observational studies were selected. In particular, the authors selected studies that examined the efﬁcacy of e-cigarettes (in terms of reduction of desire to smoke and/or number of cigarettes smoked and/or quitting or in terms of reduction of nicotine withdrawal symptoms) and the safety of e-cigarettes (adverse events self-reported or clinical/ laboratory measured after using e-cigarettes). | In the first stage, researchers analyzed the search results to find potentially eligible studies. The publications were sorted by title and abstracts, and only eligible studies were selected for full-text review.  The researchers reviewed each eligible full text and extracted the required data. For each study, information about characteristics of the survey, study design, sample size, funding, efﬁcacy (in terms of reduction of desire to smoke and/or number of cigarettes smoked) and/or adverse events (self-reported or clinical/laboratory measured) were retrieved. | 12 studies | N/A | No | Study setting and duration: New Zealand, UK, Greece, US, Italy; length of study ranged from daily (*ad libitum* for 5 min) to 52 weeks;  Population and participant characteristics: cigarette smokers;  Interventions: e-cigarettes;  Comparators: placebo e-cigarettes, nicotine inhaler, tobacco cigarette, just-hold e-cigarette, sham smoking/unlit cigarette, nicotine patches;  Outcomes: efficacy (reduction of desire to smoke and/or number of cigarettes smoked, reduction of nicotine withdrawal symptoms), short-term adverse effects, and physiological effects;  Study design: experimental studies and prospective cohort studies. |
| **Pepper and Brewer (2014)(14)** | Databases searched: PubMed, CINAHL, Web of Science, EMBASE, and PsychINFO;  Databases were searched from January 1, 2006 to July 1, 2013. Search terms used were: “electronic cigarette,” OR “electronic cigarettes,” OR “e-cigarette,” OR “e-cigarettes,” OR “electronic nicotine delivery.” The authors selected this set of broad search terms as no relevant MeSH terms existed at the time of this review. The authors also searched the reference sections of included articles. | Conference or dissertation abstracts and articles not in English were excluded. Articles that were not relevant to ENDS were also excluded. The authors excluded articles that did not contain original data about ENDS, such as commentaries, literature reviews, and information about regulation; experiments or laboratory studies without descriptions of ‘natural’ patterns of use (i.e., usage not instructed by the researcher) or subjective reports from participants on relevant dependent variables; not peer-reviewed, such as industry reports; or did not include appropriate dependent variables (i.e., they reported data about internet search engines or pharmacies). | Two authors reviewed the titles and abstracts, and conference or dissertation abstracts and articles not in English were discarded. In a second step, one author reviewed the remaining abstracts, and when necessary, full articles, and conferred with the second author where eligibility was unclear.  The first author coded the remaining articles using a standardized data abstraction form. The second author or one of two additional coders reviewed each article, conferring with the first author in case of disagreements. Coders recorded ENDS awareness, natural patterns of use (i.e., use outside of a lab setting, including dual use of ENDS with other tobacco products), subjective reactions to use (by users only), and beliefs or reasons for use (by users or non-users). The authors defined ‘dual use’ as use of both ENDS and other tobacco products in the past 30 days. For the last two outcomes, coders also recorded whether the measure assessed: the perceived cost of ENDS, including the relative cost of ENDS and regular cigarettes (cost); the possibility that ENDS would serve as a gateway to other tobacco use (gateway); health, safety and side effects, including the relative safety of ENDS and regular cigarettes (health); quitting or reducing smoking or tobacco use because of ENDS; the use of ENDS to avoid restrictions on smoking; the degree of satisfaction with ENDS’ taste, smell and quality; the extent to which ENDS have the same taste, smell or feeling of use as regular cigarettes; and changes in withdrawal symptoms, desire to smoke, and cravings. Financial relationships with the ENDS industry were also noted. | 49 studies from 47 articles | N/A | No | Study setting and duration: most studies were conducted in the US (23 studies) or with participants from multiple countries (7 studies). Other common locations included Italy (5 studies) and the UK (4 studies); follow-up duration not reported in the review for most studies;  Population and participant characteristics: current and former smokers, current and former ENDS users, included a case study of one patient with lipid pneumonia;  Interventions: e-cigarettes;  Comparators: cigarettes, Nicorette inhalators, non-nicotine ENDS, non-tobacco users, just holding ENDS, just holding cigarettes, ENDS naïve smokers;  Outcomes: awareness of ENDS, use of ENDS (ever use, current use, dual use, amount and duration of use), subjective reactions to using ENDS (cost, gateway use, health and safety, quitting or reducing tobacco use, restrictions on smoking, satisfaction, similarity to regular cigarette smoking, withdrawal symptoms, desire to smoke, and cravings), beliefs about and reasons for using ENDS (cost, gateway use, health and safety, quitting or reducing tobacco use, restrictions on smoking, satisfaction, similarity to regular cigarettes, withdrawal symptoms, desire to smoke, and cravings, other beliefs);  Study design: a total of 25 studies used cross-sectional or repeated cross-sectional surveys, eight were laboratory experiments, five were case reports, four were observational, three were prospective trials, two used qualitative interviews and two used focus groups. |
| **Glasser et al. (2019)(15)** | Databases searched: PubMed;  A search of the published literature on e-cigarettes was conducted through December 31, 2017, using the following search terms: “e-cigarette*” OR “electronic cigarette” OR “electronic cigarettes” OR “electronic nicotine delivery” OR “vape” OR “vaping.” Additional articles were reviewed based on targeted searches and expert recommendation. | Eligible publications consisted of experimental studies, quasi-experimental studies, observational studies, case reports, case series, qualitative studies, mixed methods, and preclinical/animal studies providing empirical data on e-cigarettes. Publications were restricted to English-language articles published in peer-reviewed international journals. Additional inclusion criteria required that each study: (1) includes a sample of youth (up to age 18 yrs) or young adults (18 to 29 yrs), (2) is longitudinal in design, and (3) assesses e-cigarette use and cigarette smoking at baseline and cigarette smoking at follow-up. Studies with a full adult sample with results stratified by age to distinguish young adults were also included. | Study selection methodology not provided;  Study data were extracted by two reviewers. The first conducted primary data extraction, and the second checked the accuracy of the extraction. | 26 publications | N/A | No | Study setting and duration: US, Scotland, England, Canada, Mexico, The Netherlands, China; follow-up ranged from 6 months to approximately 4 yrs;  Population and participant characteristics: youth or young adults; ten studies included samples of youth or young adults who were never smokers at baseline, five included samples of smokers and never smokers at baseline stratifying results by these two groups (each study is reported in two separate sections of this review), five included samples of smokers and never smokers at baseline analyzed together, and six included a sample of only smokers;  Interventions: e-cigarettes;  Comparators: e-cigarette non-users; e-cigarette infrequent users; those who had not used e-cigarettes in past 30 days; non-nicotine e-cigarettes; NRT; prescriptions (Zyban or Chantix); support from cessation group, Internet, or phone resource; herbal strategy;  Outcomes: combustible cigarette initiation, combustible cigarette cessation;  Study design: longitudinal observational studies. |
| **Harrell et al. (2014)(16)** | Databases searched: PubMed and Web of Science;  The most recent search was conducted on November 20, 2013. Search terms used included “electronic cigarette,” “electronic cigarettes,” “e-cig*,” and “electronic nicotine delivery”. | Inclusion criteria: articles with empirical data related to safety or tobacco cessation;  Reasons for exclusions were: article was not relevant; opinion/commentary articles; review articles; article provided empirical data regarding e-cigarette prevalence; and article provided empirical data not directly relevant, such as media exposure or effects of e-cigarettes on withdrawal symptoms or cognition. | Not provided | 55 studies | N/A | No | Study setting and duration: US, Japan, Singapore, UK, France, Switzerland, China, Poland, Korea, Greece, South Africa, Germany, Italy, New Zealand; study duration not specified in the review for all studies, ranged from 1 week to 52 weeks where specified;  Population and participant characteristics: not specified in the review;  Interventions: e-cigarettes;  Comparators: nicotine patch, non-nicotine e-cigarette, unlit cigarette, cigarettes;  Outcomes: safety and tobacco cigarette cessation;  Study design: case series, within-subjects design, mixed within subjects between subjects, prospective pilot study, survey, RCT, case report. |
| **Patil et al. (2020)(17)** | Databases searched: PubMed, EMBASE, Scopus, and Web of Science;  An online automated literature search was performed from January 1990 up to and including September 2018. Google Scholar, ProQuest, and OpenGrey were additionally searched for the grey literature. Manual reference lists searches of all included articles were also performed to identify any potential relevant articles. Various combinations of descriptors extracted from Medical Subject Headings (MeSH) and free terms were used; “Electronic Cigarettes” [MeSH] OR “Cigarette, Electronic” [MeSH] OR “Cigarettes, Electronic” [MeSH] OR “Electronic Cigarette” [MeSH] OR “E‐Cigarettes” [MeSH] OR “E Cigarettes” [MeSH] OR “E‐Cigarette” [MeSH] OR “E‐Cigs” [MeSH] OR “E Cigs” [MeSH] OR “E‐Cig” [MeSH] AND “Cessation, Tobacco Use” [MeSH] OR “Cessations, Tobacco Use” [MeSH] OR “Tobacco Cessation” [MeSH] OR “Cessation, Tobacco” [MeSH] OR “Smokeless Tobacco Cessation” [MeSH] OR “Cessation, Smokeless Tobacco” [MeSH] OR “Cessation, Smoking” [MeSH] OR “Smoking Cessations” [MeSH] OR “Stopping Smoking” [MeSH] OR “Smoking, Stopping” [MeSH] OR “Giving Up Smoking” [MeSH] OR “Smoking, Giving Up” [MeSH] OR “Smokings, Giving Up” [MeSH] OR “Up Smoking, Giving” [MeSH] OR “Quitting Smoking” [MeSH] OR “Smoking, Quitting” [MeSH]. | Inclusion criteria: The PICOS (Population, Intervention, Comparison, Outcomes, Studies) framework was used to address the focused question, of which: P) Patients who use tobacco; I) e‐cigarette use; C) Patients with no history of e‐cigarette use; O) e‐cigarette use resulted in tobacco cessation; and S) clinical trials and/or observational studies. Clinical trials (randomized controlled trial) and/or observational studies (case‐control, cross‐sectional or population‐based) that recruited patients who used e‐cigarettes to stop their tobacco habits were included. Only English language articles were included;  Exclusion criteria: (a) studies that did not report e‐cigarette use among their sample population; (b) reviews, case‐reports, short communications, experimental studies, and personal opinions, letters to the editor, and conference abstracts. | The study selection was completed in a two‐step process. First, two independent reviewers screened the titles and abstracts of all identified articles using a standardized guide. Subsequently, full texts of studies that met the eligibility criteria were retrieved by both reviewers (using a standardized and pilot tested form) independently reviewing the articles to be included in the study. In the case of disagreements, mutual consensus was made before inclusion of the article;  Tobacco cessation was considered a primary outcome for this systematic review. Both self‐reported abstinence and/or biochemically confirmed methods of abstinence regardless of the duration of abstinence were classified as tobacco cessation. Two reviewers independently collected the data on study location (author, year of study, and country), design, sample population, exposure type and frequency of e‐cigarette use, statistical findings, and conclusions | 13 studies | N/A | No | Study setting and duration: the majority of studies were carried out in USA, one study each was carried out in UK and Italy, while one study was a multi‐center study;  Population: adult current or former cigarette smokers;  Intervention: e-cigarettes;  Comparators: current non-users of e-cigarettes, never users of e-cigarettes, individuals who had only tried e-cigarettes no more than two times, individuals answering “will never use e-cigarettes,” no smoking cessation aid use;  Outcomes: cigarette smoking cessation (self-reported quitting, self-reported since previous study wave, self-reported 30-day abstinence, self-reported 7-day abstinence, biochemical verification of past 7-day abstinence);  Study design: all of the studies included in the systematic review were longitudinal cohort studies. |
| **Meernik et al. (2019)(18)** | Databases searched: PubMed, Embase, PsycINFO, and CINAHL;  An online automated literature search was performed for studies published and indexed between 4 April 2016 and 12 March 2018. Boolean language were used to connect variants of words related to tobacco products, use and flavor for PubMed, which was translated to match the search string requirements for other databases. | Inclusion criteria: observational and experimental studies that assessed the impact of non-menthol flavors in e-cigarettes on perceptions and use behaviors such as initiation, preference and cessation. Studies included populations of any age, race, sex, ethnicity or country.  Exclusion criteria: not English-language; not peer-reviewed (eg, dissertations, technical reports); did not contain original data about flavored e-cigarettes (eg, editorials, commentaries, literature reviews); did not address the impact of flavors on e-cigarette perceptions and use behaviors (eg, biological/medical/chemical toxicology/animal studies, sales trends, effects of flavor bans); related to smoking marijuana or limited findings to menthol flavored e-cigarettes only; qualitative studies; studies examining menthol flavor only. | Two authors reviewed the titles and abstracts of all articles; a third author resolved any discrepancies. Subsequently, two authors reviewed the full text of all 114 articles eligible for full-text screening; a third author resolved any discrepancies. Articles that addressed e-cigarettes from the original systematic review were then added to the articles identified from this current review.  For the articles identified in the most current review, three authors independently extracted data using a data extraction sheet, which assessed study aim, type of flavored tobacco product, characteristics of study populations and study design, and main results and findings related to the impact of flavors in tobacco products. A validated quality assessment tool (QATSDD) was used to examine the quality of quantitative studies with a diverse range of research designs.  To ensure agreement in data extraction and quality assessment, three authors reviewed and extracted the same three articles, then compared results of review and extraction, resolving discrepancies through an iterative approach of discussion. Once mutual standards were decided upon based on this process, each of the three authors then split up the remainder of articles to extract and assess on their own. Evidence tables were created, using pertinent information extracted from each study, and grouped by outcome measures. A similar procedure was conducted in the previous review, and all data were combined for final data analysis. | 51 studies | N/A | No | Seventeen studies were published up to 2016 and 34 published between 2016 and 2018. Most studies included adults only (n=30), though 13 included youth and 8 included both youth and adults.  Most youth were defined as anyone below age 18 years, and most adults were defined as 18 years or older. Additionally, though young adults are an important population and were included as a separate age group in some studies in the review, the variability in definitions of this age group made it difficult to separate for purposes of the results (some defining as ages 19–34 years, some as ages 18–29 years, etc.), and we therefore included all young adults in the adult category.  Seventy-two per cent of included studies were conducted in the USA. While four studies used longitudinal designs, most (n=47; 92%) were cross-sectional. |
| **Aladeokin and Haighton (2019)(19)** | Databases searched: PubMed, Medline (via ProQuest), CINAHL, and Scopus.  An online literature search was performed from January 2005 (when e-cigarettes became commercially available in the UK) and May 2018. Search terms were based on the following concepts: adolescents, e-cigarettes, traditional cigarette smoking, and the UK (Supplementary file gives a search string example). Example of search string:  Adolescent* OR Teen* OR Child* OR Student* OR Juvenile* OR Youth* OR Minor* OR Youngster* OR ‘Young people’ OR ‘Young adult’  AND  ‘Electronic Nicotine Delivery Systems’ OR ‘Electronic nicotine delivery device’ OR ‘ENDS’ OR ‘Electronic cigarette’ OR Vap* OR ‘E-hookahs’ OR ‘Hookah pen*’ OR ‘Ecig*’ OR ‘E-pipe*’  AND  Smok* OR ‘Smok* intent*’ OR ‘Increase* smok*’ OR Experimentation OR Initiat* OR Access* OR Opportunity OR Segway OR ‘Lead* to smok*’ OR ‘Inten* to smok*’ OR ‘Will* to smok*’ OR ‘Attitude* to smok*’ OR ‘Smok* behav*’ OR ‘Smok* perception*’ OR ‘Result* in smoke*’  AND  ‘United Kingdom’ OR UK OR Wales OR Welsh OR England OR English OR Britain OR British OR GB OR ‘Great Britain’ OR Scotland OR Scottish OR ‘Northern Ireland’ OR Irish | Inclusion Criteria: PICOS (Population, Intervention, Comparator, Outcome and Study) format was used to structure the search. Searches were made for peer reviewed empirical studies that focused on adolescent (males and females aged 10–19 years) e-cigarette use in the UK that also examined traditional smoking initiation or intention. Any study design was considered as there was no intervention or comparator. Studies were restricted to those written in English as translation services were not available.  Exclusion crtieria: (a) based outside the UK, (b) focus on adults or very young children (<10 years old), (c) examined traditional cigarette use only, and (d) were editorials, opinion articles, or systematic reviews. | All identified references were downloaded into Endnote citation management software where duplicate references were identified and removed. Initial study selection was carried out by the lead author and involved sifting title and abstract against the inclusion and exclusion criteria. In addition, the PubMed search (n=118) was also sifted by the second author and agreement compared using the Kappa coefficient. All full texts of selected studies were obtained and once again sifted by the lead author against the inclusion and exclusion criteria, in order to determine the final studies for inclusion in the review. The first 10% of the second sift was checked by the second author, with any disagreement discussed and resolved collaboratively.  A detailed data extraction table was developed by adapting data extraction tables from similar studies. Data were extracted under the following sub-headings: study year; source; participants (number of ever users of e-cigarettes); location; design; measures; adjusted variables; results; and conclusion. Data extraction was carried out by the lead author, with one full study checked by the second author. | 8 studies | 3 studies | Not reported. | Study setting and duration: all studies were carried out in the UK;  Population: adolescent (10-18 years) ever, current or never cigarette smokers;  Intervention: e-cigarettes;  Comparators: never e-cigarette users;  Outcomes: cigarette smoking cessation (self-reported quitting, self-reported since previous study wave, self-reported 30-day abstinence, self-reported 7-day abstinence, biochemical verification of past 7-day abstinence);  Study design: five of the studies were cross-sectional, and three were longitudinal (prospective). |
| **Khouja et al. (2020)(20)** | Databases searched: PubMed, Embase, Web of Science, Wiley Cochrane Library, Society for Research on Nicotine and Tobacco, and the Society for Behavioral Medicine were conducted (due to member-restricted access, the NIH Tobacco Regulatory Science Conference abstracts couldn’t not be searched, as stated in the protocol).  An online literature search was performed up to 24 November 2018; due to e-cigarettes being a relatively new product, no date restrictions were placed on the search strategy. The list of studies to be included were compared with those included in previous similar reviews to ensure that no relevant studies had been omitted. Studies written in languages other than English (and for which translations were not readily-available) were translated by colleagues and using Google translate. Studies were initially selected for screening using the following search terms within the titles, abstracts or keywords: Cigar* OR Tobacco OR Smok* AND Electronic Cigarette* OR E-Cig* OR Electronic Nicotine Delivery System* OR Vape OR Vaping OR Alternative Nicotine Delivery System*. Boolean operators and truncations differed depending on the database. Relevant MeSH terms were included when searching the PubMed database: (Tobacco Use[mesh] OR Tobacco[mesh] OR Tobacco use disorder[mesh] OR Tobacco Products[mesh] OR Cigar*[tiab] OR Tobacco[tiab] OR Smok*[tiab]) AND (Electronic Cigarettes[mesh] OR (Nebulizers and Vaporizers[mesh] AND (Tobacco[mesh] OR Tobacco[tiab] OR Nicotine[mesh] OR Nicotine[tiab])) OR Electronic Cigarette*[tiab] OR E-Cig*[tiab] OR Electronic Nicotine Delivery System*[tiab] OR Vape[tiab] OR Vaping[tiab] OR Alternative Nicotine Delivery System*[tiab]) | Inclusion criteria: Randomized controlled trials, longitudinal studies, cross-sectional studies and case–control studies; studies investigating young people aged up to the age of 30 years old (inclusive); studies with a baseline or retrospective measure of e-cigarette use (including but not limited to ever, occasional, heavy, recent, regular or frequent use) prior to initiating smoking and a measure of cigarette smoking (including but not limited to ever, occasional, heavy, recent, regular, frequent or escalated smoking) as an outcome; studies with a comparator group which could include young people who were never, former trier or non-current e-cigarette users or cigarette smokers, dependent on the study.  Exclusion criteria: Review articles and animal studies. | Study selection and data extraction took place over three stages: Stage 1 consisted of title and abstract screening; Stage 2 consisted of a full-text screening; and Stage 3 consisted of data extraction from selected studies.  Titles, abstracts and full-text articles were double screened and then double extracted by three reviewers (first review, 100% of studies; second reviewer, 50% of studies, and third reviewer, 50% of studies). Discrepancies were resolved by a third reviewer where necessary. Covidence (www. covidence.org), an online systematic review tool that is in partnership with Cochrane, was used to streamline and document this process. When insufficient information was available to determine eligibility, study authors were contacted; where insufficient information was provided or obtained, the text was excluded from the review.  For each paper, administrative details, study details, and participant characteristics were extracted, to include: author names; year of publication; country of the study; study design; study name (if applicable); sex of included participants, percentage of males included in the total sample and in the case and control groups; number of cases, controls and the size of the cohort; year(s) of data collection; age of the total sample, cases and controls; follow up length (if applicable); comparison group; exposure; outcome; covariates; definition of e-cigarette use and smoking; and type of assessment of e-cigarette use and smoking.  Exposure and control details, outcome details, and results and conclusions were also extracted, to include: stratification information; direction of effect; effect estimate reported; number of individuals included in specific analyses; number of individuals exposed and unexposed in the analysis and number of subsequent smokers for each group; effect size, confidence intervals, standard errors and p-values for both unadjusted and adjusted analyses; and the conclusion regarding support for the gateway hypothesis. | 24 studies | 17 studies | Not reported. | Study setting and duration: The majority (n = 10) of the studies were conducted in the USA; 3 studies were based in the UK, 1 in Canada, 1 in Mexico, 1 in Germany and 1 in the Netherlands. Follow-up periods ranged from 4 to 24 months.  Population: Participants were predominantly under the age of 18 years (many studies were school-based).  Intervention: ever e-cigarette use; current e-cigarette use  Comparators: never e-cigarette use; non-current e-cigarette use  Outcomes: ever use of cigarettes; experimentation with cigarette smoking; frequent and infrequent cigarette smoking; and current cigarette smoking.  Study design: The majority of included studies were longitudinal; one was cross-sectional in which participants were asked questions regarding their product use retrospectively. |

CINAHL = cumulative index to nursing and allied health literature; e-cigarette = electronic cigarette; ENDS = electronic nicotine delivery system; ENNDS = electronic non-nicotine delivery system; FDA = Food and Drug Administration; h = hour; MeSH = medical subject headings; mg = milligram; min = minute; N/A = not applicable; NIH = National Institutes of Health; NRT = nicotine replacement therapy; PRISMA = preferred reporting items for systematic reviews and meta-analyses; RCT = randomized controlled trial; RoB = risk of bias; US = United States; yr = year

**APPENDIX 8: Risk of Bias, Statistical Analysis, and Heterogeneity Methodology of Included Systematic Reviews.**

| **Author (Publication year)** | **Methods of assessing risk of bias** | **Methods of statistical analysis, if applicable** | **Methods for assessing heterogeneity** | **Methods for assessing publication bias and discussion of its impact on results** |  |
| --- | --- | --- | --- | --- | --- |
| **Lam and West (2015)(1)** | To assess for the RoB in included studies, the Cochrane Collaboration RoB evaluation framework was used. Publications were individually appraised by each author in consideration of six factors: adequate sequence generation to ensure that each study allocated their study groups with a well-known sequence generation process, allocation concealment was sufficient to determine that participants could not foresee their group assignment, proper treatment blinding technique mitigating influences that would impact data outcomes, accurate reporting of incomplete data, outcome reporting that ensured researchers did not selectively report their data and evaluation of any other potential threats to validity. Quality assessment was determined solely by what was reported in each study. No attempt was made to contact authors for missing information. | N/A | Not performed | Not performed | |
| **Franck et al. (2014)(2)** | The authors used the Cochrane Collaboration tool for assessing RoB to determine the quality of included RCTs. This tool assesses the risk of selection bias, performance bias, detection bias, attrition bias, reporting bias, and other biases. Each trial is categorized on the basis of criteria determining the likelihood of potential threats to validity. Quality assessment was performed independently by two reviewers. Quality assessment was performed for four studies. The three nonrandomized studies were not evaluated with Cochrane criteria because they were uncontrolled and therefore of relatively poor quality. | N/A | Not performed | Not performed | |
| **Gentry et al. (2019)(3)** | RCTs/cluster RCTs would have been appraised using the Cochrane RoB tool, although none were identified. Other quantitative studies were assessed using the Effective Public Health Practice Project criteria, and qualitative studies using the Critical Appraisal Skills Programme checklist, by one reviewer, and a sample (four studies) double checked by a second reviewer with no discrepancies. Results were used to inform narrative synthesis. | N/A | Not performed | Not performed | |
| **Hartmann-Boyce et al. (2016)(4)** | Cochrane Handbook for Systematic Reviews of Interventions RoB tool. | For dichotomous data, authors used a ﬁxed-effect Mantel-Haenszel model to calculate the RR with a 95% CI, according to the standard methods of the Cochrane Tobacco Addiction Group for cessation studies. | Heterogeneity was assessed by calculating the *I*^2^ statistic (>50% considered evidence of heterogeneity). | Following standard Cochrane methodology, used the five GRADE considerations (study limitations, consistency of effect, imprecision, indirectness, and publication bias). Impact on results was not discussed. The authors planned to generate funnel plots, but there were insufficient studies to do so. | |
| **El Dib et al. (2017)(5)** | Modified version of the Cochrane Collaboration's instrument that includes nine domains: adequacy of sequence generation, allocation sequence concealment, blinding of participants and caregivers, blinding of data collectors, blinding for outcome assessment, blinding of data analysts, incomplete outcome data, selective outcome reporting, and the presence of other potential sources of bias not accounted for in the previously cited domains. | All outcomes were analyzed as dichotomous variables. In three-arm studies, results from arms judged to be sufficiently similar were combined. Evidence from RCTs was synthesized separately from evidence from cohort studies. For RCTs, pooled Mantel-Haenszel RRs and 95% CIs were calculated using random effects models. Participants with missing data were excluded. Sensitivity analyses were conducted to test robustness of results if they achieved or approached significance. | *I*^2^ statistic and the p-value for the χ^2^ test of heterogeneity. | GRADE analysis; planned to assess through visual inspection of funnel plots, but insufficient number of studies. | |
| **Soneji et al. (2017)(6)** | ROBINS-I tool, which considers biases from confounding, selection of participants into the studies, missing data, and measurement of outcomes. Two investigators evaluated each study. | The authors calculated the observed probability of cigarette smoking initiation among baseline never cigarette smokers by their baseline e-cigarette use. They then calculated the corresponding unadjusted OR using data across all included studies. Next, they estimated the pooled OR for cigarette smoking initiation among baseline ever e-cigarette users compared with never e-cigarette users by fitting a random-effects meta-analysis model. The meta-analysis model included the multivariable regression results of each study that adjusted for known demographic, psychosocial, and behavioral risk factors for cigarette smoking.  Similarly, the authors calculated the observed probability of past 30-day (“current”) cigarette smoking at follow-up among baseline noncurrent cigarette smokers by their baseline use of e-cigarettes in the past 30 days. They then fit a random-effects meta-analysis model to estimate the pooled OR for current cigarette smoking at follow-up among baseline noncurrent cigarette smokers who used e-cigarettes in the past 30 days compared with baseline noncurrent cigarette smokers who did not use e-cigarettes in the past 30 days. The meta-analysis model also included the multivariable regression results of each study that adjusted for known risk factors for cigarette smoking. | *I*^2^ statistic;  For cigarette smoking initiation analysis, the authors performed a subgroup analysis between the following subgroups: adolescent vs young adult studies, baseline year of the study, and regional vs national sample. | Sensitivity analysis to assess the influence of selection bias on the pooled adjusted OR estimated by fitting a Copas selection model. | |
| **McRobbie et al. (2014)(7)** | Cochrane Handbook for Systematic Reviews of Interventions RoB tool, which uses a domain-based evaluation that addresses seven different areas: random sequence generation, allocation concealment, blinding of participants and providers, blinding of outcome assessment, incomplete outcome data, selective outcome reporting, and other potential sources of bias.  Two review authors independently assessed the RoB for each included study. Disagreements were resolved by discussion with a third author. | Where appropriate, data were pooled in meta-analyses. For dichotomous data, a fixed-effect Mantel-Haenszel model was used to calculate the RR and 95% CI. Random-effects model had been planned if there was significant heterogeneity, but there was not. There were insufficient data to calculate the summary estimates for continuous outcomes. Similarly, there was insufficient data to provide pooled analyses of adverse events. | *I*^2^ statistic;  Subgroup analyses were planned; however, there were too few studies to conduct such analyses. | Following standard Cochrane methodology, used the five GRADE considerations (study limitations, consistency of effect, imprecision, indirectness, and publication bias). Impact on results was not discussed. The authors planned to generate funnel plots, but there were insufficient studies to do so. | |
| **Liu et al. (2018)(8)** | Regarding quality assessment, non-RCTs were assessed with the Newcastle-Ottawa Scale, and RCTs were assessed using a CONSORT 2010 statement. Blinded quality assessments of the included literature were performed by 2 researchers, and a third researcher was consulted for the final grading if a discrepancy was noted. | Due to anticipated heterogeneity, the authors used a random-effects model to account for both within and between study heterogeneity. | *I*^2^ statistic | Funnel plots | |
| **Malas et al. (2016)(9)** | To accommodate the broad scope and methodological heterogeneity of the literature, references were assessed using a version of the QualSyst tool, which was modified for the review by merging the quantitative and qualitative checklists and revising criteria based on guidelines from the Cochrane Handbook. The resulting tool evaluated 16 indicators of reporting quality, study design and methodology, sample representativeness, instrument validity/reliability, statistical analysis, reflexivity, and RoB. Quality was assessed by one reviewer; when a quality rating was overruled, a second, blinded reviewer provided a second assessment. Conflict of interest was appraised separately but informed reviewers’ quality assessments; a separate form on DistillerSR was created to document conflict of interest, while a question on the quality assessment form was used to evaluate the potential influence of a present researcher bias. Disagreements and final scores/ratings were decided through inter-reviewer discussion. | N/A | Not performed | Not performed | |
| **Khoudigian et al. (2016)(10)** | The reviewers independently assessed RoB using the Cochrane Collaboration's RoB Tool. This tool assesses the methodological quality of RCTs, assigning low, unclear, or high RoB for the following domains: random sequence generation, allocation concealment, blinding of participants and personnel, blinding of outcome assessor, incomplete outcome data, selective reporting, or other sources of bias (e.g., possible funding by industry). The methodological quality of controlled before-and-after studies was assessed using the same criteria as RCTs except that the “random sequence generation” and “allocation concealment” domains were both reported as “high risk of bias” by both reviewers based on the Cochrane guidelines. Disagreements were resolved through discussion and consensus, and consultation with a third party when needed. Agreement was measured with the κ statistic and its 95% CI. | The pooled result of dichotomous outcomes was summarized using a relative risk and its 95% CI with the Mantel–Haenszel method. A random effect model was used to conduct the meta-analyses, since some heterogeneity exists between studies, such as the length of follow-up and the study design. For all statistical tests, a signiﬁcance level of 5% was used. In the case of continuous outcomes, the pooled data were summarized using a mean difference and its 95% CI. In studies where the SD was missing and not reported, Buck’s regression was used to meta-analyze the data. Studies reporting mean values with no SDs or median with IQR were not included in the meta-analysis and instead summarized narratively. A Poisson distribution was assumed to convert the number of adverse events into an average number of events experienced per subject in each study arm and their corresponding SDs. | *I*^2^ statistic;  In the case of moderate heterogeneity (*I*^2^ = 30–60%) or higher, a priori subgroup analyses were conducted, if feasible, in an attempt to explain the observed heterogeneity. | Not performed | |
| **Kalkhoran and Glantz (2016)(11)** | RoB was assessed using a modification of the ACROBAT-NRSI tool for observational studies and the Cochrane RoB Tool for clinical trials. | The authors computed pooled estimates of the odds of smoking cessation among smokers using e-cigarettes compared with smokers not using e-cigarettes using a random effects meta-analysis with the metan command in Stata version 13.0. Adjusted ORs were used when available, with unadjusted ORs for the remaining studies in the meta-analysis.  The authors performed a sensitivity analysis of the effects of study type (real world vs clinical), longitudinal vs cross-sectional data analysis, sample frame (smokers interested in quitting vs all smokers), control group (NRT users vs all no-e-cigarette users), study population (mental illness or no mental illness), whether the study controlled for level of nicotine dependence, time of e-cigarette assessment (whether e-cigarette use was assessed at baseline or follow-up in longitudinal studies), whether abstinence was biochemically defined, and whether the definition of e-cigarette use was current (past 30-day) use vs ever-use or not within the past 30 days on the results using separate random effects meta-regressions with each factor entered as a dummy variable with the Stata metareg command. The authors considered the nine sensitivity analyses to be a family of comparisons and controlled for multiple comparisons using the Holm-Sidak method to obtain adjusted p-values. | *I*^2^ statistic | Funnel plots and Egger's test | |
| **Rahman et al. (2015)(12)** | The quality of the studies included in the meta-analyses was assessed at the entire study level as well as at the outcome level, in the manner recommended by the Cochrane Collaboration using the Downs and Black instrument. For RCTs, the checklist from the Cochrane Back Review Group was used, while the checklist provided by Downs and Black was used to assess RoB in the observational studies. These tools were simultaneously used to make an assessment of the RoB affecting the findings of the studies. The authors also examined whether all outcomes were reported and reasons behind any exclusion. The quality of the studies was independently assessed by three researchers. The degree of agreement between researchers was calculated together with Cohen’s κ coefficient to measure inter-rater agreement. | Meta-analyses were performed using the metan Stata statistical program and were conducted with two objectives: 1) to compare the effect of e-cigarettes with and without nicotine on abstinence from tobacco smoking in order to evaluate the device’s associated placebo effect, and 2) to evaluate the long-term association between the use of e-cigarettes (i.e., after at least 6 months use) and smoking cessation. This was investigated by calculating a pooled proportion of quitters as reported in the meta-analyzed studies. Subjects lost to follow-up were counted as smokers (consistent with intention-to-treat methods).   Findings from the two included RCTs were meta-analyzed using a Mantel-Haenszel fixed effects model. The pooled RR with 95% CI was calculated. The RR was defined as the ratio of risk of abstinence from smoking among those exposed to nicotine enriched e-cigarettes and the risk of abstinence among those using non-nicotine enriched e-cigarettes. For the second objective, an overall proportion of abstinence from the set of proportions reported in each of the six included studies was calculated using a random-effects meta-analysis model using DerSimonian and Laird method. This method incorporates an estimate of the between-study variation into both the study weights and the standard error of the estimate of the common effect. The precision of an estimate from each included study was represented by the inverse of the variance of the outcome pooled across all participants. Less precise estimates have larger variances, so the inverse of variance is smaller for studies with less precise estimates. The fixed effects model was utilized when running the sub-analyses by study designs. The pooled effect size (estimated by the pooled proportion) with 95% CI was calculated. | *I*^2^ statistic | Not performed | |
| **Gualano et al. (2015)(13)** | The methodological quality of the studies was assessed according to the Newcastle–Ottawa Scale, an 8-item scale designed to rate the quality of the observational studies, and to the JADAD Scale that was speciﬁcally developed to assess the validity of the experimental studies. | N/A | Not performed | Not performed | |
| **Pepper and Brewer (2014)(14)** | The authors relied on the expanded Campbell approach to assess study quality, focusing on factors that bear on internal validity (study design) and external validity (sampling). | N/A | Not performed | Not performed | |
| **Glasser et al. (2019)(15)** | The two reviewers assessed quality for each study according to criteria listed for observational studies by the NHLBI. | N/A | Not performed | Not performed | |
| **Harrell et al. (2014)(16)** | Not performed | N/A | Not performed | Not performed | |
| **Patil et al. (2020)(17)** | Risk of bias was using the NOS. Study quality was assessed in 3 domains: selection, comparability, and outcome/exposure; based on the parameters in each domain, studies were given a maximum of four stars, two stars, and four stars in each domain, respectively. Total score translated to a study quality grade of “Good,” “Fair,” or “Poor”; a score of ≥7 was considered a “Good”. | N/A | Not performed | Not performed | |
| **Meernik et al. (2019)(18)** | Not performed | N/A | Not reported | Not performed | |
| **Aladeokin and Haighton (2019)(19)** | Risk of bias assessment was carried out using ROBINS-I. The following confounding factors were considered important: demographic characteristics; socioeconomic status; family and friends smoking pattern; educational attainment; and other risk behaviors. Quality assessment and risk of bias was carried out by the first author and checked by the second author. | A meta-analysis was carried out among those included studies that were homogenous in terms of design and outcomes. | Chi-squared test (presence of heterogeneity)  *I*^2^ statistic (extend of heterogeneity) | Not performed; however, briefly noted that only published peer-reviewed studies were included (with the aim of identifying evidence of high quality), which may have introduced publication bias. | |
| **Khouja et al. (2020)(20)** | Risk of bias was assessed using the NOS. Selection, comparability and outcome domains were used to assess the risk of bias in all full texts included in the review. Studies were rated as good, fair, or poor quality based on a star system (maximum of 9 stars). Quality/risk was double assessed by the review team. Studies were not excluded based on the risk of bias.  Risk of bias across studies was assessed using the symmetry and 95% confidence region of a funnel plot. Asymmetry and >5% of points lying above the 95% confidence region may have indicated some bias across studies. | In a random-effects model, pooled ORs were calculated from unadjusted and adjusted ORs for ever cigarette use at follow-up among baseline never smokers (who were either baseline ever compared with never e-cigarette users). Where multiple exposure or outcome measures were included in the original study, this estimate was used in the main analysis. If ever use of e-cigarettes was not reported, then the main effect reported in the study was included in the main analyses.   Where possible, results were also analyzed in a series of subgroups: ever versus never e-cigarette use at baseline and ever versus never smoking at follow-up; ever versus never e-cigarette use at baseline and current (past 30 days) versus non-current use of cigarettes at follow-up; and current versus non-current e-cigarette use at baseline and ever versus never smoking at follow-up. | *I*^2^ statistic; sources of heterogeneity explored via subgroup analysis. | Funnel plots | |

ACROBAT-NRSI = a Cochrane Risk of Bias Assessment tool for non-randomized studies of interventions; CI = confidence interval; e-cigarette = electronic cigarette; GRADE = grading of recommendations, assessment, development, and evaluations; IQR = interquartile range; NHLBI = National Heart, Lung, and Blood Institute; NOS = Newcastle-Ottawa Scale; NRT = nicotine replacement therapy; OR = odds ratio; RCT = randomized controlled trial; RoB = risk of bias; ROBINS-I = risk of bias in non-randomized studies – of interventions; ROBINS-I= Risk Of Bias In Non-randomized Studies of Interventions; RR = risk ratio; SD = standard deviation; vs = versus

**APPENDIX 9: Scoring Results of Methodological Quality Using the AMSTAR 2 Tool.**

| **Item** | **n (%)**  N = 16 | **n/N (adjusted %)*** |
| --- | --- | --- |
| 1. Did the research questions and inclusion criteria for the review include the components of PICO?  **Yes**  **No** | **15 (75.0%)**  **5 (25.0%)** |  |
| 2. Did the report of the review contain an explicit statement that the review methods were established prior to the conduct of the review and did the report justify any significant deviations from the protocol?  **Yes**  **Partial yes**  **No** | **4 (20.0%)**  **2 (10.0%)**  **14 (70.0%)** |  |
| 3. Did the review authors explain their selection of the study designs for inclusion in the review?  **Yes**  *Explanation for including only RCTs*  *Explanation for including only NRSIs*  *Explanation for including both RCTs and NRSIs*  **No** | **9 (45.0%)**  *0*  *0*  *9 (45.0%)*  **11 (55.0%)** |  |
| 4. Did the review authors use a comprehensive literature search strategy?  **Yes**  **Partial yes**  **No** | **0**  **11 (55.0%)**  **9 (45.0%)** |  |
| 5. Did the review authors perform study selection in duplicate?  **Yes**  *At least two reviewers independently agreed on selection of eligible studies and achieved consensus on which studies to include*  *OR two reviewers selected a sample of eligible studies and achieved good agreement (at least 80 percent), with the remainder selected by one reviewer*  **No** | **12 (60.0%)**  *10 (50.0%)*  *2 (10.0%)*  **8 (40.0%)** |  |
| 6. Did the review authors perform data extraction in duplicate?  **Yes**  *At least two reviewers achieved consensus on which data to extract from included studies*  *OR two reviewers extracted data from a sample of eligible studies and achieved good agreement (at least 80%), with the remainder extracted by one reviewer*  **No** | **9 (45.0%)**  *9 (45.0%)*  *0*  **11 (55.5%)** |  |
| 7. Did the review authors provide a list of excluded studies and justify the exclusions?  **Yes**  **No** | **2 (10.0%)**  **18 (90.0%)** |  |
| 8. Did the review authors describe the included studies in adequate detail?  **Yes**  **Partial yes**  **No** | **9 (45.0%)**  **8 (40.0%)**  **3 (15.0%)** |  |
| 9. For **RCTs** - Did the review authors use a satisfactory technique for assessing the risk of RoB in individual studies that were included in the review?  **Yes**  **Partial yes**  **No**  **Includes only NRSIs**  For **NRSIs** - Did the review authors use a satisfactory technique for assessing the RoB in individual studies that were included in the review?  **Yes**  **Partial yes**  **No**  **Includes only RCTs** | **9 (45.0%)**  **0**  **5 (25.0%)**  **6 (30.0%)**  **6 (30.0%)**  **3 (15.0%)**  **10 (50.0%)**  **1 (5.0%)** | **9/14 (64.2%)**  **6/19 (31.6%)** |
| 10. Did the review authors report on the sources of funding for the studies included in the review?  **Yes**  **No** | **3 (15.0%)**  **17 (85.0%)** |  |
| 11. For **RCT**s - If meta-analysis was performed did the review authors use appropriate methods for statistical combination of results?  **Yes**  **No**  **Not applicable (no meta-analysis conducted or NRSIs only)**  For **NRSIs** - If meta-analysis was performed did the review authors use appropriate methods for statistical combination of results?  **Yes**  **No**  **Not applicable (no meta-analysis conducted or RCTs only)** | **6 (30.0%)**  **1 (5.0%)**  **13 (65.0 %)**  **4 (20.0%)**  **4 (20.0%)**  **12 (60.0%)** | **6/7 (85.7%)**  **4/8 (50.0%)** |
| 12. If meta-analysis was performed, did the review authors assess the potential impact of RoB in individual studies on the results of the meta-analysis or other evidence synthesis?  **Yes**  *included only low risk of bias RCTs*  *OR if the pooled estimate was based on RCTs and/or NRSIs at variable RoB, the authors performed analyses to investigate possible impact of RoB on summary estimates of effect*  **No**  **No meta-analysis conducted** | **6 (30.0%)**  *3 (15.0%)*  *3 (15.0%)*  **4 (20.0%)**  **10 (50.0%)** | **6/10 (60.0%)** |
| 13. Did the review authors account for RoB in individual studies when interpreting/ discussing the results of the review?  **Yes**  *Included only low risk of bias RCTs*  *OR if RCTs with moderate or high RoB or NRSI were included, the review provided a discussion of the likely impact of RoB on the results*  **No** | **12 (60.0%)**  *3 (15.0%)*  *9 (45.0%)*  **8 (40.0%)** |  |
| 14. Did the review authors provide a satisfactory explanation for, and discussion of, any heterogeneity observed in the results of the review?  **Yes**  *There was no significant heterogeneity in the results*  *OR if heterogeneity was present, the authors performed an investigation of sources of any heterogeneity in the results and discussed the impact of this on the results of the review*  **No** | **10 (50.0%)**  *3 (15.0%)*  *7 (35.0%)*  **10 (50.0%)** |  |
| 15. If they performed quantitative synthesis did the review authors carry out an adequate investigation of publication bias (small study bias) and discuss its likely impact on the results of the review?  **Yes**  **No**  **No meta-analysis conducted** | **7 (35.0%)**  **3 (15.0%)**  **10 (50.0%)** | **7/10 (70.0%)** |
| 16. Did the review authors report any potential sources of conflict of interest, including any funding they received for conducting the review?  **Yes**  *The authors reported no competing interests*  *OR the authors described their funding sources and how they managed potential conflicts of interest*  **No** | **18 (90.0%)**  *13 (65.0%)*  *5 (25.0%)*  **2 (10.0%)** |  |
| **OVERALL SCORE**  High  Moderate  Low  Critically Low | **0**  **0**  **2 (10.0%)**  **18 (90.0%)** |  |

NRSI = nonrandomized studies of interventions; PICO = patients, intervention, comparator group, outcome; RCT = randomized controlled trials; RoB = risk of bias

* An “adjusted” percentage was generated where appropriate to eliminate any non-applicable studies from the calculation.

**APPENDIX 10: Summary of Methodological Quality Using the AMSTAR 2 Tool.**

N/A = not applicable; NRSI = nonrandomized studies of interventions; PICO = patients, intervention, comparator group, outcome; RCT = randomized controlled trials; RoB = risk of bias

**APPENDIX 11: Scoring Results of Quality of Reporting Using the PRISMA Tool.**

| **Item** | **n (%)** | **n/N (adjusted %)*** |
| --- | --- | --- |
| *Title* | | |
| 1. Does the title identify the report as a systematic review, meta-analysis, or both?  **Yes**  **Partially met criteria**  **No** | 13 (65.0%)  0  7 (35.0%) |  |
| *Abstract* | | |
| 2. Provide a structured summary including, as applicable: background; objectives; data sources; study eligibility criteria, participants, and interventions; study appraisal and synthesis methods; results; limitations; conclusions and implications of key findings; systematic review registration number.  **Yes**  **Partially met criteria**  **No** | 19 (95.0%)  0  1 (5.0%) |  |
| *Introduction* | | |
| 3. Does this review describe the rationale for the review in the context of what is already known?  **Yes**  **Partially met criteria**  **No** | 20 (100.0%)  0  0 |  |
| 4. Does this review provide an explicit statement of questions being addressed with reference to PICOS?  **Yes**  **Partially met criteria**  **No** | 3 (15.0%)  15 (75.0%)  2 (10.0%) |  |
| *Methods* | | |
| 5. Do they indicate if a review protocol exists, if and where it can be accessed?  **Yes**  **Partially met criteria**  **No** | 7 (35.0%)  2 (10.0%)  11 (55.0%) |  |
| 6. Does this review specify study characteristics (e.g., PICOS, length of follow-up) and report characteristics (e.g., years considered, language, publication status) used as criteria for eligibility, giving rationale?  **Yes**  **Partially met criteria**  **No** | 4 (20.0%)  16 (80.0 %)  0 |  |
| 7. Does this review describe all information sources (e.g., databases with dates of coverage, contact with study authors to identify additional studies) in the search and date last searched?  **Yes**  **Partially met criteria**  **No** | 10 (50.0%)  10 (50.0%)  0 |  |
| 8. Does this review present full electronic search strategy for at least one database, including any limits used, such that it could be repeated?  **Yes**  **Partially met criteria**  **No** | 7 (35.0%)  1 (5.0%)  12 (60.0%) |  |
| 9. Does this review state the process for selecting studies (i.e., screening, eligibility, included in systematic review, and, if applicable, included in the meta-analysis)?  **Yes**  **Partially met criteria**  **No** | 14 (70.0%)  4 (20.0%)  2 (10.0%) |  |
| 10. Does this review describe method of data extraction from reports (e.g., piloted forms, independently, in duplicate) and any processes for obtaining and confirming data from investigators?  **Yes**  **Partially met criteria**  **No** | 6 (30.0%)  10 (50.0%)  4 (20.0%) |  |
| 11. Does this review list and define all variables for which data were sought (e.g., PICOS, funding sources) and any assumptions and simplifications made?  **Yes**  **Partially met criteria**  **No** | 13 (65.0%)  4 (20.0%)  3 (15.0%) |  |
| 12. Does this review describe methods used for assessing risk of bias of individual studies (including specification of whether this was done at the study or outcome level), and how this information is to be used in any data synthesis?  **Yes**  **Partially met criteria**  **No** | 10 (50.0%)  7 (35.0%)  3 (15.0%) |  |
| 13. Does this review state the principal summary measures (e.g., risk ratio, difference in means)?  **Yes**  **Partially met criteria**  **No**  **Not applicable** | 9 (45.0%)  0  11 (55.0%)  0 |  |
| 14. Describe the methods of handling data and combining results of studies, if done, including measures of consistency (such as I^2^) for each meta-analysis.  **Yes**  **Partially met criteria**  **No**  **Not applicable** | 9 (45.0%)  0  2 (10.0%)  9 (45.0%) | 9/11 (81.8%) |
| 15. Does this review specify any assessment of risk of bias that may affect the cumulative evidence (e.g., publication bias, selective reporting within studies)?  **Yes**  **Partially met criteria**  **No** | 10 (50.0%)  0  10 (50.0%) |  |
| 16. Does this review describe methods of additional analyses (e.g., sensitivity or subgroup analyses, meta-regression), if done, indicating which were pre-specified?  **Yes**  **Partially met criteria**  **No**  **Not applicable** | 4 (20.0%)  3 (15.0%)  2 (10.0%)  11 (55.0%) | 3/9 (33.3%) |
| *Results* | | |
| 17. Does this review give numbers of studies screened, assessed for eligibility, and included in the review, with reasons for exclusions at each stage, ideally with a flow diagram?  **Yes**  **Partially met criteria**  **No** | 10 (50.0%)  10 (50.0%)  0 |  |
| 18. Does this review present characteristics for which data were extracted (e.g., study size, PICOS, follow-up period) and provide the citations, for each study presented?  **Yes**  **Partially met criteria**  **No** | 16 (80.0%)  4 (20.0%)  0 |  |
| 19. Does this review present data on risk of bias of each study and, if available, any outcome level assessment (see item 12).  **Yes**  **Partially met criteria**  **No**  **Not applicable** | 12 (60.0%)  4 (20.0%)  1 (5.0%)  3 (15.0%) | 12/17 (70.6%) |
| 20. Does this review present, for each study: (a) simple summary data for each intervention group, and if applicable: (b) effect estimates and confidence intervals, ideally with a forest plot, for all outcomes considered (benefits or harms)?  **Yes**  **Partially met criteria**  **No** | 12 (60.0%)  5 (25.0%)  3 (15.0%) |  |
| 21. Does this review present results of each meta-analysis done, including confidence intervals and measures of consistency?  **Yes**  **Partially met criteria**  **No**  **Not applicable** | 10 (50.0%)  0  0  10 (50.0%) | 10/10 (100%) |
| 22. Does this review present results of any assessment of risk of bias across studies (see Item 15)?  **Yes**  **Partially met criteria**  **No**  **Not applicable** | 7 (35.0%)  3 (15.0%)  1 (5.0%)  9 (45.0%) | 7/11 (63.6%) |
| 23. Does this review give results of additional analyses, if done (e.g., sensitivity or subgroup analyses, meta-regression [see Item 16])?  **Yes**  **Partially met criteria**  **No**  **Not applicable** | 9 (45.0%)  0  0  11 (55.0%) | 9/9 (100%) |
| *Discussion* | | |
| 24. Does this review summarize the main findings including the strength of evidence for each main outcome; consider their relevance to key groups (e.g., healthcare providers, users, and policy makers)?  **Yes**  **Partially met criteria**  **No** | 20 (100.0%)  0  0 |  |
| 25. Does this review discuss limitations at study and outcome level (e.g., risk of bias), and at review-level (e.g., incomplete retrieval of identified research, reporting bias)?  **Yes**  **Partially met criteria**  **No** | 18 (90.0%)  1 (5.0%)  1 (5.0%) |  |
| 26. Does this review provide a general interpretation of the results in the context of other evidence, and implications for future research?  **Yes**  **Partially met criteria**  **No** | 19 (95.0%)  1 (5.0%)  0 |  |
| *Funding* | | |
| 27. Does this review describe sources of funding for the systematic review and other support (e.g., supply of data); role of funders for the systematic review?  **Yes**  **Partially met criteria**  **No** | 11 (55.0%)  5 (25.0%)  4 (20.0%) |  |

PICOS = participants, interventions, comparisons, outcomes, and study design

* An “adjusted” percentage was generated where appropriate to eliminate any non-applicable studies from the calculation.

**APPENDIX 12: Summary of Quality of Reporting Using the PRISMA Tool.**

N/A = not applicable: RoB = risk of bias

**REFERENCES**

1. Lam C, West A. Are electronic nicotine delivery systems an effective smoking cessation tool? Can J Respir Ther. 2015;51(4):93-8.

2. Franck C, Budlovsky T, Windle SB, Filion KB, Eisenberg MJ. Electronic cigarettes in North America: history, use, and implications for smoking cessation. Circulation. 2014;129(19):1945-52.

3. Gentry S, Forouhi NG, Notley C. Are Electronic Cigarettes an Effective Aid to Smoking Cessation or Reduction Among Vulnerable Groups? A Systematic Review of Quantitative and Qualitative Evidence. Nicotine Tob Res. 2019;21(5):602-16.

4. Hartmann-Boyce J, McRobbie H, Bullen C, Begh R, Stead LF, Hajek P. Electronic cigarettes for smoking cessation. Cochrane Database Syst Rev. 2016;9:CD010216.

5. El Dib R, Suzumura EA, Akl EA, Gomaa H, Agarwal A, Chang Y, et al. Electronic nicotine delivery systems and/or electronic non-nicotine delivery systems for tobacco smoking cessation or reduction: a systematic review and meta-analysis. BMJ Open. 2017;7(2):e012680.

6. Soneji S, Barrington-Trimis JL, Wills TA, Leventhal AM, Unger JB, Gibson LA, et al. Association Between Initial Use of e-Cigarettes and Subsequent Cigarette Smoking Among Adolescents and Young Adults: A Systematic Review and Meta-analysis. JAMA pediatrics. 2017;171(8):788-97.

7. McRobbie H, Bullen C, Hartmann-Boyce J, Hajek P. Electronic cigarettes for smoking cessation and reduction. Cochrane Database Syst Rev. 2014(12):CD010216.

8. Liu X, Lu W, Liao S, Deng Z, Zhang Z, Liu Y, et al. Efficiency and adverse events of electronic cigarettes: A systematic review and meta-analysis (PRISMA-compliant article). Medicine (Baltimore). 2018;97(19):e0324.

9. Malas M, van der Tempel J, Schwartz R, Minichiello A, Lightfoot C, Noormohamed A, et al. Electronic Cigarettes for Smoking Cessation: A Systematic Review. Nicotine Tob Res. 2016;18(10):1926-36.

10. Khoudigian S, Devji T, Lytvyn L, Campbell K, Hopkins R, O'Reilly D. The efficacy and short-term effects of electronic cigarettes as a method for smoking cessation: a systematic review and a meta-analysis. Int J Public Health. 2016;61(2):257-67.

11. Kalkhoran S, Glantz SA. E-cigarettes and smoking cessation in real-world and clinical settings: a systematic review and meta-analysis. Lancet Respir Med. 2016;4(2):116-28.

12. Rahman MA, Hann N, Wilson A, Mnatzaganian G, Worrall-Carter L. E-cigarettes and smoking cessation: evidence from a systematic review and meta-analysis. PLoS One. 2015;10(3):e0122544.

13. Gualano MR, Passi S, Bert F, La Torre G, Scaioli G, Siliquini R. Electronic cigarettes: assessing the efficacy and the adverse effects through a systematic review of published studies. J Public Health (Oxf). 2015;37(3):488-97.

14. Pepper JK, Brewer NT. Electronic nicotine delivery system (electronic cigarette) awareness, use, reactions and beliefs: a systematic review. Tob Control. 2014;23(5):375-84.

15. Glasser A, Abudayyeh H, Cantrell J, Niaura R. Patterns of E-Cigarette Use Among Youth and Young Adults: Review of the Impact of E-Cigarettes on Cigarette Smoking. Nicotine Tob Res. 2019;21(10):1320-30.

16. Harrell PT, Simmons VN, Correa JB, Padhya TA, Brandon TH. Electronic nicotine delivery systems ("e-cigarettes"): review of safety and smoking cessation efficacy. Otolaryngol Head Neck Surg. 2014;151(3):381-93.

17. Patil S, Arakeri G, Patil S, Ali Baeshen H, Raj T, Sarode SC, et al. Are electronic nicotine delivery systems (ENDs) helping cigarette smokers quit?-Current evidence. J Oral Pathol Med. 2020;49(3):181-9.

18. Meernik C, Baker HM, Kowitt SD, Ranney LM, Goldstein AO. Impact of non-menthol flavours in e-cigarettes on perceptions and use: an updated systematic review. BMJ Open. 2019;9(10):e031598.

19. Aladeokin A, Haighton C. Is adolescent e-cigarette use associated with smoking in the United Kingdom?: A systematic review with meta-analysis. Tob Prev Cessat. 2019;5:15.

20. Khouja JN, Suddell SF, Peters SE, Taylor AE, Munafo MR. Is e-cigarette use in non-smoking young adults associated with later smoking? A systematic review and meta-analysis. Tob Control. 2020.
